# TDP-43 dysregulation of polyadenylation site selection is a defining feature of RNA misprocessing in amyotrophic lateral sclerosis and frontotemporal dementia

**DOI:** 10.1172/JCI182088

**Published:** 2025-06-02

**Authors:** Frederick J. Arnold, Ya Cui, Sebastian Michels, Michael R. Colwin, Cameron M. Stockford, Wenbin Ye, Vidhya Maheswari Jawahar, Karen Jansen-West, Julien Philippe, Ravinder Gulia, Yunzi Gou, Oliver H. Tam, Sneha Menon, Wendy G. Situ, Saira L. Cazarez, Aryan Zandi, Kean C.K. Ehsani, Sierra Howard, Dennis W. Dickson, Molly Gale Hammell, Mercedes Prudencio, Leonard Petrucelli, Wei Li, Albert R. La Spada

**Affiliations:** 1 Department of Pathology & Laboratory Medicine and; 2 Department of Biological Chemistry, University of California, Irvine, Irvine, California, USA.; 3 Department of Neurology, University of Ulm, Ulm, Germany.; 4 Department of Neuroscience, Mayo Clinic, Jacksonville, Florida, USA.; 5 Institute for Systems Genetics, NYU Langone Health, New York, New York, USA.; 6 Department of Neuroscience & Physiology, NYU Grossman School of Medicine, New York, New York, USA.; 7 Neuroscience Graduate Program, Mayo Graduate School, Mayo Clinic College of Medicine, Jacksonville, Florida, USA.; 8 Department of Neurology,; 9 Department of Neurobiology & Behavior, and; 10 UCI Center for Neurotherapeutics, University of California, Irvine, Irvine, California, USA.

**Keywords:** Genetics, Neuroscience, Neurodegeneration, RNA processing

## Abstract

Nuclear clearance and cytoplasmic aggregation of TAR DNA-binding protein 43 (TDP-43) are observed in many neurodegenerative disorders, including amyotrophic lateral sclerosis (ALS) and frontotemporal dementia (FTD). Although TDP-43 dysregulation of splicing has emerged as a key event in these diseases, TDP-43 can also regulate polyadenylation; yet this has not been adequately studied. Here, we applied the dynamic analysis of polyadenylation from an RNA-Seq (DaPars) tool to ALS/FTD transcriptome datasets and report extensive alternative polyadenylation (APA) upon TDP-43 alteration in ALS/FTD cell models and postmortem ALS/FTD neuronal nuclei. Importantly, many identified APA genes highlight pathways implicated in ALS/FTD pathogenesis. To determine the functional relevance of APA elicited by TDP-43 nuclear depletion, we examined microtubule affinity regulating kinase 3 (MARK3). Nuclear loss of TDP-43 yielded increased expression of *MARK3* transcripts with longer 3′ UTRs, corresponding with a change in the subcellular distribution of MARK3 and increased neuronal tau S262 phosphorylation. Our findings define changes in polyadenylation site selection as a previously understudied feature of TDP-43–driven disease pathology in ALS/FTD and highlight a potentially important mechanistic link between TDP-43 dysfunction and tau regulation.

## Abstract 2

We report that TDP-43 dysfunction affects polyadenyation site selection to alter the length of the 3’-untranslated-region of RNAs to promote neurodegeneration, revealing a novel aspect of TDP-43 RNA pathology.

## Graphical abstract

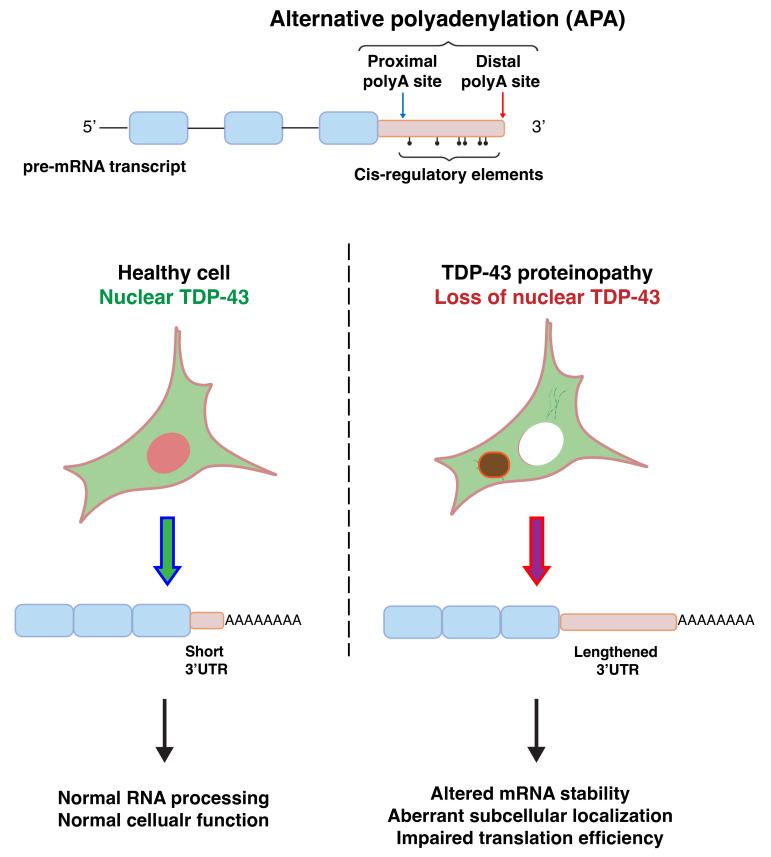

## Introduction

While the underlying etiology of many age-related neurodegenerative disorders remains unknown, distinct neuronal subtypes and specific brain regions exhibit a common pathological hallmark of nuclear clearance and cytoplasmic aggregation of TAR DNA-binding protein 43 (TDP-43) (1). TDP-43 pathology is present in motor neurons in approximately 97% of patients with amyotrophic lateral sclerosis (ALS) and in the neocortex of approximately 45% of individuals suffering from frontotemporal dementia (FTD). Cytoplasmic aggregates of TDP-43 have also been observed in Huntington’s disease (2) and chronic traumatic encephalopathy (3) and are emerging as the defining pathology for limbic-predominant age-related TDP-43 encephalopathy (LATE), a recently described late-onset dementia characterized by atrophy in the medial temporal lobes and frontal cortex, though not exclusive to this brain region (4). Furthermore, as many as 57% of Alzheimer’s disease (AD) patients display TDP-43 copathology, which is often described as LATE neuropathologic change (LATE-NC) (5) and correlates with more rapid disease progression and worse cognitive impairment (6, 7). Consequently, heterogenous neurodegenerative disorders characterized by TDP-43 pathology may collectively be referred to as “TDP-43 proteinopathies” (1).

Under physiological conditions, TDP-43 is a predominantly nuclear RNA-binding protein (RBP) that directly binds over 6,000 RNAs (8). Prior studies of ALS/FTD have identified differentially expressed and alternatively spliced genes associated with TDP-43 nuclear loss of function. This includes a motor neuron growth and repair factor, stathmin-2 (STMN2), in which TDP-43 loss of nuclear function results in derepression of a cryptic 3′ splice site in the *STMN2* gene, favoring inclusion of a cryptic exon (CE) in the *STMN2* pre-mRNA, premature transcription termination, and loss of STMN2 protein expression (9–11). Another gene subject to TDP-43 splicing dysregulation in ALS/FTD is *UNC13A*, as depletion of TDP-43 in the nucleus promotes CE inclusion within the *UNC13A* pre-mRNA, resulting in loss of UNC13A protein expression (12, 13). Both STMN2 and UNC13A are currently under investigation as candidates for therapeutic intervention and biomarker development in ALS/FTD.

In addition to its role in RNA splicing, TDP-43 regulates alternative polyadenylation (APA) by binding target RNAs near polyadenylation signals (PAS) (14), yet this aspect of TDP-43 function remains relatively unexplored in TDP-43 proteinopathies. Notably, up to 70% of all human pre-mRNAs contain more than one polyadenylation (poly[A]) site, and differential PAS utilization yields transcripts with distinct 3′ untranslated regions (3′ UTRs) (15, 16). APA is a highly conserved and ubiquitous mechanism of gene regulation that is exceedingly cell-type specific (17). As it turns out, neurons tend to preferentially utilize distal poly(A) sites (18, 19), accounting for the well-established observation that genes expressed in the CNS encode transcripts containing the longest 3′ UTR sequences of all tissues throughout the body. This suggests that APA is likely an important posttranscriptional regulatory mechanism in neurons and other CNS cell types, as the 3′ UTR is highly enriched for binding motifs for microRNAs (miRNAs) (20) and RBPs (21). Hence, by modulating the presence or absence of such cis-regulatory elements, APA can produce transcript isoforms with substantial differences in RNA stability, subcellular localization, nuclear export, and translational efficiency (22). These changes in RNA metabolism can markedly impact protein expression, though it is also likely that changes in subcellular localization can modulate gene function without affecting steady-state RNA levels. An important consideration for the study of APA is that standard informatics analysis of RNA-Seq data does not fully capture APA events. For this reason, a growing number of computational methods have been developed to infer PAS usage from standard, short-read, bulk RNA-Seq transcriptome data, allowing post hoc analysis of APA from existing datasets (23).

Here, we applied the dynamic analysis of poly(A) from an RNA-Seq (DaPars) tool to transcriptome data sets obtained from neuron cell culture models of ALS/FTD and from ALS/FTD patient postmortem neuron nuclei. We identified hundreds of previously unknown genes regulated by TDP-43, with many genes conserved across multiple cell types and model systems. As a substantial number of these genes function in cellular pathways implicated in ALS/FTD disease pathogenesis, APA analysis can provide insight into how TDP-43 dysfunction alters the transcriptome of neurons and other cell types. To determine the biological relevance of TDP-43-regulated APA, we studied its effects on microtubule affinity regulating kinase 3 (MARK3), a highly significant (*P* = 9.4 × 10^–16^) hit from APA analysis of ALS/FTD frontal cortex neuron nuclei containing or depleted of TDP-43. We confirmed that upon TDP-43 knockdown, the *MARK3* gene displayed increased expression of transcripts with a longer 3′ UTR in induced pluripotent stem cell–derived (iPSC-derived) neurons and an overall increase in transcript abundance in iPSC-derived neurons and in the frontal cortex of patients with frontotemporal lobar degeneration with TDP-43 pathology (FTLD-TDP). We further documented that TDP-43 depletion promoted increased phosphorylation of tau at serine 262, a previously established target of MARK3 and a read-out for tau dysfunction in AD (24). Our results indicate that TDP-43 dysregulation of poly(A) site selection is another facet of the RNA-processing dysfunction that is a central feature of the disease process in TDP-43 proteinopathies.

## Results

### TDP-43 depletion induces widespread APA in neuronal cells.

**Figure 1:**
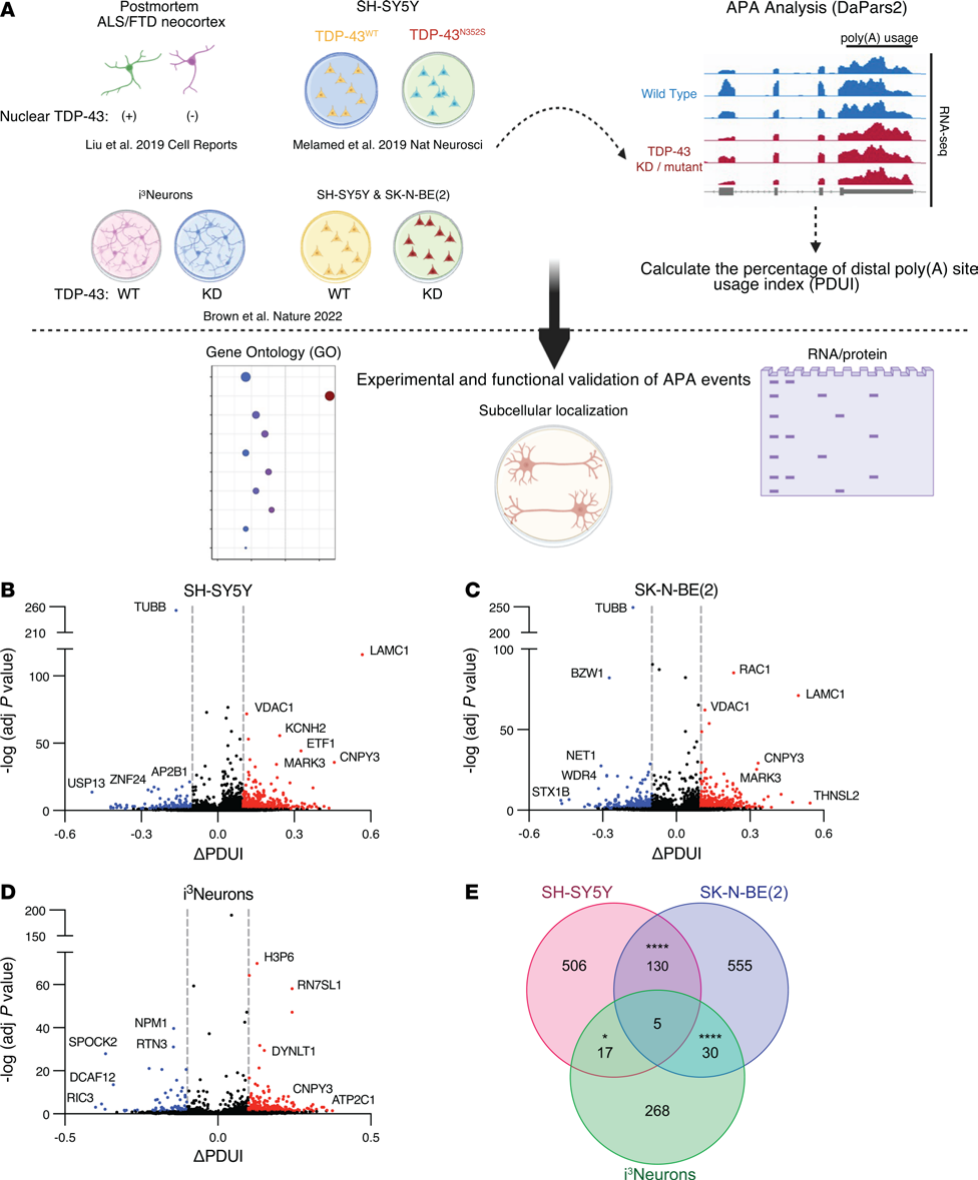
TDP-43 depletion induces widespread APA. (**A**) Overview of the study. We applied the dynamic analysis of poly(A) from an RNA-Seq (DaPars) tool to published RNA-Seq datasets to detect APA events resulting from TDP-43 depletion or mutation in immortalized cell lines, stem cell–derived neurons, and postmortem ALS/FTD neocortex. We then performed experimental and functional validation of select targets. Volcano plots depicting APA events in SH-SY5Y cells (**B**), SK-N-BE(2) cells (**C**), and i^3^Neurons (**D**) in which TDP-43 was knocked down via shRNA treatment. APA genes with FDR adj. *P* < 0.05 and ΔPDUI ≥ 0.1 are depicted in red, and APA genes with FDR adj. *P* < 0.05 and ΔPDUI ≤ –0.1 are depicted in blue. (**E**) Venn diagram illustrating the intersection of APA events across datasets. Hypergeometric distribution test was used to determine the statistical significance of overlapping genes between datasets: SH-SY5Y cells versus SK-N-BE(2) cells, *P* = 1.68 × 10^–51^; SH-SY5Y cells versus i^3^Neurons, *P* = 0.011; SK-N-BE(2) cells versus i^3^Neurons, *P* = 6.01 × 10^–06^.

To identify APA events regulated by TDP-43 in neuronal cells, we employed the dynamic analysis of poly(A) from an RNA-Seq (DaPars) tool to previously published RNA-Seq datasets (Figure 1A). While a number of tools have been developed to quantify APA from bulk RNA-Seq data (23), we selected DaPars (25), which identifies and quantifies de novo poly(A) site usage by applying a linear regression model to localized changes in RNA-Seq read density within the 3′ UTR. Given the established role of TDP-43 in regulating the splicing of unannotated “cryptic” exons, we hypothesized that “cryptic,” unannotated poly(A) sites may be similarly utilized upon TDP-43 depletion or mutation. DaPars calculates the percentage of distal poly(A) site usage index (PDUI) for each transcript, with the results averaged across replicates to determine ΔPDUI in cells where TDP-43 was knocked down or mutated in comparison with control conditions. A positive ΔPDUI denotes increased relative expression of transcripts with a longer 3′ UTR, while a negative ΔPDUI signifies increased relative expression of transcripts with a shorter 3′ UTR.

Using published RNA-Seq data from SH-SY5Y cells, SK-N-BE(2) cells, and i^3^Neurons in which TDP-43 was depleted by shRNA knockdown (12), we identified hundreds of genes in which TDP-43 knockdown resulted in significant APA (FDR adjusted [adj] *P* < 0.05, |ΔPDUI| 0.1) (Figure 1, B–D, Tables 1, 2, and 3, and Supplemental Tables 1–3; supplemental material available online with this article;https://doi.org/10.1172/JCI182088DS1). APA genes exhibit both 3′ UTR lengthening and shortening upon TDP-43 knockdown; however, we observed increased use of distal poly(A) sites in 69.9% (460/658) of APA genes in SH-SY5Y cells, 59.6% (429/720) of APA genes in SK-N-BE(2) cells, and 78.1% (250/320) of APA genes in i^3^Neurons. Nearly 20% of APA events were shared between the closely related SH-SY5Y and SK-N-BE(2) neuroblastoma cell lines (Figure 1E), including 4/10 of the top APA genes by *P* value (Tables 1 and 2). Additionally, 16.3% (52/320) of APA genes in i^3^Neurons overlapped with at least 1 of the immortalized neuronal cell lines (Figure 1E), indicating that, while APA is known to be highly cell-type specific (17), many APA events regulated by TDP-43 are conserved across multiple neuronal cell types.

We also applied DaPars to previously published transcriptome data sets in which TDP-43 was depleted by siRNAs in SH-SY5Y cells (11) or in human embryonic stem cell–derived motor neurons (hESC-MNs) for 96 hours (9) (Supplemental Figure 1 and Supplemental Tables 4 and 5). As expected, we observed fewer APA changes in cells in which TDP-43 was knocked down for a shorter duration or incompletely (~60% in hESC-MNs). Both datasets do, however, exhibit significantly decreased PDUI in *SMC1A*, as previously shown for HEK293 cells upon TDP-43 siRNA knockdown (14).

Given that APA can affect cellular function by regulating mRNA stability and subcellular localization (22), we performed Gene Ontology (GO) analysis of APA genes in each neuronal cell type (Supplemental Figure 2). Shared terms between datasets for biological pathways known to be highly relevant to ALS/FTD included “establishment of protein localization” (SH-SY5Y and i^3^Neurons) and “cytoskeleton organization” (SH-SY5Y and SK-N-BE[2]), and shared terms for GO molecular functions were “kinase activity,” “protein kinase binding,” and “cytoskeletal protein binding” (SH-SY5Y and SK-N-BE[2]). These results indicate that TDP-43 regulation of APA impacts disease-relevant pathways and should be considered, along with differential expression and alternative splicing, as a key aspect of TDP-43 dysfunction.

### TDP-43 binds within the 3′ UTR of a subset of APA genes, preferentially blocking the use of the distal PAS.

**Figure 2:**
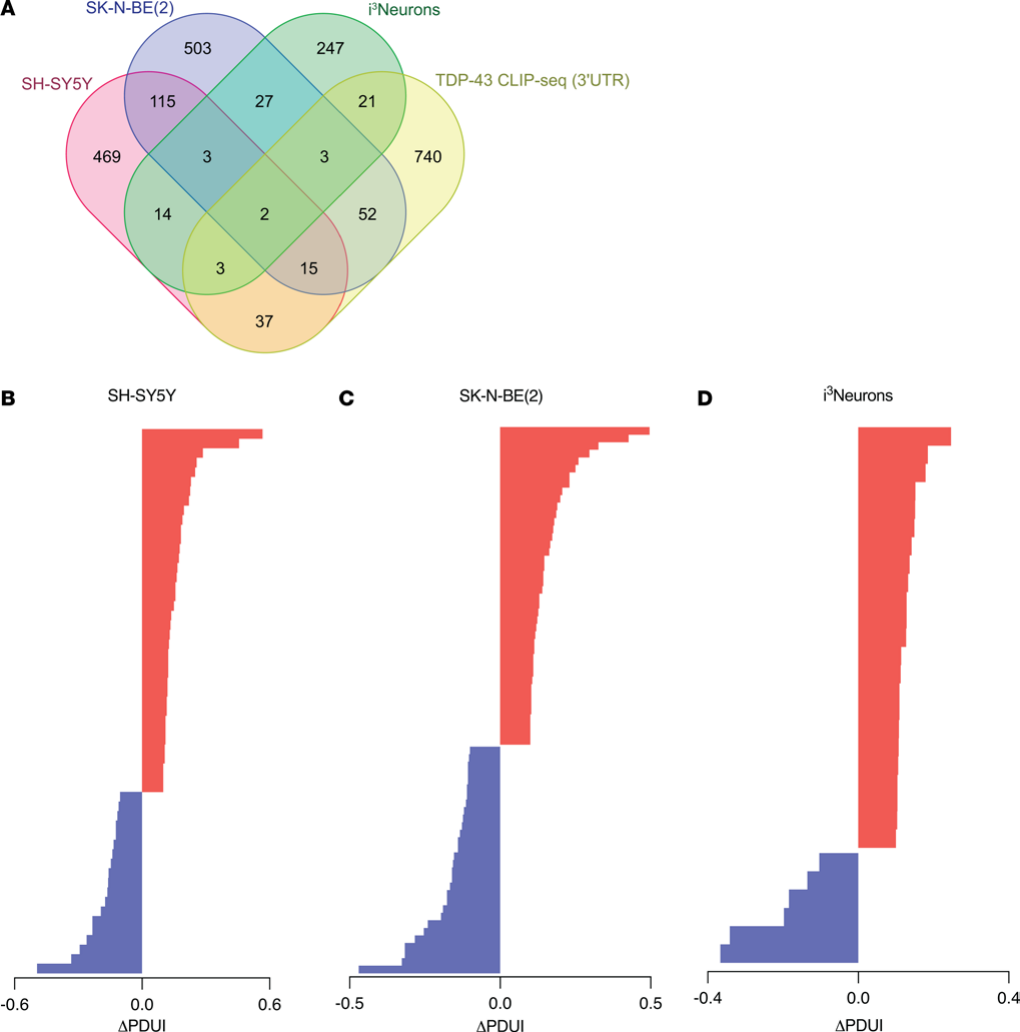
TDP-43 binds within the 3′ UTR of a subset of APA genes, preferentially blocking use of the distal PAS. (**A**) Venn diagram illustrating the proportion of APA genes for which there is published evidence of TDP-43 binding within the 3′ UTR. (**B**–**D**) Graphical representation of ΔPDUI for all APA genes with TDP-43–binding sites in the 3′ UTR.

By cross-referencing significant APA genes with TDP-43 enhanced crosslink and immunoprecipitation followed by sequencing (eCLIP-Seq) data from SH-SY5Y cells (26), we found that TDP-43 directly binds either within the 3′ UTR or in another region of the transcript to 43.2% (284/658) of APA genes in SH-SY5Y cells, 47.4% (341/720) of APA genes in SK-N-BE(2) cells, and 51.3% (164/320) of APA genes in i^3^Neurons (Figure 2A and Supplemental Figure 3). In neuronal cells chronically depleted of TDP-43, it is likely that cytotoxicity contributes to gene regulatory changes that do not necessarily reflect direct regulation by TDP-43. Indeed, we noted that in i^3^Neurons, only 36.7% of differentially expressed genes are targets of TDP-43 binding by eCLIP-Seq, while a higher proportion of CE (60.3%) and APA (51.3%) events are observed in genes directly bound by TDP-43 (Table 4). In support of our overall finding that TDP-43 depletion preferentially increases use of a distal PAS, we found that APA genes exhibit increased PDUI upon TDP-43 loss — when TDP-43 binds within the 3′ UTR, with i^3^Neurons displaying the largest trend (Figure 2, B–D).

**Figure 3:**
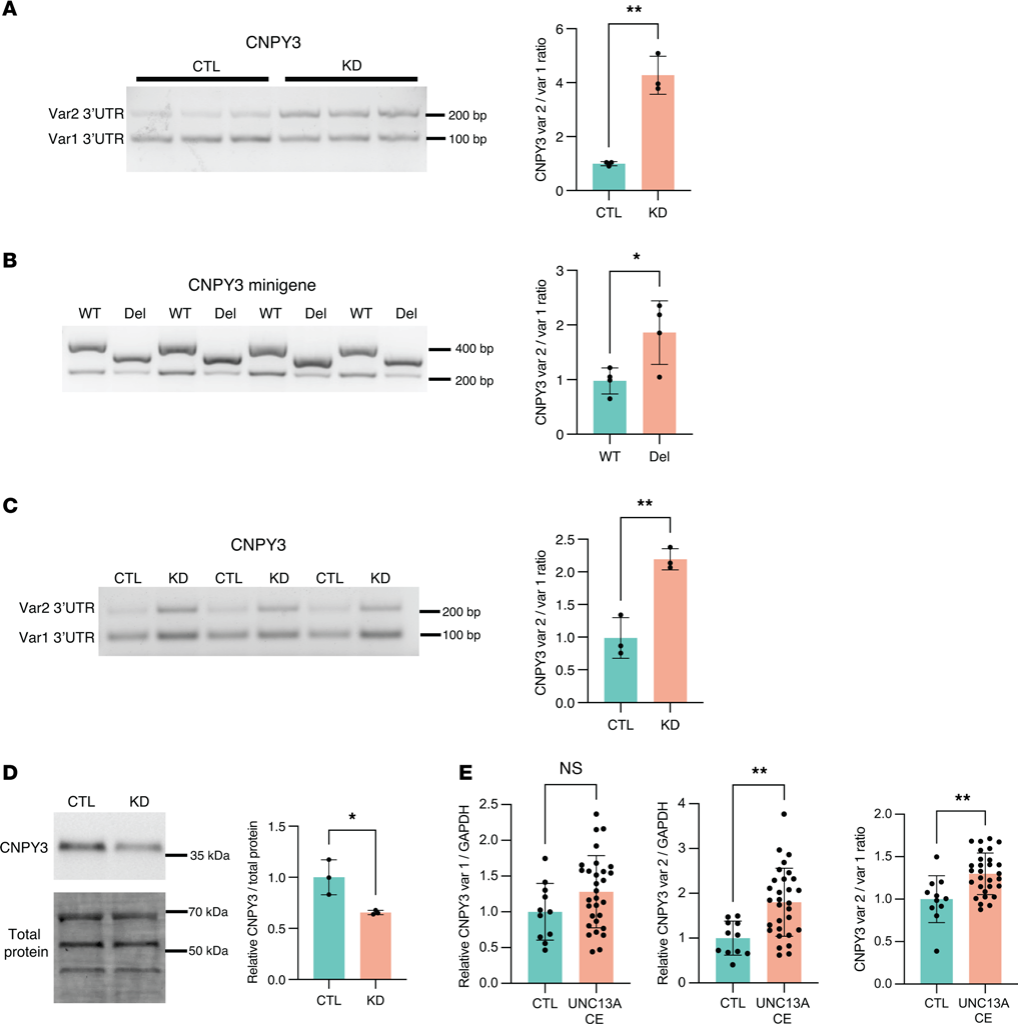
*CNPY3* APA increases the expression of an isoform variant with an alternative last exon in neuronal cells and in ALS/FTD and FTLD-TDP patient tissue. (**A**) RT-PCR analysis and qRT-PCR quantification of *CNPY3* APA isoforms with or without TDP-43 knockdown in SH-SY5Y cells. **P < 0.01; unpaired 2-tailed *t* test. *n* = 3 biological replicates. (**B**) RT-PCR analysis and qRT-PCR quantification of *CNPY3* APA isoforms in SH-SY5Y cells transfected with minigene constructs encoding exon 3, intron 3, and exon 4 of the *CNPY3* gene (NM_006586.5) with WT sequence or with the TDP-43–binding motif deleted (Del). *P < 0.05; unpaired 2-tailed *t* test. *n* = 4 biological replicates. (**C**) RT-PCR analysis and qRT-PCR quantification of *CNPY3* APA isoforms with or without TDP-43 knockdown in i^3^Neurons. **P < 0.01; unpaired 2-tailed *t* test. *n* = 3 biological replicates. (**D**) Immunoblot analysis of CNPY3 in i^3^Neurons reveals that *CNPY3* APA corresponds with a decrease in CNPY3 protein levels. *P < 0.05; unpaired 2-tailed *t* test. *n* = 3 biological replicates. (**E**) qRT-PCR quantification of CNPY3 APA isoforms in postmortem frontal cortex from healthy controls versus FTLD-TDP or ALS/FTD patients with confirmed CE inclusion in *UNC13A*. **P < 0.01; unpaired 2-tailed *t* test. *n* = 11 (control), *n* = 30 (*UNC13A* CE). All data are represented as mean values ± SEM.

To experimentally validate TDP-43 APA events, we first utilized the above SH-SY5Y cell model, in which a doxycycline-inducible shRNA against TDP-43 is stably integrated (12). We selected 2 APA genes to validate based on their robust and consistent shift in PDUI upon TDP-43 knockdown, and because TDP-43 binds directly within each 3′ UTR (Supplemental Figure 4). The Canopy FGF signaling regulator 3 (*CNPY3*) gene showed among the 3 greatest PDUI increases in each of our tested neuronal cell lines (Figure 2, B–D). *CNPY3* exhibits extensive alternative splicing, with numerous annotated transcript variants. In examining the RNA-Seq tracks for *CNPY3*, we found that TDP-43 knockdown significantly increases PAS usage within a specific transcript variant (NM_001318848.2, variant 2), defined by an alternative last exon without an upstream splice junction. In line with these data, we confirmed by quantitative reverse transcription PCR (qRT-PCR) that TDP-43 depletion results in a significant increase in the use of the variant two 3′ UTR relative to variant one (NM_006586.5) in SH-SY5Y cells (Figure 3A and Supplemental Figure 5A). To demonstrate that *CNPY3* APA is specifically regulated by TDP-43, we designed minigene expression constructs in which exon 3, intron 3, and exon 4 of the *CNPY3* gene (NM_006586.5) were cloned downstream of nanoluciferase with either WT sequence or with the TDP-43–binding site mutated. We found that deletion of the TDP-43–binding motif was sufficient to recapitulate the *CNPY3* isoform switch observed upon TDP-43 knockdown (Figure 3B). We further validated that *CNPY3* APA occurs in i^3^Neurons following TDP-43 depletion (Figure 3C and Supplemental Figure 6A). Interestingly, we observed a cell-type–specific effect of TDP-43 knockdown on *CNPY3* variant 1, whereby i^3^Neurons but not SH-SY5Y cells display a modest, but significant increase in *CNPY3* variant 1 expression in addition to a substantial increase in variant 2 expression (Supplemental Figure 5). Because we found an overall increase in both *CNPY3* isoforms in i^3^Neurons depleted of TDP-43, we evaluated CNPY3 protein levels. In contrast with increased expression of *CNPY3* at the RNA level, we found that CNPY3 protein expression is decreased upon TDP-43 knockdown (Figure 3D). This suggests that TDP-43 regulation of *CNPY3* APA may be functionally relevant in human neurons.

Given that CNPY3 was one of the most consistent APA events observed across cell models (Figure 2, B–D), we next investigated *CNPY3* APA in postmortem frontal cortex tissue from FTLD-TDP and ALS/FTD patients. Because even healthy controls exhibit low, but detectable levels of phosphorylated TDP-43 by ELISA, we used the presence or absence of the *UNC13A* CE as a more sensitive proxy for TDP-43 dysfunction (Table 5 and Supplemental Table 6). In agreement with our bioinformatics analyses and our experimental validation of *CNPY3* APA in cell models of TDP-43 depletion, we observed significant *CNPY3* APA, corresponding with increased expression of *CNPY3* isoform variant 2 in the frontal cortex of TDP-43 proteinopathy patients (Figure 3E). Altogether, these results demonstrate that TDP-43 regulation of poly(A) site selection occurs in the CNS of ALS/FTD and FTLD-TDP patients. Specifically, *CNPY3* APA increases the expression of a distinct protein-coding CNPY3 isoform with unknown function.

**Figure 4:**
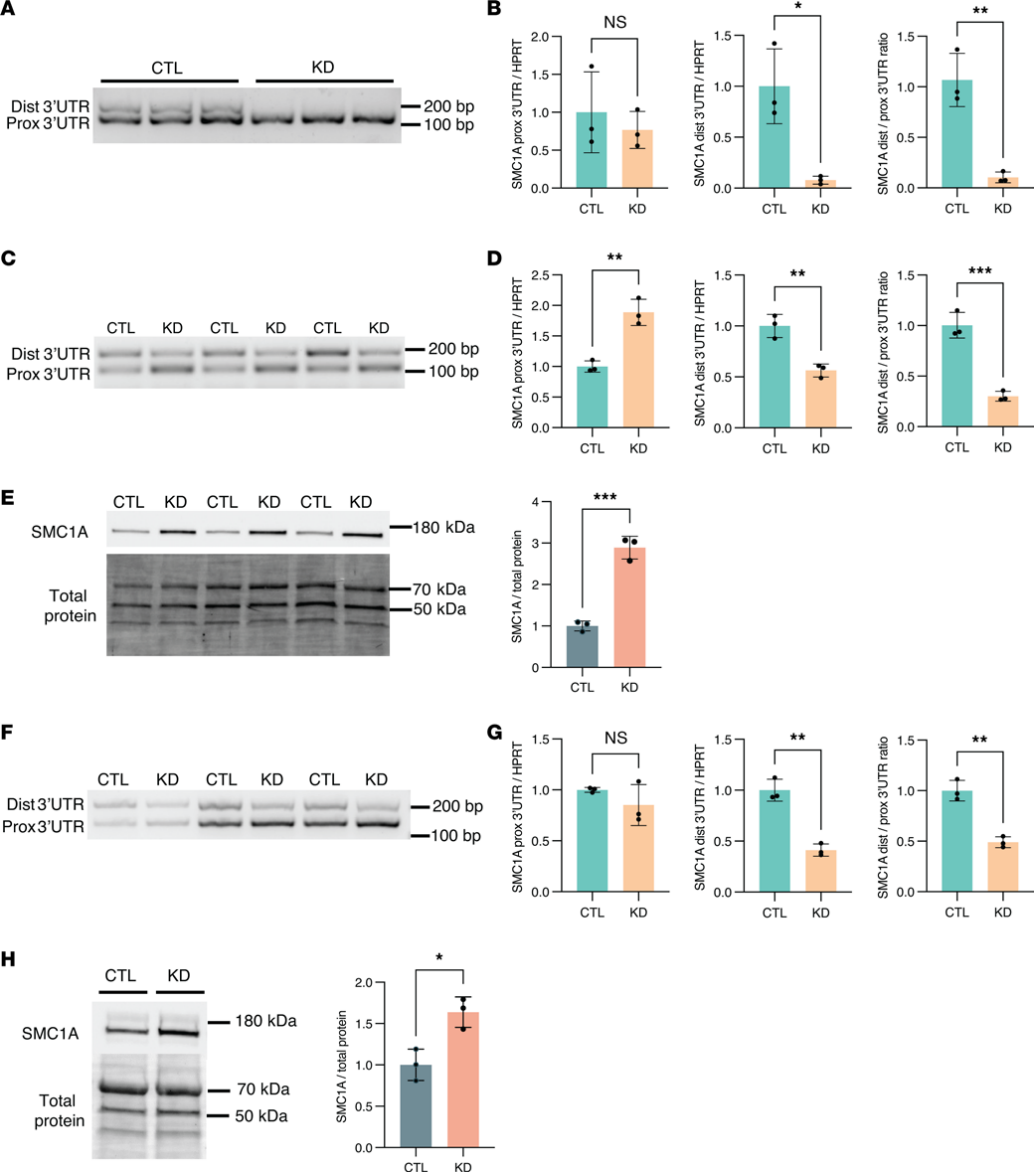
*SMC1A* APA corresponds with increased SMC1A protein levels in neuronal cells. (**A**, **C**, and **F**) RT-PCR analysis and (**B**, **D**, and **G**) qRT-PCR quantification of *SMC1A* APA in the presence or absence of TDP-43 knockdown in SH-SY5Y cells, i^3^Neurons, and iPSC-MNs, respectively. *P < 0.05; **P < 0.01; ***P < 0.001; unpaired 2-tailed *t* test. *n* = 3 biological replicates. (**E** and **H**) Immunoblot analysis of SMC1A reveals that *SMC1A* APA corresponds with an increase in SMC1A protein levels in i^3^Neurons and in iPSC-MNs, respectively. *P < 0.05; ***P < 0.001; unpaired 2-tailed *t* test. *n* = 3 biological replicates. All data are represented as mean values ± SEM.

In contrast to the majority of APA genes in which TDP-43 binds within the 3′ UTR, DaPars calculated a significant decrease in distal PAS usage for *SMC1A* (Supplemental Figure 4B), which we experimentally validated by qRT-PCR in SH-SY5Y cells (Figure 4, A and B). As noted, APA of *SMC1A* was previously characterized in HEK293 cells upon TDP-43 knockdown (14), indicating that this is a highly sensitive APA event across diverse cell types. Given that SMC1A is a subunit of the cohesin complex and plays a critical role in chromatin organization, we sought to investigate SMC1A in neuronal cells. TDP-43 depletion in i^3^Neurons resulted in significantly decreased expression of the long 3′ UTR *SMC1A* isoform and, in contrast to SH-SY5Y cells, increased expression of the short 3′ UTR *SMC1A* isoform (Figure 4, C and D). Furthermore, we found that *SMC1A* APA corresponded to a nearly 3-fold increase in SMC1A protein expression (Figure 4E). To determine whether TDP-43 regulation of *SMC1A* occurs in other neuronal cell types affected by TDP-43 proteinopathy, we similarly evaluated *SMC1A* APA and protein expression in DIV38 iPSC-derived motor neurons (iPSC-MNs) in which TDP-43 was knocked down for 10 days (Supplemental Figure 6B). We confirmed that TDP-43 loss indeed reduces use of the distal *SMC1A* PAS in human motor neurons (Figure 4, F and G). We further found that APA of *SMC1A* corresponds with a significant increase in SMC1A protein levels in iPSC-MNs (Figure 4H), suggesting that altered regulation of *SMC1A* APA may contribute to impaired chromatin organization upon TDP-43 loss, as previously reported in postmortem neuronal nuclei (27).

### Mutant TDP-43 induces APA in genes that function in the oxidative stress response.

**Figure 5:**
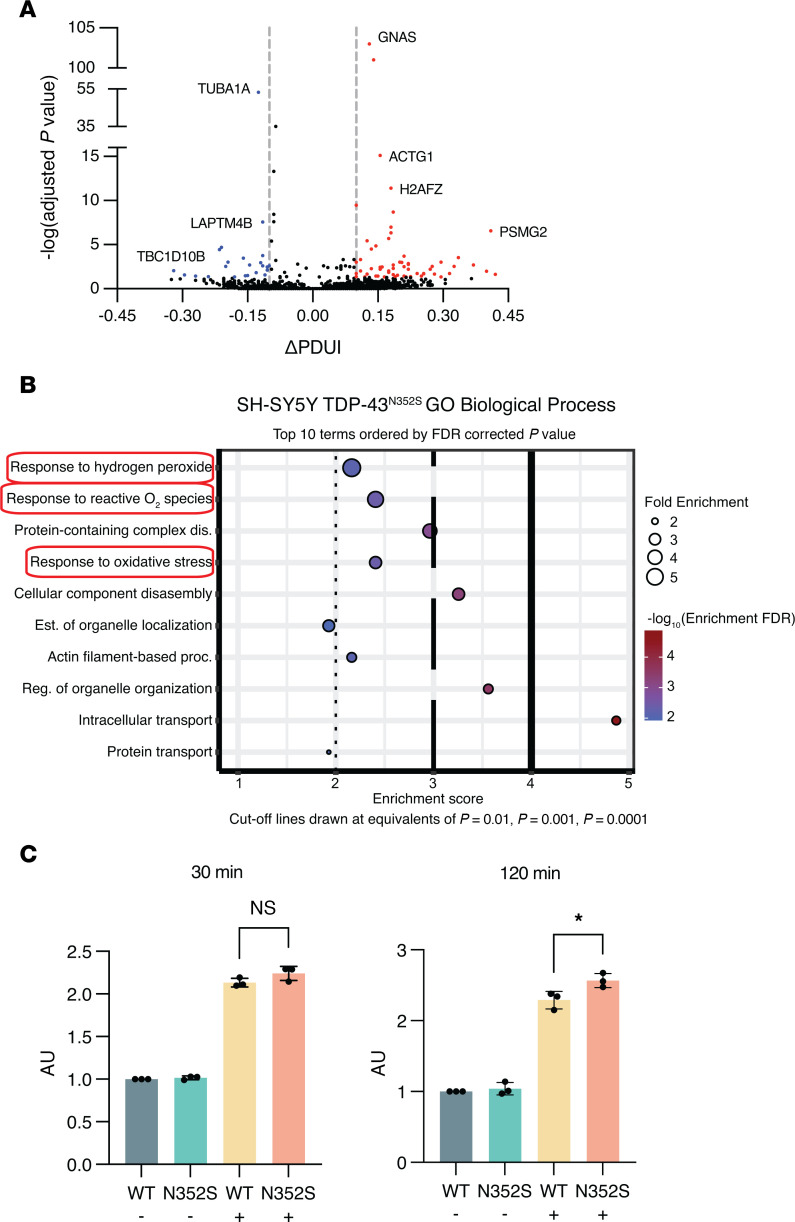
Mutant TDP-43 induces APA in genes functioning in oxidative stress response. (**A**) Volcano plot depicting APA genes in SH-SY5Y cells expressing homozygous TDP-43^N352S^ via CRISPR/Cas9 mediated genome editing. APA genes with FDR adj. *P* < 0.05 and ΔPDUI ≥ 0.1 are depicted in red, and APA genes with FDR adj. *P* < 0.05 and ΔPDUI ≤ –0.1 are depicted in blue. (**B**) GO biological processes analysis of APA events in TDP-43^N352S^ cells reveals impaired response to hydrogen peroxide (H_2_O_2_). (**C**) WT and TDP-43^N352S^ cells were treated with 100 μM H_2_O_2_. ROS was detected at 30 minutes and 120 minutes using a fluorometric intracellular ROS detection kit. *P < 0.05, unpaired 2-tailed *t* test. *n* = 3 biological replicates. All data are represented as mean values ± SEM.

To explore APA dysregulation that reflects TDP-43 mutation in addition to TDP-43 knockdown, we next applied DaPars to RNA-Seq data generated from SH-SY5Y cells with homozygous mutation of TDP-43^N352S^ achieved via CRISPR/Cas9 genome editing (11). Consistent with prior studies, 72% (59/82) of significant APA events corresponded with increased use of a distal PAS (Figure 5A, Supplemental Table 7). Notably, GO analysis revealed enrichment of genes that function in the “response to oxidative stress” pathway (Figure 5B). Numerous studies have implicated oxidative stress in ALS/FTD pathogenesis, including recent evidence that TDP-43 aggregation induces the generation of ROS (28). To determine whether TDP-43^N352S^ SH-SY5Y cells display an altered oxidative stress response, we measured ROS at 30 minutes and 120 minutes after treating WT or TDP-43^N352S^ SH-SY5Y cells with hydrogen peroxide. While there was no initial difference in ROS levels between control and TDP-43^N352S^ cells, ROS levels were significantly higher in TDP-43^N352S^ SH-SY5Y cells compared with WT cells after 120 minutes of exposure (Figure 5C). This provides proof of concept that TDP-43 APA genes function in cellular pathways implicated in neurodegenerative disease and that characterization of APA events can highlight disease-relevant phenotypes in cell models of ALS.

### Nuclear clearance of TDP-43 induces APA in ALS/FTD patient neurons.

**Figure 6:**
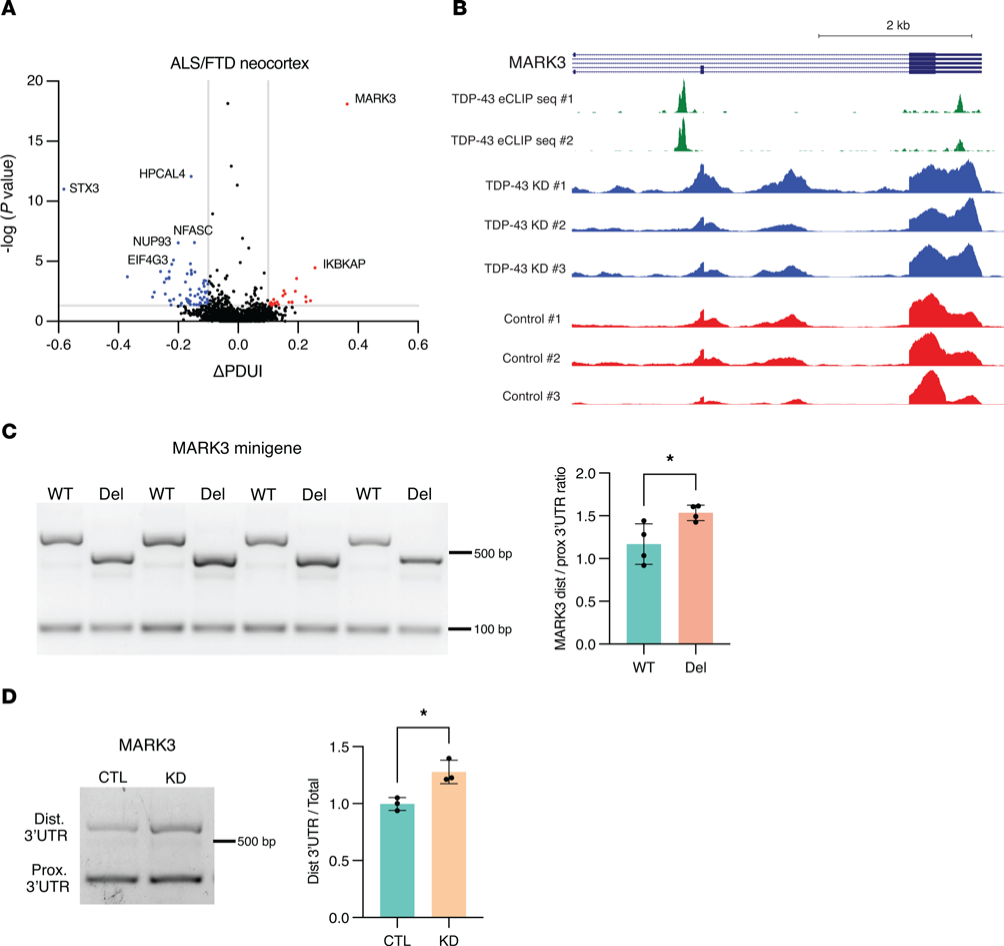
Nuclear clearance of TDP-43 induces APA in ALS/FTD patient neurons. (**A**) Volcano plot depicting APA genes in neuronal nuclei from 7 postmortem ALS/FTD neocortex samples sorted by FACS for the presence or absence of nuclear TDP-43. APA genes with *P* < 0.05 and ΔPDUI ≤ –0.1 are depicted in blue and APA genes with *P* < 0.05 and ΔPDUI ≥ 0.1 are in red. (**B**) eCLIP-Seq data showing the location of TDP-43 binding within the MARK3 3′ UTR, as well as in an upstream intronic region (green). MARK3 binding within the 3′ UTR is immediately upstream of the distal shift in 3′ UTR usage observed in TDP-43 negative neurons (blue versus red RNA-Seq tracks). (**C**) RT-PCR analysis and qRT-PCR quantification of *MARK3* APA in SH-SY5Y cells transfected with minigene constructs in which the *MARK3* 3′ UTR was cloned downstream of the NanoLuc luciferase gene with WT sequence or with the TDP-43 binding motif deleted (Del). *P < 0.05; unpaired 2-tailed *t* test. *n* = 4 biological replicates. (**D**) RT-PCR of distal 3′ UTR (top band) and proximal 3′ UTR (bottom band) of MARK3 in iPSC-MNs in which TDP-43 was knocked down with shRNA for 10 days. *P < 0.05; unpaired 2-tailed *t* test. *n* = 3 biological replicates. All data are represented as mean values ± SEM.

To further determine the significance of TDP-43 APA dysregulation in human patients, we considered APA events in neuron nuclei obtained from 7 postmortem ALS/FTD neocortex samples, where FACS sorting resulted in transcriptome data sets for neurons either containing or depleted of nuclear TDP-43 (27). We identified 87 APA genes (|ΔPDUI| > 0.1, *P* < 0.05) in neuronal nuclei lacking nuclear TDP-43 (Figure 6A, Table 6, and Supplemental Table 8), but unlike in neuronal cell culture models, we observed a preference toward negative ΔPDUI in postmortem TDP-43 nuclear-depleted neurons, as 72.7% (63/87) of genes exhibited 3′ UTR shortening. By correlating the most significant APA events (|ΔPDUI| > 0.1, FDR *P* < 0.05) with eCLIP-Seq of TDP-43, we noted that 57.7% of these APA genes are bound by TDP-43 (Table 7), suggesting that many APA events are directly regulated by TDP-43. We then performed GO analysis for APA events in coding mRNA with *P* < 0.05 and [|ΔPDUI| ≥ 0.1] and found enrichment of genes functioning in the “histamine response,” “synapse assembly,” and “protein transport” pathways (Supplemental Figure 7). These pathways have been previously implicated in ALS disease models, again underscoring potential contributions of APA events to ALS pathobiology (29–31).

The most substantial 3′ UTR lengthening in ALS/FTD neuronal nuclei (ΔPDUI = +0.363), was observed in the gene encoding *MARK3* (Table 6). Visualizing RNA-Seq tracks from this experiment with overlaid eCLIP-Seq generated by the ENCODE project (32), we confirmed that TDP-43 binds *MARK3* in its 3′ UTR immediately upstream of a canonical ATTAAA poly(A) signal (hg38: chr14:103503803-103503809) as well as in an upstream intron (Figure 6B). This suggests that nuclear TDP-43 normally represses use of a distal *MARK3* PAS, which then becomes preferentially utilized upon nuclear depletion of TDP-43. To test this hypothesis, we designed minigene expression constructs in which the *MARK3* 3′ UTR was cloned downstream of nanoluciferase with either WT sequence or with the TDP-43 binding site mutated. We found that deletion of the TDP-43–binding motif was sufficient to drive increased distal PAS usage in the *MARK3* 3′ UTR, supporting the hypothesis that TDP-43 represses use of the distal PAS (Figure 6C). Importantly, significant 3′ UTR lengthening in *MARK3* was also observed in our APA analysis in SH-SY5Y and SK-N-BE(2) cells (Figure 2, B and C), indicating that increased utilization of a distal poly(A) site in *MARK*3 is a prominent effect in neuronal cells upon loss of nuclear TDP-43. Given the consistency of this result, we evaluated this phenotype in iPSC-MNs and confirmed that TDP-43 knockdown can induce markedly increased use of a distal PAS in the *MARK3* gene (Figure 6D).

### MARK3 APA promotes increased tau S262 phosphorylation and altered subcellular distribution in neurons.

**Figure 7:**
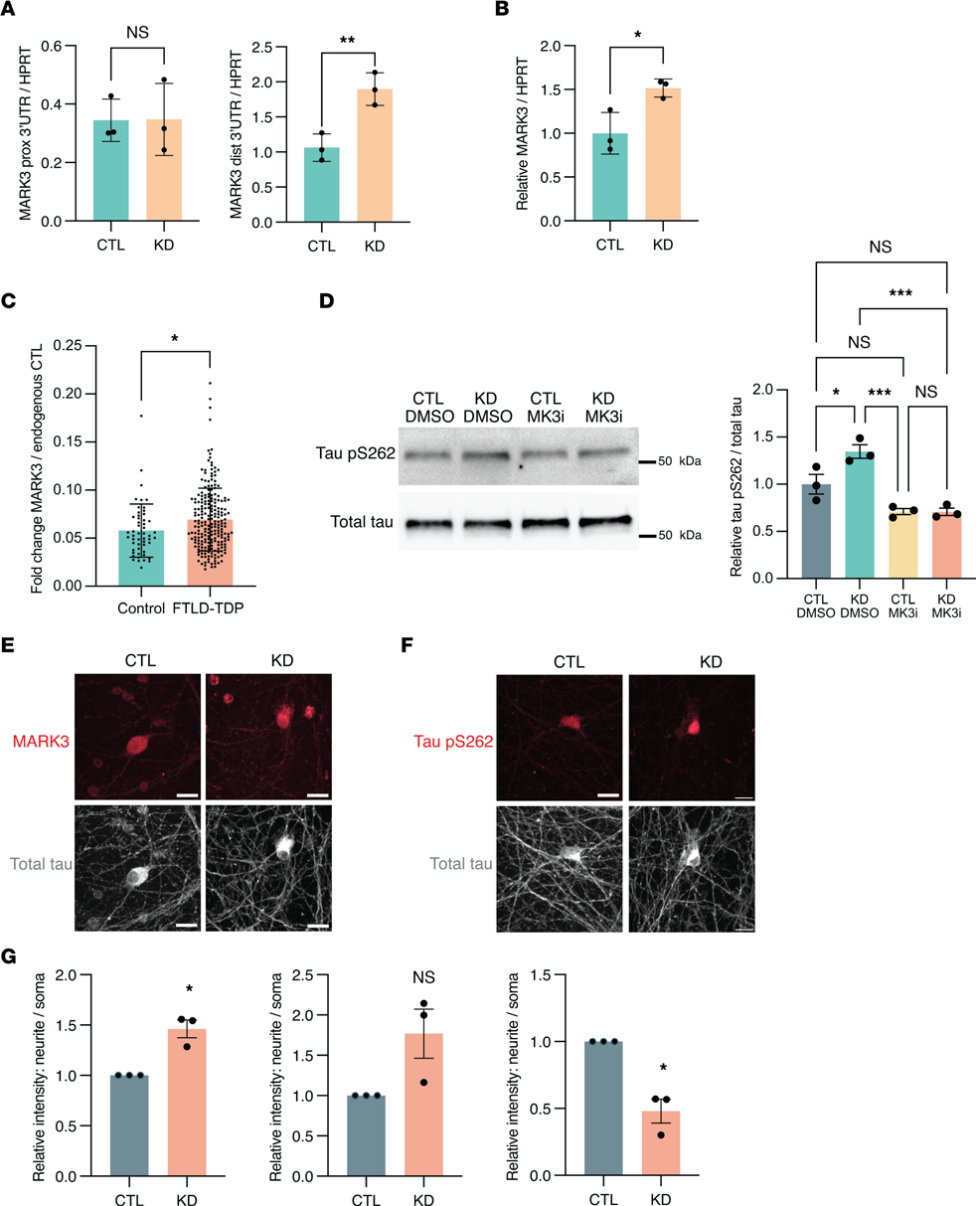
*MARK3* APA corresponds with increased *MARK3* transcript levels, increased tau S262 phosphorylation, and a change in MARK3 subcellular localization. (**A**) qRT-PCR quantification of *MARK3* APA or (**B**) total *MARK3* with or without TDP-43 knockdown in i^3^Neurons. *P < 0.05; **P < 0.01; unpaired 2-tailed *t* test. *n* = 3 biological replicates. (**C**) qRT-PCR quantification of total *MARK3* expression in control (*n* = 52) or FTLD (*n* = 221) postmortem frontal cortex tissue. *P < 0.05; Mann-Whitney *U* test. (**D**) Immunoblot analysis of S262 phosphorylated tau and total tau in i^3^Neurons transduced with lentivirus encoding control shRNA or shRNA TARDBP for 10 days and treated with DMSO (vehicle) or with 5 μM of PCC0208017 (MARK3/4 inhibitor) for the final 24 hours. *P < 0.05; ***P < 0.001; 1-way ANOVA. *n* = 3 biological replicates. (**E**) Representative images of i^3^Neurons immunostained for MARK3 and total tau in the presence or absence of TDP-43 knockdown. Scale bars:10 μm. (**F**) Representative images of i^3^Neurons immunostained for tau pS262 and total tau in the presence or absence of TDP-43 knockdown. Scale bars: 10 μm. (**G**) Quantification of fluorescence intensity. 15–20 images were analyzed per condition, *P < 0.05; 1-sample *t* test. *n* = 3 biological replicates. All data are represented as mean values ± SEM.

As MARK3 is a tau kinase associated with tau S262 phosphorylation in the early stages of AD pathogenesis (24), and because mutations in closely related MARK4 can significantly increase AD risk, promote hyperphosphorylation of tau, and induce neuron toxicity (33), we sought to investigate the functional consequences of *MARK3* APA in neurons. qRT-PCR analysis revealed that TDP-43 knockdown results in increased use of the distal *MARK3* PAS in i^3^Neurons (Figure 7A), which corresponds with an increase in overall *MARK3* expression (Figure 7B). To characterize the cis-regulatory elements within the *MARK3* 3′ UTR that may affect its metabolism, we evaluated predicted miRNA-binding sites using miRBD (34) and TargetScan (35), and we obtained predicted RBP-binding motifs using RBPmap (36) (Supplemental Figure 8). Both miRNA prediction tools highlighted a conserved binding motif for miR-142-3p within the *MARK3* 3′ UTR (Supplemental Table 9). Intriguingly, this miRNA was found to be upregulated in ALS mouse models and in sporadic ALS patients; indeed, serum levels of miR-142-3p have been negatively correlated with ALS clinical outcomes (37). We also identified 2 conserved RBP-binding motifs within the *MARK3* 3′ UTR, which are only present when poly(A) occurs at a distal *MARK3* PAS (Supplemental Figure 8 and Supplemental Table 10). The first motif is recognized by TDP-43 as well as by other RBPs (e.g., RBM24 and RBM38), while the second T/G-rich motif is predicted to be bound by several other RBPs, including TIA1 and HuR (Supplemental Figure 8B). The presence of highly conserved miRNA and RBP-binding motifs in the distal region of the *MARK3* 3′ UTR suggests that differential recognition of short versus long 3′ UTR isoforms by cis-regulatory elements may account for observed changes in *MARK3* mRNA expression upon TDP-43 knockdown.

Using a large cohort of frontal cortex tissue from healthy controls (*n* = 52) and FTLD-TDP patients (*n* = 221), we found that *MARK3* RNA expression is modestly increased in the frontal cortex in human disease (Figure 7C and Supplemental Table 11). Using a smaller, independent cohort of patient tissue (Table 5 and Supplemental Table 6), we validated our finding of increased *MARK3* RNA expression in the frontal cortex of ALS/FTD and FTLD-TDP patients relative to controls (Supplemental Figure 9, A and B). While we observed a small increase in the relative expression of the long 3′ UTR *MARK3* isoform in TDP-43 proteinopathy patient tissue, this did not reach statistical significance (Supplemental Figure 9C). Given that *MARK3* APA is prominent in monocultured neuronal cells (Figure 1, B and C) and in isolated neuronal nuclei depleted of TDP-43 (Figure 6A), but not in bulk frontal cortex tissue (Supplemental Figure 9C), it is likely that *MARK3* RNA isoform expression is regulated by distinct mechanisms in nonneuronal cells or in neurons without TDP-43 pathology in FTLD-TDP.

Based on our finding that TDP-43 depletion results in an overall increase in *MARK3* expression in i^3^Neurons and in postmortem patient tissue, we next evaluated tau S262 phosphorylation, a known target of MARK3 kinase activity. We found that TDP-43 knockdown significantly increases tau S262 phosphorylation in i^3^Neurons and that this effect is blocked by PCC0208017, a small molecule inhibitor of MARK3/4 (38) (Figure 7D). We similarly found that TDP-43 depletion in iPSC-MNs corresponds with a strong trend (*P* = 0.069) toward increased tau S262 phosphorylation (Supplemental Figure 10A). To determine whether MARK3 itself is sufficient to drive tau S262 hyperphosphorylation in human motor neurons, we transduced iPSC-MNs with lentivirus encoding a shRNA vector against *MARK3*, lentivirus encoding the *MARK3* gene, or control empty vector lentivirus. In iPSC-MNs subjected to MARK3 overexpression, we detected an approximately 2.4-fold increase in tau S262 phosphorylation (Supplemental Figure 10B). Moreover, we observed an approximately 50% reduction in tau S262 phosphorylation in iPSC-MNs subjected to MARK3 shRNA knockdown (Supplemental Figure 10B).

While these results demonstrate that MARK3 protein levels correlate with tau S262 phosphorylation in iPSC-MNs and that inhibition of MARK3/4 kinase activity blocks increased tau S262 phosphorylation in i^3^Neurons, we found no significant change in MARK3 protein expression in i^3^Neurons or iPSC-MNs upon TDP-43 knockdown (Supplemental Figure 10C and Supplemental Figure 11A). Similarly, MARK3 protein levels were unchanged in ALS/FTD and FTLD-TDP patient tissue relative to controls (Supplemental Figure 11B). We further confirmed that protein levels of the closely related tau S262 kinase MARK4 are unaffected by TDP-43 knockdown (Supplemental Figure 11C). Given that we observed increased tau S262 phosphorylation upon TDP-43 knockdown, which was blocked by treatment with a MARK3/4 inhibitor, yet we observed no increase in MARK3 protein levels, we next examined whether MARK3 subcellular localization is altered by TDP-43 depletion in i^3^Neurons. Indeed, we noted a significant increase in the subcellular localization of MARK3 to neurites relative to soma upon TDP-43 knockdown, which corresponded with a similar trend of increased neurite localization of tau pS262 (Figure 7, E–G). Moreover, we observed a decrease in total tau localization in the neurites of i^3^Neurons upon TDP-43 depletion (Figure 7, E–G), suggesting broader dysregulation of tau dynamics following TDP-43 knockdown. Taken together, these results reveal a potentially important mechanistic link between TDP-43 and tau biology and suggest that TDP-43 dysregulation of neuronal *MARK3* APA may contribute to altered cytoskeletal function in ALS/FTD and related neurodegenerative disorders.

## Discussion

The vast majority (>90%) of late-onset neurodegenerative diseases are sporadic, caused by ill-defined interactions between genetic and environmental risk factors. The complex etiology of these disorders has consequently hindered development of broadly effective therapies, and recent advances in the use of biological agents, which hold promise for treating rare familial forms of disease, may only benefit a small fraction of patients. This underscores the need to study convergent neurodegenerative disease mechanisms, such as the aberrant nuclear clearance and cytoplasmic aggregation of TDP-43, which has been implicated in a growing number of neurodegenerative disorders. Indeed, in addition to being a defining histopathological hallmark of ALS/FTD, emerging evidence suggests that TDP-43 dysfunction may play a pivotal role in dementia. Comorbid TDP-43 pathology is correlated with more severe cognitive impairment, more rapid disease progression, and increased brain atrophy in AD (6, 7). Furthermore, while TDP-43 and tau tangles rarely colocalize within the same inclusion, they can occur in close proximity within affected brain regions (39). Hence, delineating the cellular consequences of TDP-43 loss of function in neurons and other CNS cell types has the potential to be broadly relevant across multiple neurodegenerative diseases.

While the specific mechanisms linking TDP-43 dysfunction to neurodegeneration remain unclear, independent lines of investigation point toward aberrant RNA metabolism as the key driver of disease. Indeed, a number of independent studies have identified disease-relevant differentially expressed transcripts (e.g. *STMN2*) and alternatively spliced transcripts (e.g., *UNC13A*) that are being regulated by TDP-43 (9, 11–13). TDP-43 is also known to regulate APA, and previous work in HEK293 cells defined a position-dependent principle by which binding of TDP-43 within 75 nucleotides of a PAS represses its usage, while binding further downstream of a PAS site enhances its usage (14). Here we found that TDP-43 loss of nuclear function changes poly(A) site selection in hundreds of transcripts in neuron cell culture models of TDP-43 depletion or mutation and in postmortem ALS/FTD patient neuron nuclei lacking TDP-43. Based on TDP-43 eCLIP-Seq data from SH-SY5Y cells, we found evidence that approximately 40%–60% of APA transcripts directly interact with TDP-43, although we note that additional TDP-43 CLIP-Seq datasets will be necessary to ascertain which APA transcripts are bound by TDP-43 in distinct cell types. While the majority of TDP-43–regulated APA events were cell-type specific, a number of highly reproducible changes in PAS usage were identified across multiple datasets, including significantly increased distal PAS usage in *CNPY3*, significantly reduced distal PAS usage in *SMC1A*, and significantly increased distal PAS usage in *MARK3*. We further showed that *CNPY3* APA occurs in the frontal cortex of ALS/FTD and FTLD-TDP patients, indicating that TDP-43 regulation of APA is indeed a feature of human disease. Notably, *CNPY3* was not detected in our bioinformatics analysis of postmortem ALS/FTD neuronal nuclei; thus, additional APA events detected in cell models of TDP-43 depletion may have functional relevance in TDP-43 proteinopathies. Two independent reports have also evaluated the effect of TDP-43 depletion in postmortem ALS/FTD patient neuron nuclei using an identical transcriptome data set (27), and despite employing distinct APA detection algorithms and bioinformatics analysis pipelines, both investigations identified hundreds of TDP-43 target genes subject to APA (40, 41), underscoring the relevance of TDP-43 dysregulation of poly(A) site selection to the disease process in TDP-43 proteinopathy.

To assess the functional relevance of TDP-43 APA in ALS/FTD disease pathogenesis, we also performed GO analysis on the APA hits and identified various pathways, including response to oxidative stress, which we validated as impaired in TDP-43^N352S^ SH-SY5Y cells by documenting elevated ROS upon treatment with hydrogen peroxide. To examine the functional implications of TDP-43 APA, we pursued directed studies on the tau kinase MARK3, our top hit from the DaPars analysis of ALS/FTD patient postmortem frontal cortex neuron nuclei containing or depleted of TDP-43. Inclusions of tau protein accumulate in the brains of patients affected with familial and sporadic FTD (42), and tau is centrally involved in AD where as many as 57% of affected patients display TDP-43 nuclear clearance and cytoplasmic aggregation in addition to the hallmark neurofibrillary tangles of tau protein (6). To determine whether TDP-43 APA of MARK3 could contribute to disease pathogenesis in ALS/FTD and AD, we evaluated the effect of TDP-43 knockdown on MARK3 phosphorylation of tau at S262, a well-established MARK3 phosphorylation site with potential pathophysiological implications, because tau phosphorylation at this residue, which is located within a tau microtubule-binding site (43), can reduce the microtubule-stabilizing properties of tau and antagonize microtubule tracking at the plus end by disrupting interaction between end-binding protein 1 and tubulin (44, 45). We found that TDP-43 knockdown in i^3^Neurons resulted in significantly (*P* = 0.0283) increased tau S262 phosphorylation and yielded increased expression of a *MARK3* transcript with a lengthened 3′ UTR accompanied by an overall increase in *MARK3* RNA levels upon TDP-43 knockdown in neuronal cells. *MARK3* RNA expression was also significantly (*P* = 0.0124) elevated in the frontal cortex of 2 cohorts of ALS/FTD and FTLD-TDP patients; however, we found that MARK3 protein levels were unchanged in i^3^Neurons or iPSC-MNs upon TDP-43 knockdown and that MARK3 protein levels did not differ significantly between ALS/FTD and FTLD-TDP patients and unaffected controls in the frontal cortex. As the 3′ UTR can regulate subcellular transcript localization and the site of local protein translation (46), we also examined the effect of TDP-43–mediated lengthening of the *MARK3* 3′ UTR on its subcellular localization, and we noted that TDP-43 knockdown resulted in significantly (*P* = 0.0348) greater localization of MARK3 protein to neurites in comparison with cell soma and we detected increased levels of phosphorylated S262 tau in neurites versus soma. Hence, it is possible that TDP-43 APA of *MARK3* promotes increased tau S262 phosphorylation by shifting the subcellular localization of MARK3 protein, indicating that TDP-43 APA of *MARK3* may contribute to tau dysregulation and altered cytoskeletal dynamics in TDP-43 proteinopathies, even in the absence of overt tau pathology. The effect of *MARK3* APA and increased tau S262 phosphorylation on microtubule dynamics in TDP-43 proteinopathies should be the focus of future studies. In a related study, TDP-43 APA of the FTLD-TDP risk gene *TMEM106B* was documented in postmortem frontal cortex samples from FTLD-TDP patients and proposed to alter *TMEM106B* translational efficiency, potentially destabilizing TMEM106B dimer formation (41).

Nearly 2 decades ago, the discovery of TDP-43 aggregates in the brains of patients with ALS and FTD revolutionized the neurodegenerative disease field (47) and set the stage for intense research into the role of TDP-43 dysfunction in ALS/FTD and related disorders. Over the last decade, considerable evidence has accumulated indicating that TDP-43 dysregulation of RNA splicing is likely a key step in the disease process for TDP-43 proteinopathies. However, in addition to regulating splicing, TDP-43 performs another fundamentally important function in RNA processing — selection of the site of poly(A). It is important to emphasize that poly(A) site selection is a critical factor in regulating gene function. Within the 3′ UTR are binding sites for miRNAs and RBPs, and these interactions dictate transcript stability and can modulate expression at the protein level. Furthermore, cis-acting regulatory elements controlling mRNA subcellular localization exist within 3′ UTRs (46). Subcellular localization of RNAs within neurons and other CNS cell types could profoundly affect cellular function; hence, altered subcellular localization due to APA could be contributing to neurodegeneration, as reported for *ELK1* in an independent study (40). Here, we report that TDP-43 APA can alter the subcellular localization of MARK3 with consequences for tau function, underscoring the disease relevance of TDP-43 dysregulation of poly(A) site selection. Our results reveal that TDP-43 dysregulation of poly(A) site selection could be driving the pathogenesis of various neurodegenerative diseases, likely in combination with altered splicing and CE inclusion. Defining genes subject to TDP-43 APA should thus be a major focus of future studies, as a number of these APA genes could be targets for developing biomarkers and engineering novel therapies. As antisense oligonucleotides can regulate poly(A) site usage by steric hindrance (48, 49), delineation of genes subject to APA may yield candidates for a next generation of biological agents for treatment of TDP-43 proteinopathy.

## Methods

### Sex as a biological variable.

This study includes DaPars analysis of 1 published transcriptome data set (27), based on sorting of neuron nuclei containing or depleted of TDP-43 from 7 postmortem ALS/FTD patients (3 females and 4 males), where the study was not powered to evaluate sex as a biological variable. For experiments using postmortem human tissue, roughly equal numbers of males and females were analyzed (Supplemental Table 6 and Supplemental Table 11), and sex was not evaluated as a biological variable.

DaPars software, described previously (25), allows for the joint analyses of multiple samples based on a 2-normal mixture model, which is used to calculate the PDUI value. Briefly, we extract a 3′ UTR annotation for each gene using the “DaPars_Extract_Anno.py” script within DaPars2. We then used the “samtools flagstat” command (50) to calculate the sequencing depth for each sample. Finally, we use DaPars2 to calculate the percentage of the PDUI divided by the total expression level of each transcript across samples.

### Cell culture.

WT human neuroblastoma SH-SY5Y cells (ATCC) and SH-SY5Y cells with homozygous mutation of TDP-43^N352S^ (obtained from the lab of Don Cleveland; ref. 11) were maintained in 50% Eagle’s minimum essential medium (EMEM) (ATCC, 30-2003), 50% Ham’s F12 (ThermoFisher, 11765047), supplemented with 10% fetal bovine serum, and 50 U/mL penicillin-streptomycin. Cells were grown at 37°C and 5% CO_2_.

### Doxycycline-inducible knockdown of TDP-43.

SH-SY5Y cells with stable integration of a doxycycline-inducible shRNA cassette targeting TDP-43 were obtained from the lab of Pietro Fratta (12). Cells were treated with 1 μg/mL doxycycline (Sigma-Aldrich, D9891-1G) for 7 days to induce TDP-43 knockdown.

### Minigene expression constructs.

The *MARK3* minigene construct comprises an insert containing the NanoLuc luciferase gene (FLAG-tagged at the C-terminus) and the *MARK3* 3′ UTR cloned into the pJTI R4 DEST CMV pA vector (ThermoFisher) using BbsI and PmeI restriction enzyme sites. The HSV TK poly(A) signal was deleted. The *CNPY3* minigene construct comprises an insert containing the NanoLuc luciferase gene immediately followed by exon 3, intron 3, and exon 4 of the *CNPY3* gene cloned into the pJTI R4 DEST CMV pA vector (ThermoFisher) using BbsI and PmeI restriction enzyme sites. Exon 4 is FLAG tagged at the C-terminus. Synthesis of the described inserts and assembly of the vectors was performed by Azenta. With the exception of the primers used to amplify the *MARK3* long 3′ UTR, all primer pairs were designed to specifically amplify minigene construct products by containing either FLAG tag or NanoLuc luciferase gene sequence.

qRT-PCR primer sequences were as follows: CNPY3 minigene var1 3′ UTR fwd: GCATGTCAGAGACCTTTGAGAC; CNPY3 minigene var1 3′ UTR rev: GTCATCGTCTTTGTAGTCCTGCTTC; CNPY3 minigene var2 3′ UTR fwd: GAATCCTCGCCTCGGACTTG; CNPY3 minigene var2 3′ UTR rev: GGATGAAGGACAATCCCGAATC; MARK3 minigene fwd: GACGATGACGATAAATAACCCAGTGA; short 3′ UTR MARK3 rev: TAGAAGATGCAGACGTTATTGCC; long 3′ UTR MARK3 fwd: CAGGTTTACAGTTCATGCCTGT; long 3′ UTR MARK3 rev: CACACACAAGCAATGTTCACAAC.

### i^3^Neurons.

i^3^Neurons were generated from the KOLF2.1J iPSC line (51) in which neurogenin 2 (NGN2), under the control of a tetracycline-inducible promoter, was stably integrated into the adeno-associated virus integration site (AAVS) locus. iPSCs were cultured in mTeSR (STEMCELL Technologies, 100-0276). As previously described (52), NGN2 expression was induced by doxycycline for 3 days; then immature i^3^Neurons were replated and cultured for an additional 7 days (in the presence of doxycycline) prior to experimentation.

### iPSC-MNs.

iPSCs from the KOLF2.1J line were maintained in mTeSR (STEMCELL Technologies, 100-0276). According to the manufacturer’s instructions, iPSCs were differentiated into motor neurons from days 0–14 (STEMCELL Technologies, 100-0871) and subsequently matured from days 14–28 (STEMCELL Technologies, 100-0872). iPSC-MNs were transduced using lentiviral vectors at day 28 and collected at day 38. Cells were cultured at 37°C and 5% CO_2_.

### Lentiviral vector transduction.

Lentiviral constructs encoding GFP (VectorBuilder, LVM[VB010000-9298rtf]-C), MARK3-V5 (Addgene, 107235), shRNA control (Sigma, SHC002), tardbp shRNA (Sigma, TRCN0000016038), or MARK3 shRNA (Sigma, TRCN0000001564) were packaged into lentivirus by VectorBuilder. iPSC-MNs were transduced with 5 MOI lentivirus at day in vitro (DIV) 28 for 10 days, and i^3^Neurons were transduced with 5 MOI lentivirus at DIV 10 for 10 days.

shRNA target sequences were as follows: control: CAACAAGATGAAGAGCACCAA; Tardbp: GCTCTAATTCTGGTGCAGCAA; and MARK3: TGTGTGTGAAGTGGTGTATAT.

### MARK3/4 inhibitor treatment.

i^3^Neurons were treated with 5 μM MARK3/4 inhibitor (PCC0208017[38]) or DMSO (vehicle) for 24 hours prior to harvesting cells for downstream assays.

### RT-PCR analysis.

RNA was isolated from cell lysates (QIAGEN, 74106) and reverse transcribed to generate cDNA (Thermo Fisher, 11756500). PCR was performed using 10–30 ng cDNA template, 0.5 μM forward and reverse primers, and One*Taq* DNA polymerase mastermix (NEB, M0488L). PCR products were separated by agarose gel electrophoresis using 1.5% agarose (VWR, 0710) in 1× TBE buffer at 150V.

RT-PCR primer sequences were as follows: MARK3 fwd: GGTTTAAGCGGATATCGGGG; MARK3 prox rev: TAGAAGATGCAGACGTTATTGCC; and MARK3 dist rev: CACACACAAGCAATGTTCACAAC.

### qRT-PCR.

Relative fold change of CNPY3, SMC1A, and MARK3 was determined by qRT-PCR using SYBR Green Master Mix (Thermo Fisher, A25776) with hypoxanthine-guanine phosphoribosyltransferase (HPRT) as an endogenous control.

qRT-PCR primer sequences were as follows: CNPY3 var1 3′ UTR fwd: CCAGCATCCTCTGTCCTGA; CNPY3 var1 3′ UTR rev: GAAGAGGGCACAGCCAAG; CNPY3 var2 3′ UTR fwd: GACCACCTGGGATCTTCCT; CNPY3 var2 3′ UTR rev: CACATCGTGGATCTTGCTGAG; SMC1A prox 3′ UTR fwd: CCTGTCTGGATCCCTAAGCTG; SMC1A prox 3′ UTR rev: CTCCAGACCTAACATCACCTCTG’ SMC1A dist 3′ UTR fwd: GTTAGTCAGTAGCAGTAGGAGGAG; SMC1A dist 3′ UTR rev: GCATTCACAGGGAAATAAGGAAGAC; total MARK3 fwd: CAGTCTCCTCACCACAAAGTGC; total MARK3 rev: TGCTGGTCTGACTCCTTTTCGG; short 3′ UTR MARK3 fwd: GGTTTAAGCGGATATCGGGG; short 3′ UTR MARK3 rev: TAGAAGATGCAGACGTTATTGCC; long 3′ UTR MARK3 fwd: CAGGTTTACAGTTCATGCCTGT; long 3′ UTR MARK3 rev: CACACACAAGCAATGTTCACAAC; HPRT fwd: CATTATGCTGAGGATTTGGAAAGG; and HPRT rev: CTTGAGCACACAGAGGGCTAC.

### Evaluation of CNPY3 and MARK3 APA in ALS/FTD and FTLD-TDP frontal cortex.

To evaluate total *MARK3* RNA expression, our study cohort included 273 postmortem cases classified into 2 groups: healthy controls (*n* = 52) and FTD with TDP-43 pathology (FTLD-TDP) (*n* = 221). A separate cohort, in which phosphorylated TDP-43 (pTDP-43) was previously quantified by ELISA and the presence or absence of the *UNC13A* CE was previously determined, was used to evaluate *MARK3* and *CNPY3* APA. This cohort (Table 5) consisted of healthy controls with low, but detectable levels of pTDP-43 and no detectable *UNC13A* CE (*n* = 11) and ALS/FTD or FTLD-TDP cases with low or high levels of pTDP-43 and confirmed *UNC13A* CE expression (*n* = 30). Finally, to evaluate MARK3 protein levels, our study cohort consisted of healthy controls with low pTDP-43 (*n* = 5) and ALS/FTD or FTLD-TDP cases with high pTDP-43 (*n* = 11). A summary of patient data is included in Supplemental Table 6 and Supplemental Table 11. RNA was extracted from frontal cortex tissue following the manufacturer’s protocol using the RNAeasy Plus Mini Kit (QIAGEN) and as previously described (13, 53). Up to 3 cuts of the sample was used for extraction and only the high-quality RNA samples were processed for downstream analysis. RNA concentration was measured by using Nanodrop technologies (Thermo Fisher) and the RNA integrity number (RIN) was evaluated by Agilent 2100 Bioanalyzer (Agilent Technologies). Subsequently, 500 ng of the total RNA extracted was reverse transcribed into cDNA using the High-Capacity cDNA Transcription Kit (Applied Biosystems) per the manufacturer’s instructions. cDNA samples, in triplicate, with SYBR GreenER qPCR SuperMix (Invitrogen), were further evaluated for the quantitative real-time PCR (qRT-PCR) on a QuantStudio 7 Flex Real-Time PCR System (Applied Biosystems). Relative quantification of total *MARK3* levels was determined using the ΔΔCt method and normalized to 2 endogenous controls, *GAPDH* and *RPLP0*, while quantification of *MARK3* and *CNPY3* APA was normalized to *GAPDH*. Protein was extracted from frontal cortex tissue using RIPA lysis buffer, and MARK3 (Cell Signaling Technology, 9311S, 1:2000) was normalized to GAPDH (Meridian Life Science, H86504M, 1:10,000).

### Immunoblot analysis.

Protein extracts were prepared in RIPA lysis buffer (Thermo Fisher, 89900) with protease and phosphatase inhibitors (Thermo Fisher, 87786). Lysates were sonicated at 4 °C (Bioruptor Pico), denatured in LDS sample buffer (Thermo Fisher, NP0007) with a reducing agent (Thermo Fisher, NP0009), and heated at 70 °C for 10  minutes. Lysates were electrophoresed by sodium dodecyl sulfate–polyacrylamide gel electrophoresis using a 4%–20% gradient polyacrylamide gel (Bio-Rad, 5678093) and transferred to a 0.45 μm polyvinylidene difluoride membrane (Bio-Rad, 1704157). Blots were incubated with primary antibodies diluted in 5% nonfat milk in TBST (TRIS-buffered saline, 0.05% Tween-20) overnight at 4 °C and with secondary antibodies diluted in 5% nonfat milk in TBST (Thermo Fisher, A16078 [goat anti-mouse HRP, 1:3000], A16110 [goat anti-rabbit HRP, 1:3000], A32728 [goat anti-mouse Alexa Fluor 647, 1:1000], A32733 [goat anti-rabbit Alexa Fluor 647, 1:1000]) for 60  minutes at RT. Detection was performed using enhanced chemiluminescence substrate (Genesee Scientific, 20–300S) and blots were imaged on a ChemiDoc MP (Bio-Rad). Primary antibodies were as follows: TDP-43 (Proteintech, 12892-1-AP, 1:3000), MARK3 (Cell Signaling Technology, 9311S, 1:2000), MARK4 (Proteintech, 20174-1-AP, 1:2000), CNPY3 (Proteintech, 15215-1-AP, 1:1000), SMC1A (abcam, ab9262, 1:2500), phospho-tau Ser262 (Thermo Fisher, 44-750-G, 1:2000), and TAU-5 (ThermoFisher, AHB0042, 1:2000).

### Immunofluorescence staining and image analysis.

i^3^Neurons plated in 8-well chamber slides (Thermo Fisher, 154941PK) were fixed in 4% PFA for 15 minutes, permeabilized, and blocked in 0.3% Triton X-100 in 2% normal donkey serum (NDS) for 30 minutes, and incubated with primary antibodies in 1.5% NDS for overnight at 4°C. Secondary antibodies were diluted in 1.5% NDS and incubated with samples for 60 minutes at room temperature. Hoescht was added to visualize nuclei (Thermo Fisher, H1399). Slides were mounted using ProLong Glass Antifade Mounting Media (Thermo Fisher, P36984). Cells were imaged on a Nikon A1R confocal microscope using a ×60 objective. Antibodies used included the following: MARK3 (Cell Signaling Technology, 9311S, 1:200). phospho-tau Ser262 (Thermo Fisher, 44-750-G, 1:200), and TAU-5 (Thermo Fisher, AHB0042, 1:500). Fluorescence intensity of tau pS62, total tau, or MARK3 was quantified as described (54). Each raw image was maximally projected and fluorescence intensity thresholds were set using identical values for all conditions per experiment. Thresholded areas occupied by a fluorescence signal for tau pS62, total tau, or MARK3 were measured; then fluorescence area was divided by the cell area, which was determined by subtracting the background fluorescence. To quantify the neurite/soma expression levels of these proteins, the neurite values were defined by the area 10 μm away from the soma. All quantitation is represented as combined results for all the repeats of a particular experiment (*n* = 10–15 images analyzed per condition).

### Detection of ROS.

WT and TDP-43^N352S^ SH-SY5Y cells were plated on a 96-well plate and treated for 120 minutes with 100 μM H_2_O_2_. A fluorometric intracellular ROS detection kit was used to detect ROS levels at 30 and 120 minutes (Sigma, MAK144) using a Varioskan microplate reader (Thermo Fisher).

### GO analysis.

GO analysis was performed using ShinyGO, version 0.77 (55), for GO Biological Processes and GO Molecular Function pathway databases. GO analysis was performed for APA events in protein-coding genes with ΔPDUI 0.1 and *P* < 0.05. Graphical representations of the top 10 pathways with an FDR adj. *P* < 0.05 cut-off were generated in R, version 4.3.1, utilizing the ggplot and sjPlot packages.

### Statistics.

As indicated, for pairwise comparisons, statistical significance was determined by unpaired 2-tailed Student’s *t* test or by Mann-Whitney *U* test (Figure 7C). For pairwise comparisons in which the controls of distinct biological replicates were normalized to 1, statistical significance was determined by a 2-tailed, 1-sample *t* test in which experimental conditions were compared with a value of 1. For multiple comparisons, statistical significance was determined by 1-way ANOVA with post hoc Tukey’s test. Hypergeometric distribution was used to determine the statistical significance of Venn diagram overlaps. The significance level (α) was set at 0.05 for all experiments. Data were compiled and analyzed using Microsoft Excel or Prism 10 (GraphPad).

### Study approval.

This study does not include research conducted on animals or human subjects. For more information on deidentified postmortem human tissue used in this study, see Supplemental Table 6 and Supplemental Table 11.

### Data availability.

Full documentation and workflow of the DaPars2 algorithm is publicly available on github: https://github.com/3UTR/DaPars2/tree/v2.1 Values for all data points in graphs are reported in the Supporting Data Values file. Deidentified information on postmortem human samples is available in Supplemental Table 6 and Supplemental Table 11. Any additional information on underlying data can be made available by the corresponding author upon request.

## Author contributions

FJA, YC, S Michels, and ARLS provided the conceptual framework for the study. FJA, YC, S Michels, OHT, MGH, DWD, MP, LP, WL, and ARLS designed the experiments. FJA, YC, S Michels, MRC, CMS, WY, VMJ, KJW, JP, RG, YG, S Menon, WGS, SLC, AZ, KCKE, and SH performed the experiments. FJA, YC, S Michels, OHT, MGH, WL, and ARLS analyzed the data. FJA, YC, S Michels, and ARLS wrote the manuscript. The study was initiated by FJA, who was joined by YC, and then by S Michels in leading the project, who are listed as equally contributing co–first authors in this order.

## Supplementary Material

Supplemental data

Unedited blot and gel images

Supplemental table 1

Supplemental table 2

Supplemental table 3

Supplemental table 4

Supplemental table 5

Supplemental table 6

Supplemental table 7

Supplemental table 8

Supplemental table 9

Supplemental table 10

Supplemental table 11

Supporting data values

## Figures and Tables

**Table 3:**
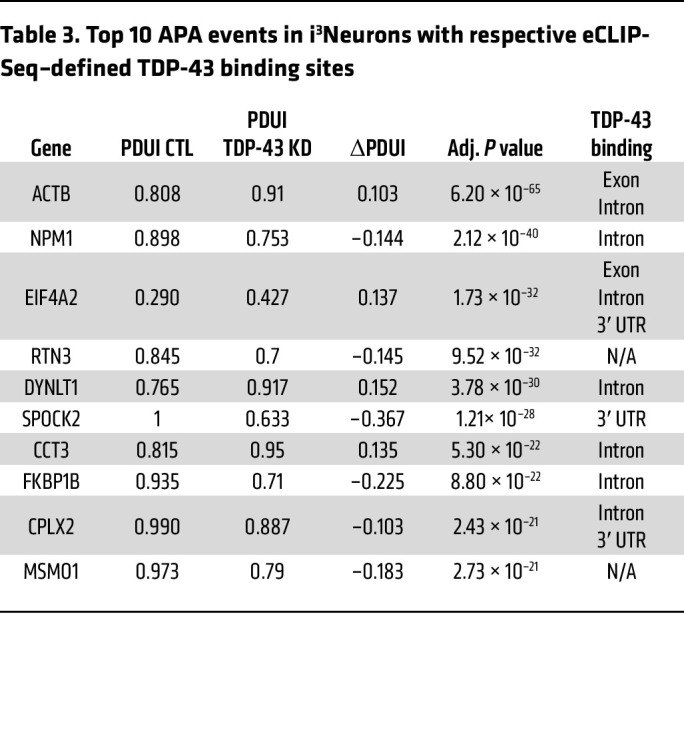
Top 10 APA events in i^3^Neurons with respective eCLIP-Seq–defined TDP-43 binding sites

**Table 5:**
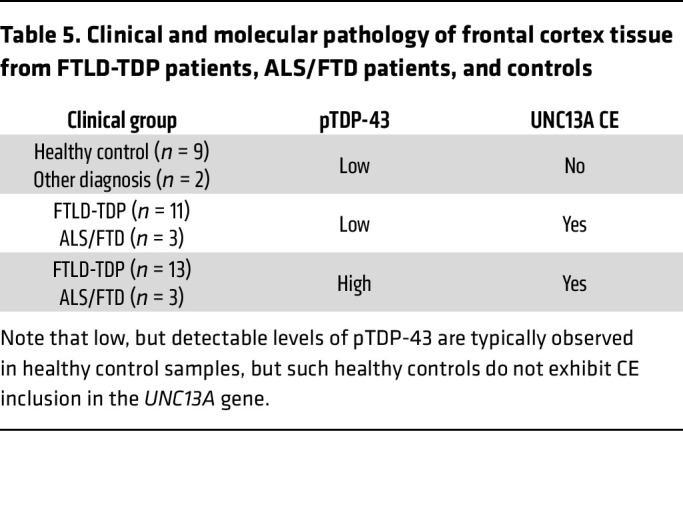
Clinical and molecular pathology of frontal cortex tissue from FTLD-TDP patients, ALS/FTD patients, and controls

**Table 2:**
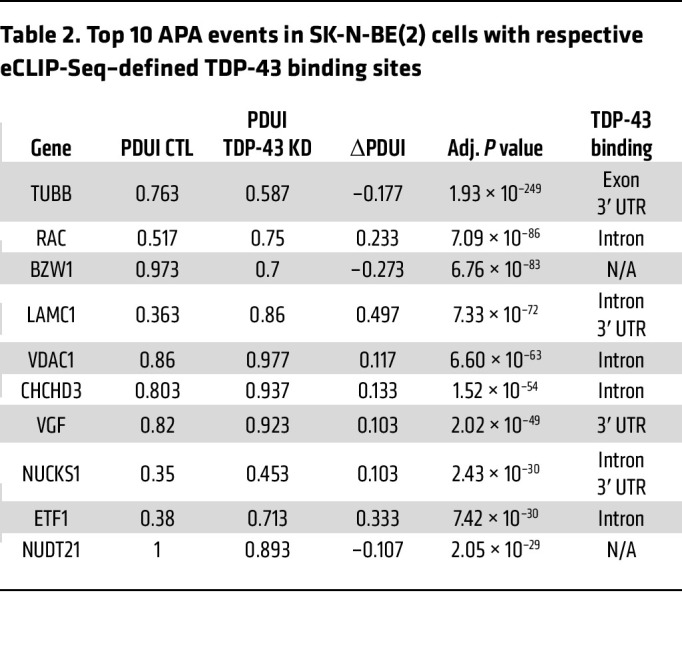
Top 10 APA events in SK-N-BE(2) cells with respective eCLIP-Seq–defined TDP-43 binding sites

**Table 1:**
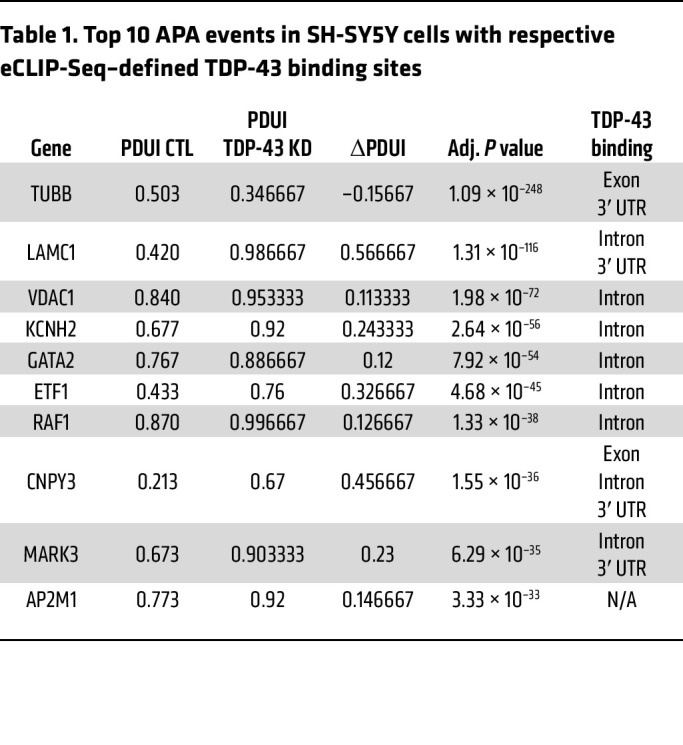
Top 10 APA events in SH-SY5Y cells with respective eCLIP-Seq–defined TDP-43 binding sites

**Table 4:**
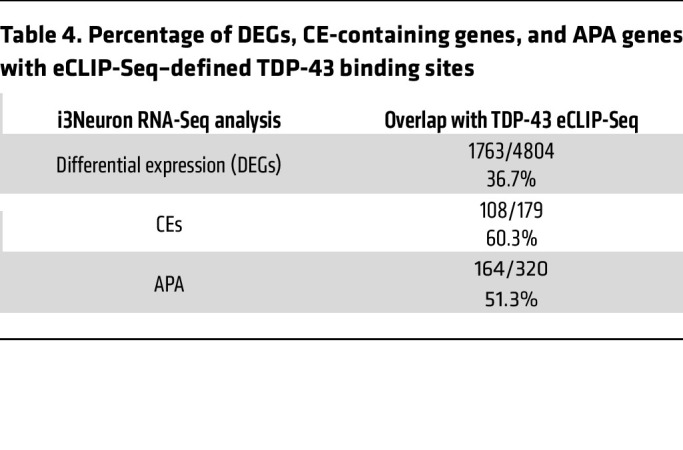
Percentage of DEGs, CE-containing genes, and APA genes with eCLIP-Seq–defined TDP-43 binding sites

**Table 6:**
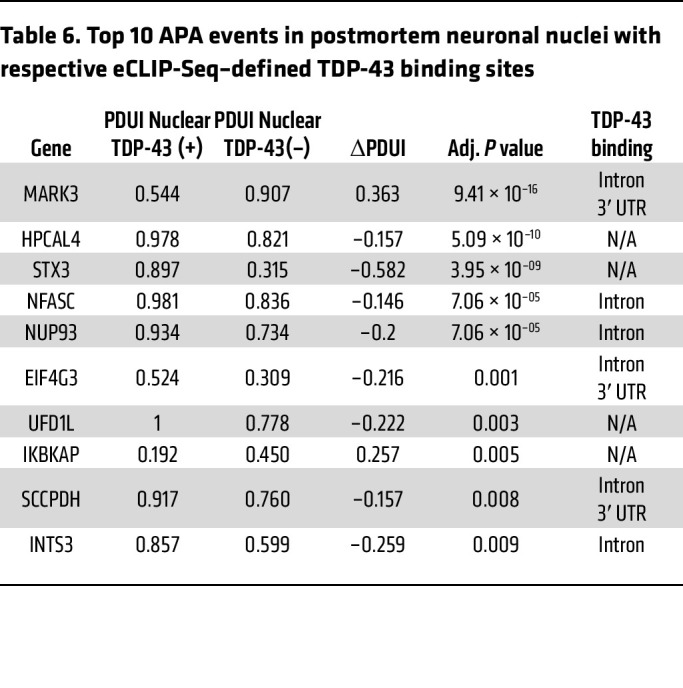
Top 10 APA events in postmortem neuronal nuclei with respective eCLIP-Seq–defined TDP-43 binding sites

**Table 7:**
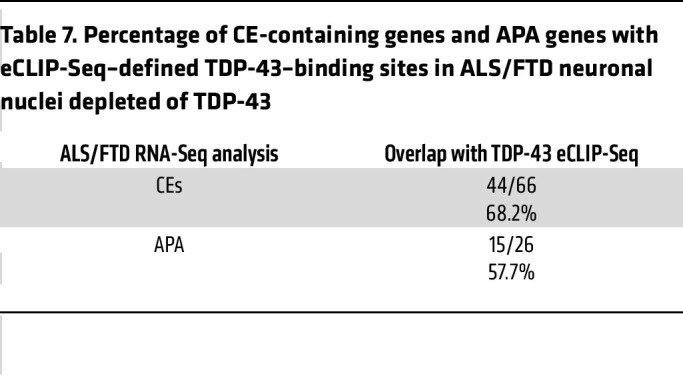
Percentage of CE-containing genes and APA genes with eCLIP-Seq–defined TDP-43–binding sites in ALS/FTD neuronal nuclei depleted of TDP-43

## Version 1 06/02/2025

Electronic publication
