# Supplementary material for: TDP-43 dysregulation of polyadenylation site selection is a defining feature of RNA misprocessing in amyotrophic lateral sclerosis and frontotemporal dementia
Source: J Clin Invest. 2025 Jun 2;135(11):e182088. doi: 10.1172/JCI182088 (PMC12126230; doi:10.1172/JCI182088)
Supplement: Supplemental data [file jci-135-182088-s015.pdf]

Supplementary Figure Legends

Figure S1: APA events in SH-SY5Y cells and hESC-derived MNs upon TDP-43 knock-down
(**A**) Table of top 10 APA events in SH-SY5Y cells treated with control siRNA or TARDBP siRNA for 96 hrs. (**B**) Depiction of ΔPDUI for all significant APA genes in TDP-43 depleted SH-SY5Y cells. (**C**) Overlapping APA genes between siRNA-treated SH-SY5Y cells and doxycycline-induced TARDBP knockdown SH-SY5Y cells, filtering for $p < 0.05$ and $|\Delta PDUI| \geq 0.1$. (**D**) Table of top 10 APA events in hESC-derived motor neurons (ESC-MNs) treated with control siRNA or TARDBP siRNA. Note that TARDBP siRNA treated ESC-MNs displayed only a partial (~60%) reduction in TDP-43 protein levels. (**E**) Depiction of ΔPDUI for all significant APA genes in TDP-43 KD ESC-MNs.

Figure S2: Genes affected by APA function in ALS-relevant pathways
Gene ontology (GO) analysis of biological processes (BP) and molecular functions (MF) of coding APA genes with $p < 0.05$ and $|\Delta PDUI| \geq 0.1$ in (**A, B**) SH-SY5Y cells, (**C, D**) SK-N-BE(2) cells, and (**E**) i^{3}Neurons (note that GO MF terms were not significantly enriched in i^{3}Neurons). Analysis was performed using ShinyGO v0.77.

Figure S3: APA genes are bound by TDP-43 outside of the 3'UTR
Venn diagram illustrating the intersection of APA events between SH-SY5Y cells, SK-N-BE(2) cells, and i^{3}Neurons depleted of TDP-43, along with evidence for direct TDP-43 binding in regions outside of the 3'UTR, in which eCLIP-seq data show TDP-43 binding within the 5'UTR, in introns, or in exons of APA genes.

Figure S4: RNA-sequencing tracks depict APA for the *CNPY3* and *SMC1A* genes in the presence or absence of TDP-43 knock-down
RNA-seq tracks for *CNPY3* (**A**) and *SMC1A* (**B**) in i^{3}Neurons, SH-SY5Y cells, and SK-N-BE(2) cells in the presence or absence of TDP-43 knock-down. The location of the TDP-43 3'UTR binding site is depicted in green (*CNPY3* at chr6: 42,935,715 - 42,935,762; *SMC1A* at chrX: 53,379,722 - 53,379,785).

Figure S5: Isoform specific quantification of CNPY3 APA in the presence or absence of TDP-43 knockdown.
qRT-PCR quantification of CNPY3 variant 1 and variant 2 in (**A**) SH-SY5Y cells or (**B**) i^{3}Neurons in the presence of absence of TDP-43 knockdown. $^{**}p < 0.01$; unpaired two-tailed t-test; $n = 3$ biological replicates. Data are presented as mean values ± s.e.m.

Figure S6: Validation of TDP-43 knockdown in i3Neurons and iPSC-MNs.

Immunoblot of i3Neurons (**A**) and iPSC-MNs (**B**) in which TDP-43 was knocked down with shRNAs for 10 days. ****p<0.0001, one-way ANOVA (**A**), unpaired two-tailed t-test (**B**); n=3 biological replicates. Error bars = s.e.m.

Figure S7: Gene ontology (GO) analysis of coding APA events in ALS/FTD postmortem neuronal nuclei depleted of TDP-43.

GO analysis of biological processes enriched for coding APA events with $p < 0.05$ and $|\Delta PDUI| \geq 0.1$ highlights genes that function in nucleocytoplasmic transport and synapse assembly.

Figure S8: The MARK3 3'UTR contains two conserved RBP binding motifs

(**A**) Predicted RBP binding motifs in the MARK3 3'UTR with $p < 0.001$ and Z-score > 3.5 for at least one motif (RBPmap). (**B**) Graphical representation of select conserved cis-regulatory elements in the MARK3 3'UTR.

Figure S9: **Quantification of MARK3 APA in ALS/FTD and FTLD-TDP frontal cortex.**

qRT-PCR quantification of (**A**) the short 3'UTR MARK3 isoform, (**B**) the long-3'UTR MARK3 isoform, and (**C**) the ratio of long:short 3'UTR isoforms in postmortem frontal cortex tissue from controls or from ALS/FTD and FTLD-TDP patients with confirmed inclusion of the *UNC13A* CE (as described in **Fig. 3E**). *p<0.05; unpaired two-tailed t-test; n = 11 (control), n = 30 (*UNC13A* CE). Data are presented as mean values ± s.e.m.

Figure S10: TDP-43 knock-down in iPSC-MNs corresponds with a trend towards increased tau S262 phosphorylation.

(**A**) Immunoblot of S262 phosphorylated tau and total tau in iPSC-MNs transduced with lentivirus encoding control shRNA or shRNA TARDBP for 10 days. Statistical significance was determined by one sample t-test; n=3 biological replicates. (**B**) Immunoblot of MARK3, S262 phosphorylated tau, and total tau in iPSC-MNs transduced with lentivirus encoding either control shRNA, MARK3 shRNA, GFP (empty vector), or MARK3-V5 for 10 days. Statistical significance was determined by unpaired two-tailed t-test; n=3 biological replicates. *p<0.05, ***p<0.001; unpaired two-tailed t-test; n = 3 biological replicates. Error bars = s.e.m. (**C**) Immunoblot of MARK3 in iPSC-MNs transduced with lentivirus encoding control shRNA, TARDBP shRNA, or MARK3 shRNA for 10 days. Statistical significance was determined by unpaired two-tailed t-test; n=3 biological replicates. Error bars = s.e.m.

Figure S11: TDP-43 knock-down does not significantly increase MARK3 or MARK4 protein levels in i^{3}Neurons or in postmortem ALS/FTD or FTLD-TDP patient tissue.

(**A**) Immunoblot of MARK3 in i^{3}Neurons depleted of TDP-43 with shRNA for 10 days. n=3 biological replicates. Statistical significance was determined by one-way ANOVA; p=n.s. Note that the lower molecular weight portion of this blot is shown in Figure S6; thus, the same total protein blot was used for quantification. (**B**) Quantification of immunoblots for MARK3 protein in the frontal cortex of healthy controls (n=5) or ALS/FTD and FTLD-TDP patients (n=11). Statistical significance was determined by unpaired two-tailed t-test; p=n.s. (**C**) Immunoblot of MARK4 in i^{3}Neurons depleted of TDP-43 with shRNAs for 10 days. Statistical significance was determined by one sample t-test against a value of 1; p=n.s. Error bars = s.e.m.

A

Gene	PDUI CTL	PDUI KD	ΔPDUI	p-value	Adj. p-value	TDP-43 binding
ILF2	0.71	0.86	0.15	7.10E-10	1.22E-06	N/A
RPN2	0.883	1	0.117	8.77E-10	1.22E-06	Intron
HNRNPA1P10	0.75	0.86	0.11	1.26E-09	1.40E-06	Exon
SMC1A	0.28	0.0733	-0.207	1.44E-06	0.000616	Intron **3'UTR**
PLD3	0.783	0.89	0.107	5.60E-05	0.0195	N/A
COASY	0.78	0.987	0.207	0.00012	0.0381	N/A
SSNA1	0.883	1	0.117	0.00015	0.0477	N/A
ACBD6	0.72	0.923	0.203	0.00024	0.0659	Intron
PNKD	0.817	0.987	0.17	0.00028	0.0754	N/A
STRA13	0.757	0.897	0.14	0.00045	0.104	N/A

D

Gene	PDUI CTL	PDUI KD	ΔPDUI	p-value	Adj. p-value	TDP-43 binding
SMC1A	0.722	0.467	-0.255	0.0064	1	Intron 3'UTR
GPCPD1	0.783	0.542	-0.242	0.0055	1	N/A
PTPLB	0.907	0.692	-0.215	0.011	1	N/A
CDK6	0.972	0.780	-0.192	0.0053	1	Intron Exon 3'UTR
DIDO1	0.515	0.327	-0.188	0.043	1	Intron
AIG1	0.835	0.652	-0.183	0.033	1	Intron
PLEKHM2	0.718	0.538	-0.180	0.030	1	N/A
RANGRF	0.957	0.783	-0.173	0.0009	0.55	N/A
TMEM11	0.937	0.765	-0.172	0.0004	0.37	N/A
TFIP11	0.912	0.740	-0.172	0.0093	1	N/A

B

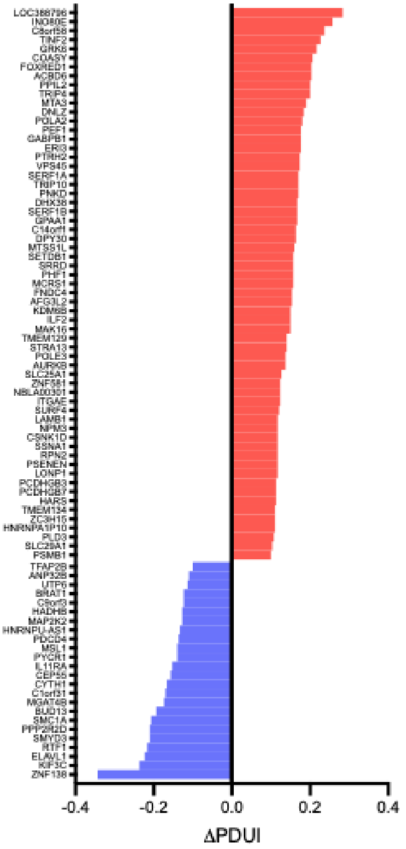

E

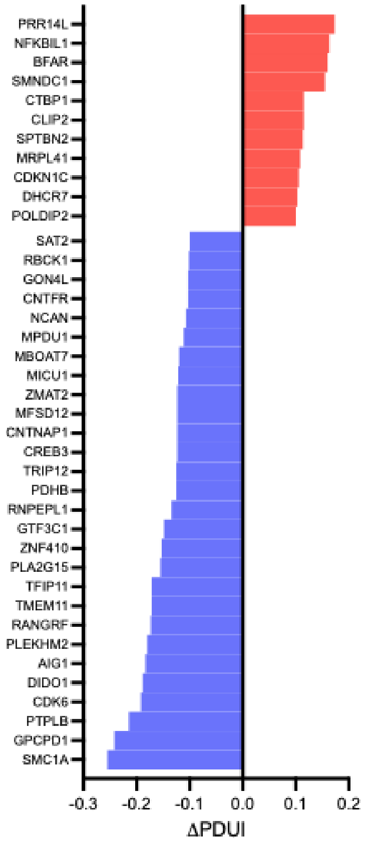

C

Overlap with Brown et. al, 2022
ACBD6
ELAVL1
GRK6
INO80E
NPM3
PPP2R2D
PTRH2
SMC1A
ZNF138

Figure S1

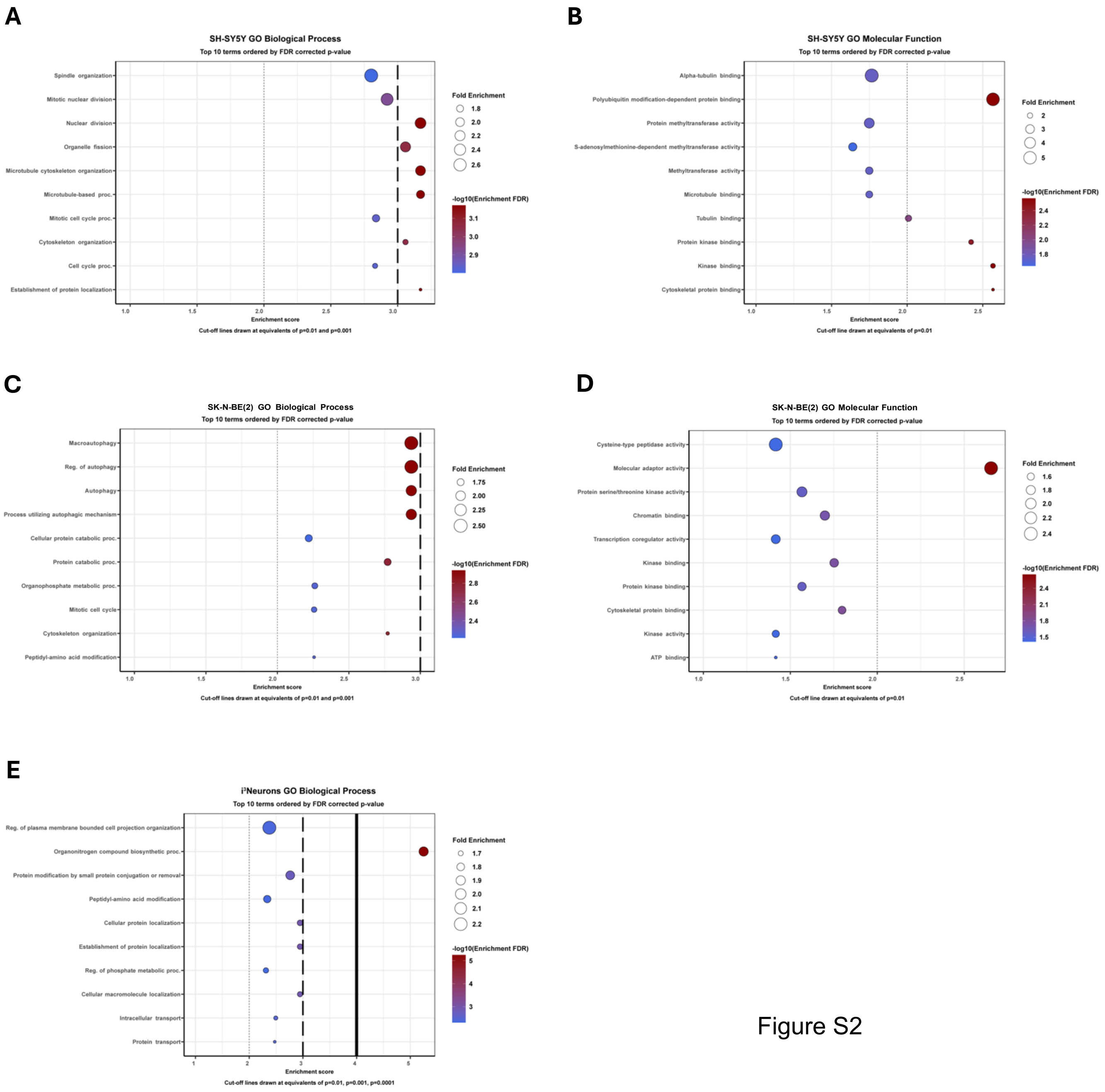
A
SH-SY5Y GO Biological Process
Top 10 terms ordered by FDR corrected p-value
Spindle organization
Mitotic nuclear division
Nuclear division
Organelle fission
Microtubule cytoskeleton organization
Microtubule-based proc.
Mitotic cell cycle proc.
Cytoskeleton organization
Cell cycle proc.
Establishment of protein localization
Fold Enrichment
-log10(Enrichment FDR)
Enrichment score
Cut-off lines drawn at equivalents of p=0.01 and p=0.001
B
SH-SY5Y GO Molecular Function
Top 10 terms ordered by FDR corrected p-value
Alpha-tubulin binding
Polyubiquitin modification-dependent protein binding
Protein methyltransferase activity
S-adenosylmethionine-dependent methyltransferase activity
Methyltransferase activity
Microtubule binding
Tubulin binding
Protein kinase binding
Kinase binding
Cytoskeletal protein binding
Fold Enrichment
-log10(Enrichment FDR)
Enrichment score
Cut-off line drawn at equivalents of p=0.01
C
SK-N-BE(2) GO Biological Process
Top 10 terms ordered by FDR corrected p-value
Macroautophagy
Reg. of autophagy
Autophagy
Process utilizing autophagic mechanism
Cellular protein catabolic proc.
Protein catabolic proc.
Organophosphate metabolic proc.
Mitotic cell cycle
Cytoskeleton organization
Peptidyl-amino acid modification
Fold Enrichment
-log10(Enrichment FDR)
Enrichment score
Cut-off lines drawn at equivalents of p=0.01 and p=0.001
D
SK-N-BE(2) GO Molecular Function
Top 10 terms ordered by FDR corrected p-value
Cysteine-type peptidase activity
Molecular adaptor activity
Protein serine/threonine kinase activity
Chromatin binding
Transcription coregulator activity
Kinase binding
Protein kinase binding
Cytoskeletal protein binding
Kinase activity
ATP binding
Fold Enrichment
-log10(Enrichment FDR)
Enrichment score
Cut-off line drawn at equivalents of p=0.01
E
i³Neurons GO Biological Process
Top 10 terms ordered by FDR corrected p-value
Reg. of plasma membrane bounded cell projection organization
Organonitrogen compound biosynthetic proc.
Protein modification by small protein conjugation or removal
Peptidyl-amino acid modification
Cellular protein localization
Establishment of protein localization
Reg. of phosphate metabolic proc.
Cellular macromolecule localization
Intracellular transport
Protein transport
Fold Enrichment
-log10(Enrichment FDR)
Enrichment score
Cut-off lines drawn at equivalents of p=0.01, p=0.001, p=0.0001

Figure S2

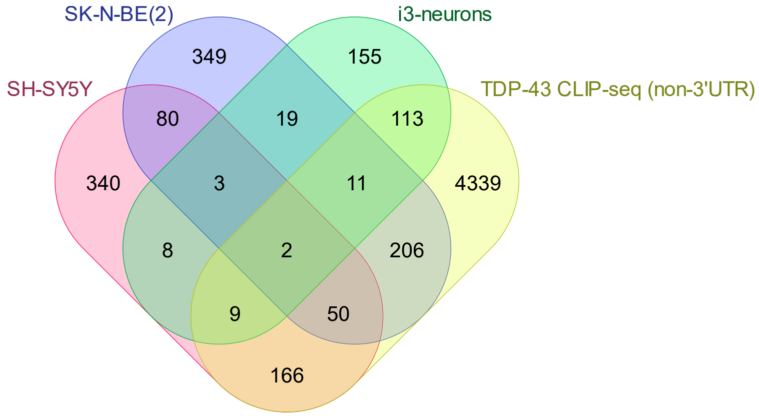
SK-N-BE(2)
i3-neurons
SH-SY5Y
TDP-43 CLIP-seq (non-3'UTR)
349
155
80
19
113
340
3
11
4339
8
2
206
9
50
166

Figure S3

Figure S4

A

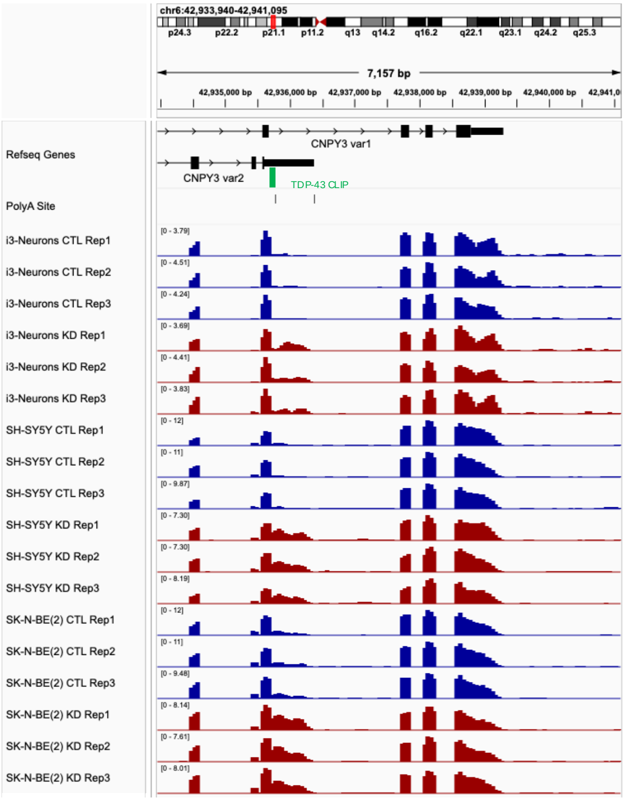
chr6:42,933,940-42,941,095
p24.3
p22.2
p21.1
p11.2
q13
q14.2
q16.2
q22.1
q23.1
q24.2
q25.3
7,157 bp
42,935,000 bp
42,936,000 bp
42,937,000 bp
42,938,000 bp
42,939,000 bp
42,940,000 bp
Refseq Genes
CNPY3 var1
CNPY3 var2
TDP-43 CLIP
PolyA Site
i3-Neurons CTL Rep1
i3-Neurons CTL Rep2
i3-Neurons CTL Rep3
i3-Neurons KD Rep1
i3-Neurons KD Rep2
i3-Neurons KD Rep3
SH-SY5Y CTL Rep1
SH-SY5Y CTL Rep2
SH-SY5Y CTL Rep3
SH-SY5Y KD Rep1
SH-SY5Y KD Rep2
SH-SY5Y KD Rep3
SK-N-BE(2) CTL Rep1
SK-N-BE(2) CTL Rep2
SK-N-BE(2) CTL Rep3
SK-N-BE(2) KD Rep1
SK-N-BE(2) KD Rep2
SK-N-BE(2) KD Rep3

B

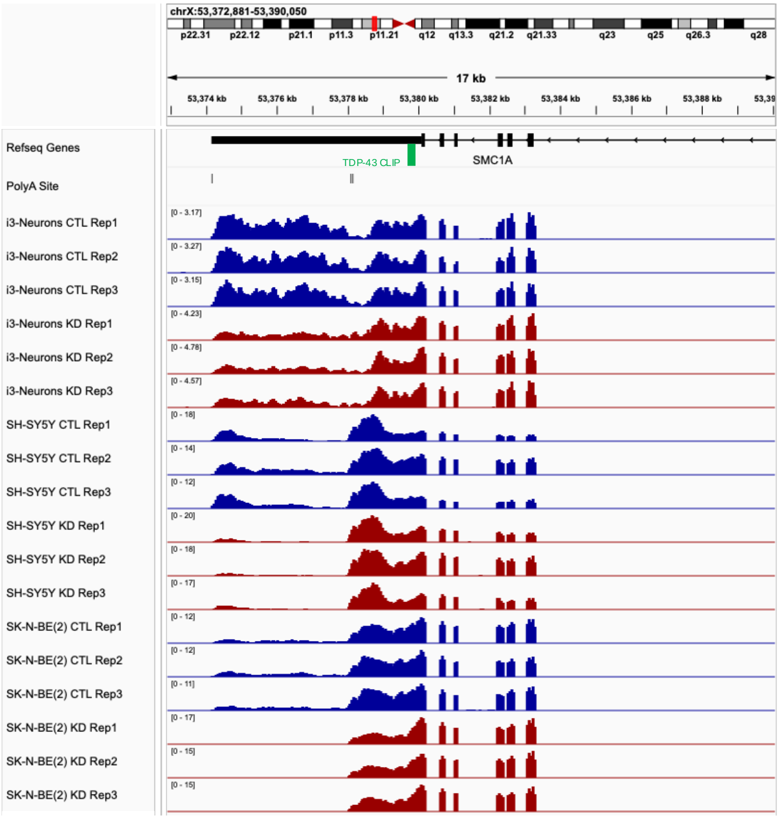
chrX:53,372,881-53,390,050
p22.31
p22.12
p21.1
p11.3
p11.21
q12
q13.3
q21.2
q21.33
q23
q25
q26.3
q28
17 kb
53,374 kb
53,376 kb
53,378 kb
53,380 kb
53,382 kb
53,384 kb
53,386 kb
53,388 kb
Refseq Genes
TDP-43 CLIP
SMC1A
PolyA Site
i3-Neurons CTL Rep1
i3-Neurons CTL Rep2
i3-Neurons CTL Rep3
i3-Neurons KD Rep1
i3-Neurons KD Rep2
i3-Neurons KD Rep3
SH-SY5Y CTL Rep1
SH-SY5Y CTL Rep2
SH-SY5Y CTL Rep3
SH-SY5Y KD Rep1
SH-SY5Y KD Rep2
SH-SY5Y KD Rep3
SK-N-BE(2) CTL Rep1
SK-N-BE(2) CTL Rep2
SK-N-BE(2) CTL Rep3
SK-N-BE(2) KD Rep1
SK-N-BE(2) KD Rep2
SK-N-BE(2) KD Rep3

Figure S5

A

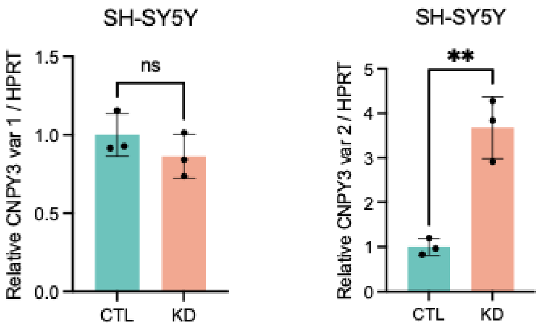
SH-SY5Y
Relative CNPY3 var 1 / HPRT
1.5
1.0
0.5
0.0
ns
CTL
KD
SH-SY5Y
Relative CNPY3 var 2 / HPRT
5
4
3
2
1
0
**
CTL
KD

B

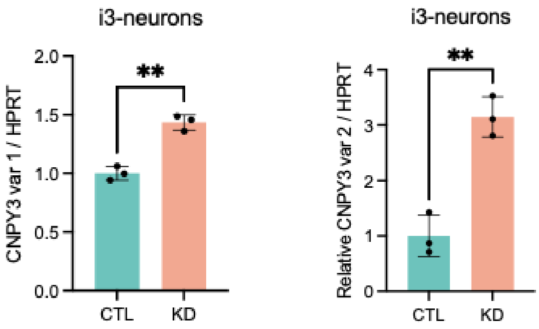
i3-neurons
CNPY3 var 1 / HPRT
2.0
1.5
1.0
0.5
0.0
**
CTL
KD
i3-neurons
Relative CNPY3 var 2 / HPRT
4
3
2
1
0
**
CTL
KD

A

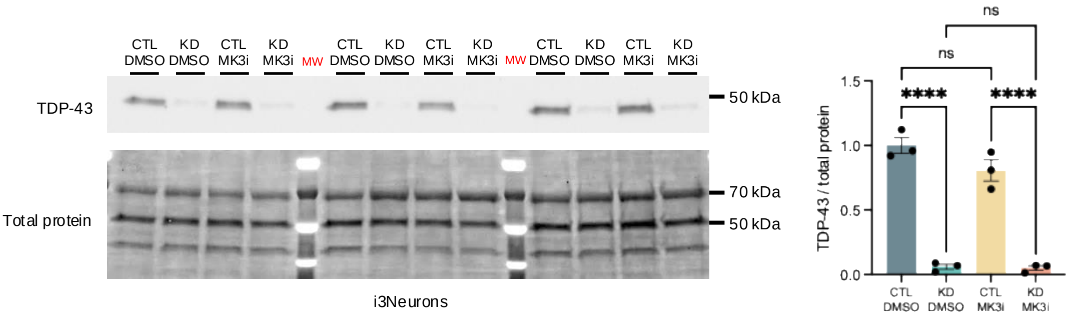
CTL DMSO
KD DMSO
CTL MK3i
KD MK3i
MW
TDP-43
Total protein
50 kDa
70 kDa
50 kDa
i3Neurons
ns
ns
****
****
1.5
1.0
0.5
0.0
TDP-43 / total protein
CTL DMSO
KD DMSO
CTL MK3i
KD MK3i

B

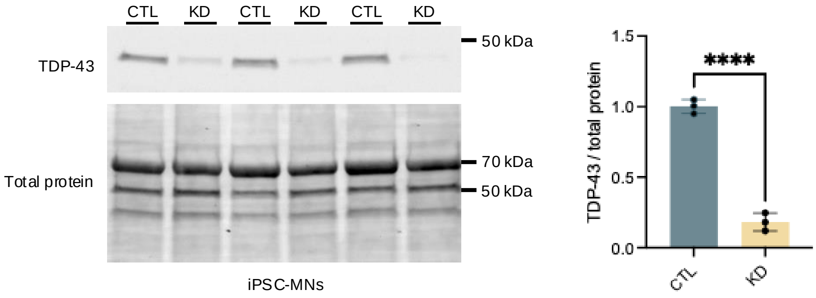
CTL
KD
CTL
KD
CTL
KD
TDP-43
Total protein
50 kDa
70 kDa
50 kDa
iPSC-MNs
1.5
1.0
0.5
0.0
****
TDP-43 / total protein
CTL
KD

Figure S6

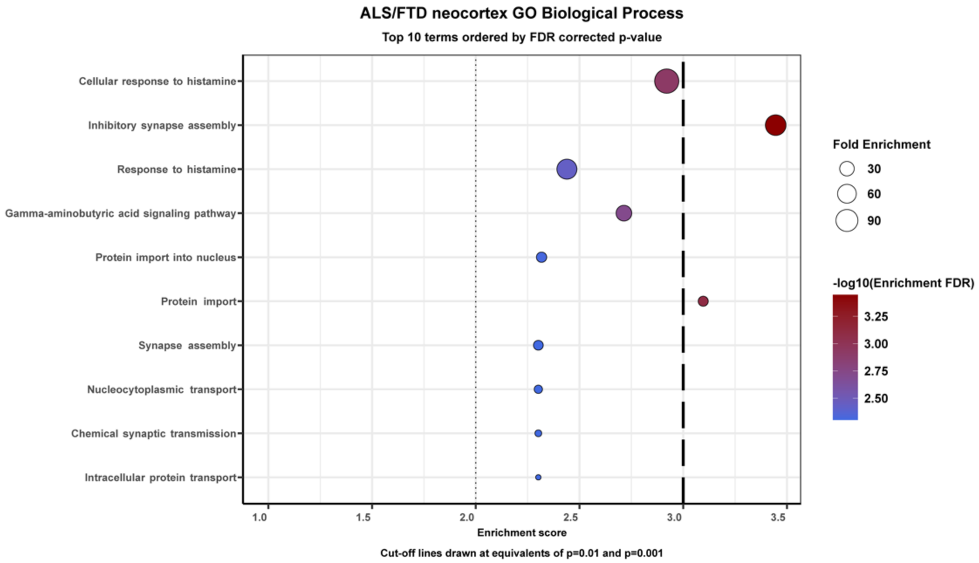

ALS/FTD neocortex GO Biological Process
Top 10 terms ordered by FDR corrected p-value
Cellular response to histamine
Inhibitory synapse assembly
Response to histamine
Gamma-aminobutyric acid signaling pathway
Protein import into nucleus
Protein import
Synapse assembly
Nucleocytoplasmic transport
Chemical synaptic transmission
Intracellular protein transport
1.0
1.5
2.0
2.5
3.0
3.5
Enrichment score
Cut-off lines drawn at equivalents of p=0.01 and p=0.001
Fold Enrichment
30
60
90
-log10(Enrichment FDR)
3.25
3.00
2.75
2.50

Figure S7

A

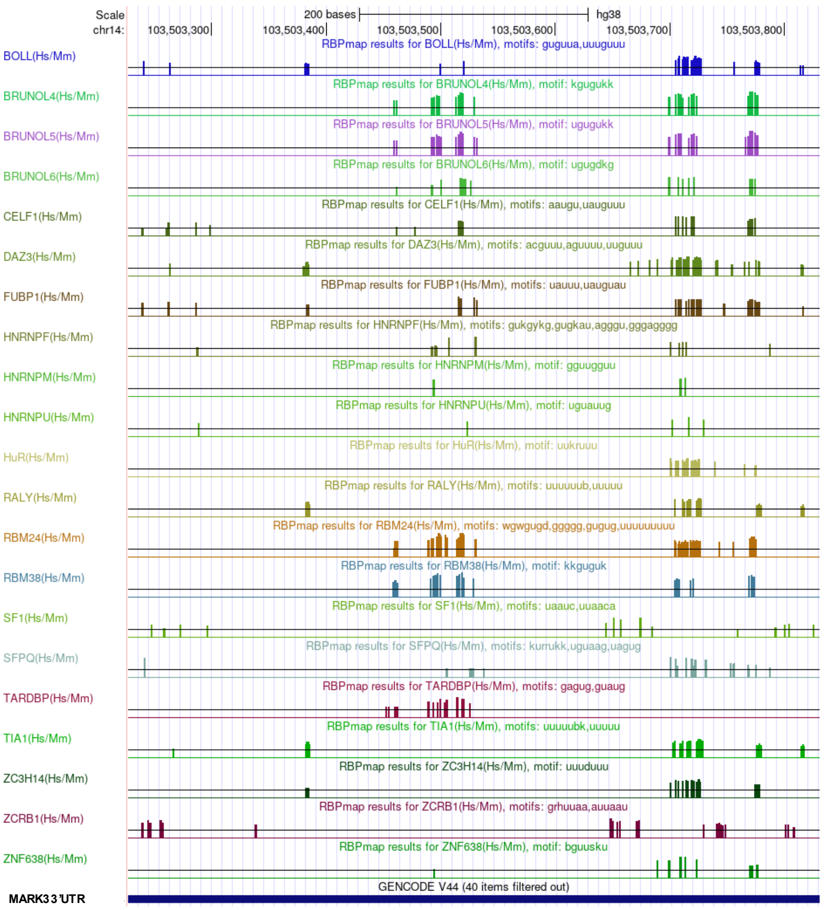

Scale
chr14:
200 bases
hg38
103,503,300
103,503,400
103,503,500
103,503,600
103,503,700
103,503,800
BOLL(Hs/Mm)
RBPmap results for BOLL(Hs/Mm), motifs: guguua,uuuguuu
BRUNOL4(Hs/Mm)
RBPmap results for BRUNOL4(Hs/Mm), motif: kgugukk
BRUNOL5(Hs/Mm)
RBPmap results for BRUNOL5(Hs/Mm), motif: ugugukk
BRUNOL6(Hs/Mm)
RBPmap results for BRUNOL6(Hs/Mm), motif: ugugdkg
CELF1(Hs/Mm)
RBPmap results for CELF1(Hs/Mm), motifs: aaugu,uauguuu
DAZ3(Hs/Mm)
RBPmap results for DAZ3(Hs/Mm), motifs: acguuu,aguuuu,uuguuu
FUBP1(Hs/Mm)
RBPmap results for FUBP1(Hs/Mm), motifs: uauuu,uauguau
HNRNPF(Hs/Mm)
RBPmap results for HNRNPF(Hs/Mm), motifs: gukgykg,gugkau,agggu,gggagggg
HNRNPM(Hs/Mm)
RBPmap results for HNRNPM(Hs/Mm), motif: gguuggu
HNRNPU(Hs/Mm)
RBPmap results for HNRNPU(Hs/Mm), motif: uguauug
HuR(Hs/Mm)
RBPmap results for HuR(Hs/Mm), motif: uukruuu
RALY(Hs/Mm)
RBPmap results for RALY(Hs/Mm), motifs: uuuuuub,uuuuu
RBM24(Hs/Mm)
RBPmap results for RBM24(Hs/Mm), motifs: wgwgugd,ggggg,gugug,uuuuuuuuu
RBM38(Hs/Mm)
RBPmap results for RBM38(Hs/Mm), motif: kkguguk
SF1(Hs/Mm)
RBPmap results for SF1(Hs/Mm), motifs: uaauc,uuaaca
SFPQ(Hs/Mm)
RBPmap results for SFPQ(Hs/Mm), motifs: kurrukk,uguaag,uagug
TARDBP(Hs/Mm)
RBPmap results for TARDBP(Hs/Mm), motifs: gagug,guaug
TIA1(Hs/Mm)
RBPmap results for TIA1(Hs/Mm), motifs: uuuuubk,uuuuu
ZC3H14(Hs/Mm)
RBPmap results for ZC3H14(Hs/Mm), motif: uuuduuu
ZCRB1(Hs/Mm)
RBPmap results for ZCRB1(Hs/Mm), motifs: grhuuaa,auuaau
ZNF638(Hs/Mm)
RBPmap results for ZNF638(Hs/Mm), motif: bguusku
MARK3 3'UTR
GENCODE V44 (40 items filtered out)

B

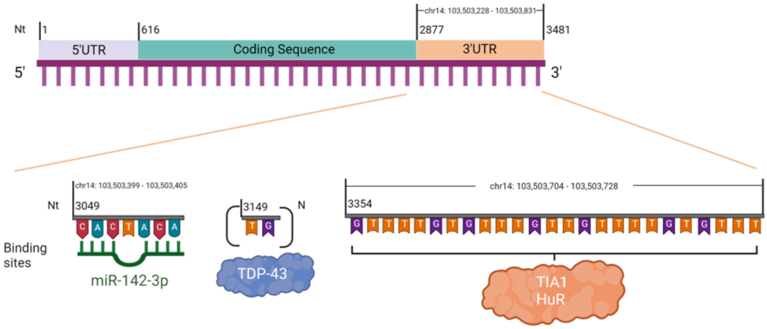

chr14: 103,503,228 - 103,503,831
Nt
1
616
2877
3481
5'UTR
Coding Sequence
3'UTR
5'
3'
chr14: 103,503,399 - 103,503,405
Nt
3049
C A C T A C A
Binding sites
miR-142-3p
3149
N
T G
TDP-43
chr14: 103,503,704 - 103,503,728
3354
TIA1
HuR

Figure S8

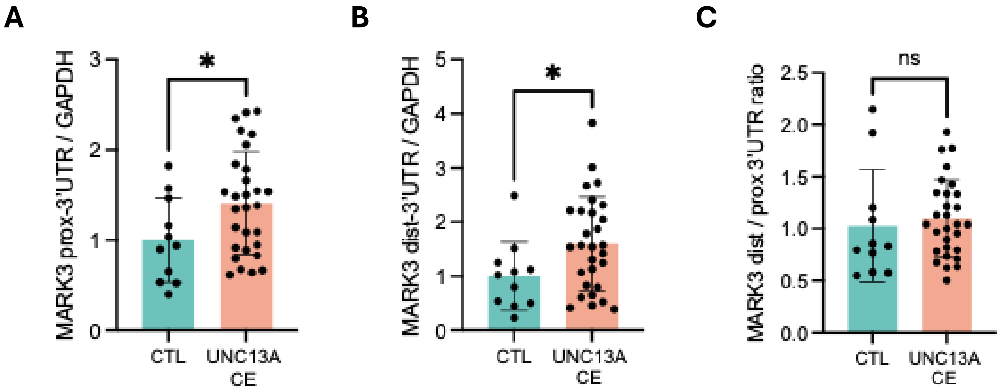
A
B
C
MARK3 prox-3'UTR / GAPDH
MARK3 dist-3'UTR / GAPDH
MARK3 dist / prox 3'UTR ratio
*
*
ns
CTL
UNC13A CE
CTL
UNC13A CE
CTL
UNC13A CE

Figure S9

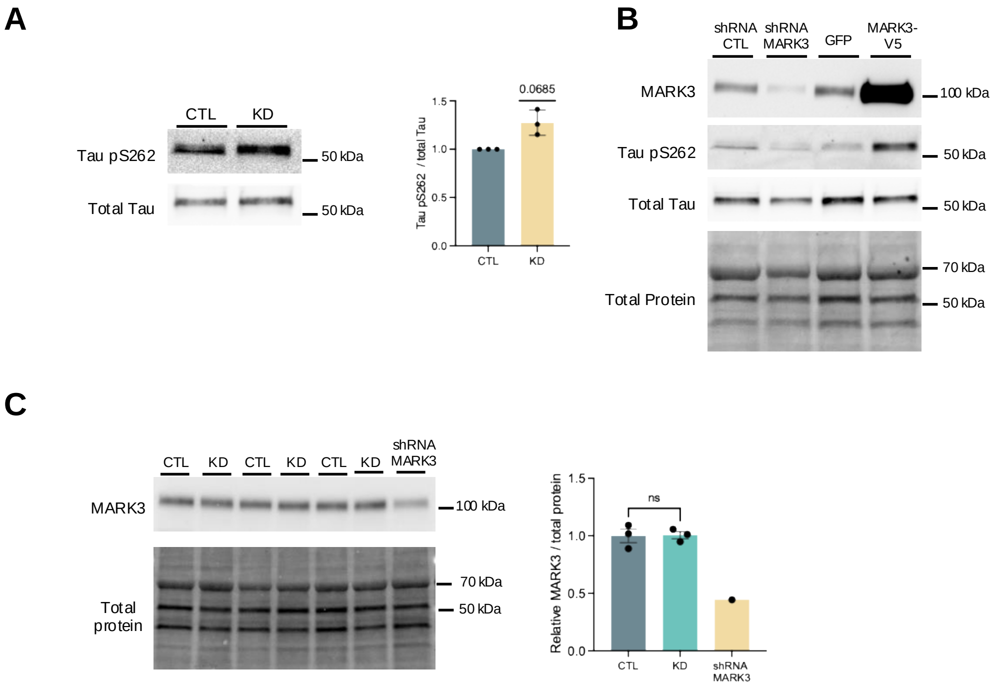
A
CTL
KD
Tau pS262
50 kDa
Total Tau
50 kDa
0.0685
Tau pS262 / total Tau
CTL
KD
B
shRNA CTL
shRNA MARK3
GFP
MARK3-V5
MARK3
100 kDa
Tau pS262
50 kDa
Total Tau
50 kDa
Total Protein
70 kDa
50 kDa
***
*
MARK3 / total protein
shRNA CTL
shRNA MARK3
GFP
MARK3-V5
C
CTL
KD
CTL
KD
CTL
KD
shRNA MARK3
MARK3
100 kDa
Total protein
70 kDa
50 kDa
ns
Relative MARK3 / total protein
CTL
KD
shRNA MARK3

Figure S10

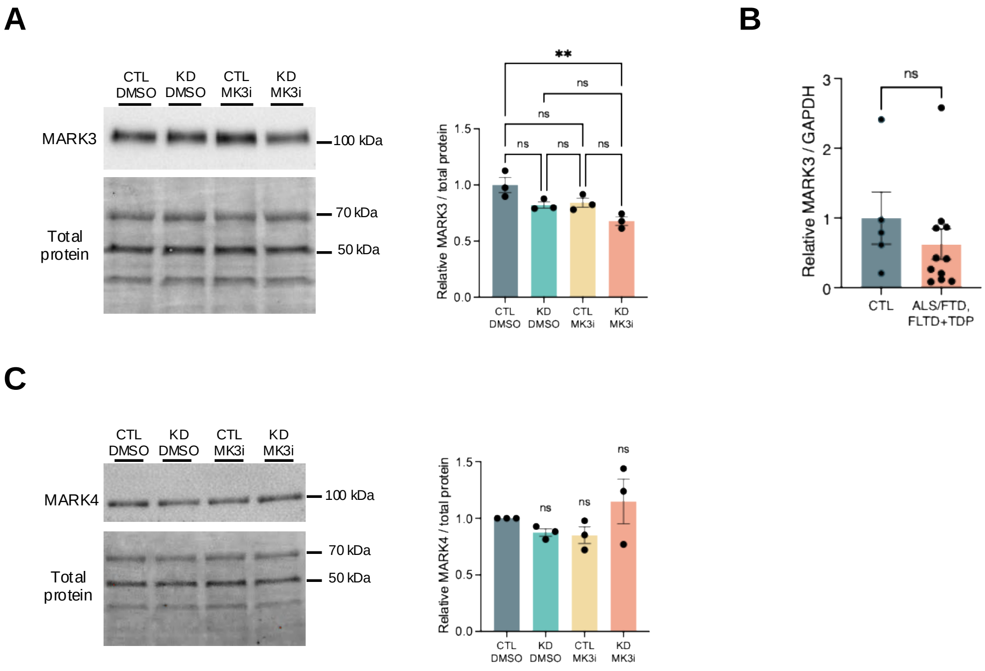
A
CTL DMSO
KD DMSO
CTL MK3i
KD MK3i
MARK3
100 kDa
Total protein
70 kDa
50 kDa
**
ns
ns
ns
ns
ns
Relative MARK3 / total protein
1.5
1.0
0.5
0.0
B
Relative MARK3 / GAPDH
3
2
1
0
ns
CTL
ALS/FTD, FLTD+TDP
C
MARK4
Relative MARK4 / total protein

Figure S11
