# Supplementary material for: TDP-43 dysregulation of polyadenylation site selection is a defining feature of RNA misprocessing in amyotrophic lateral sclerosis and frontotemporal dementia
Source: J Clin Invest. 2025 Jun 2;135(11):e182088. doi: 10.1172/JCI182088 (PMC12126230; doi:10.1172/JCI182088)
Supplement: Unedited blot and gel images [file jci-135-182088-s016.pdf]

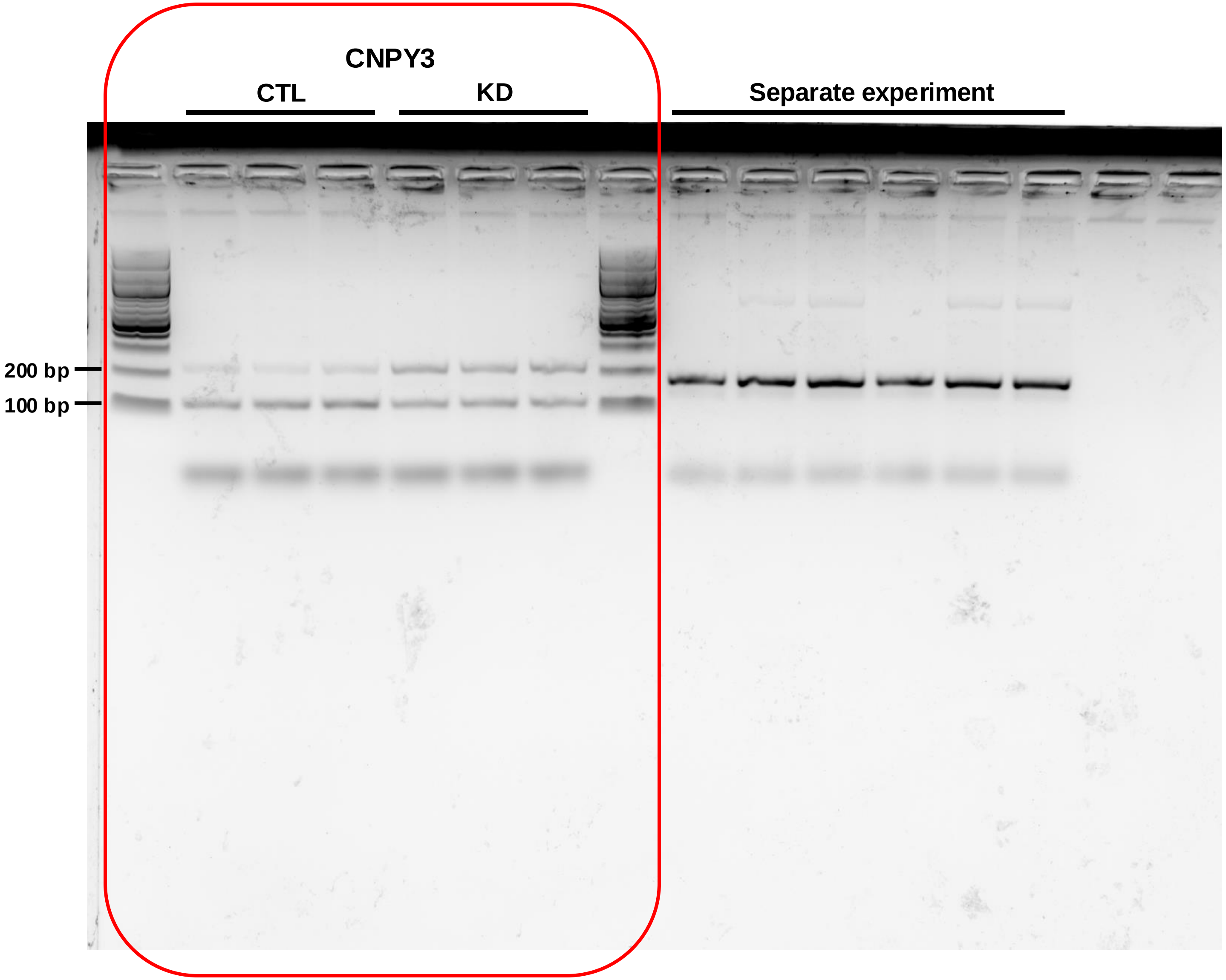

Full unedited gel for Figure 3A. Highlighted bands (red), excluding DNA ladders and primer dimers, were used in Figure 3A.

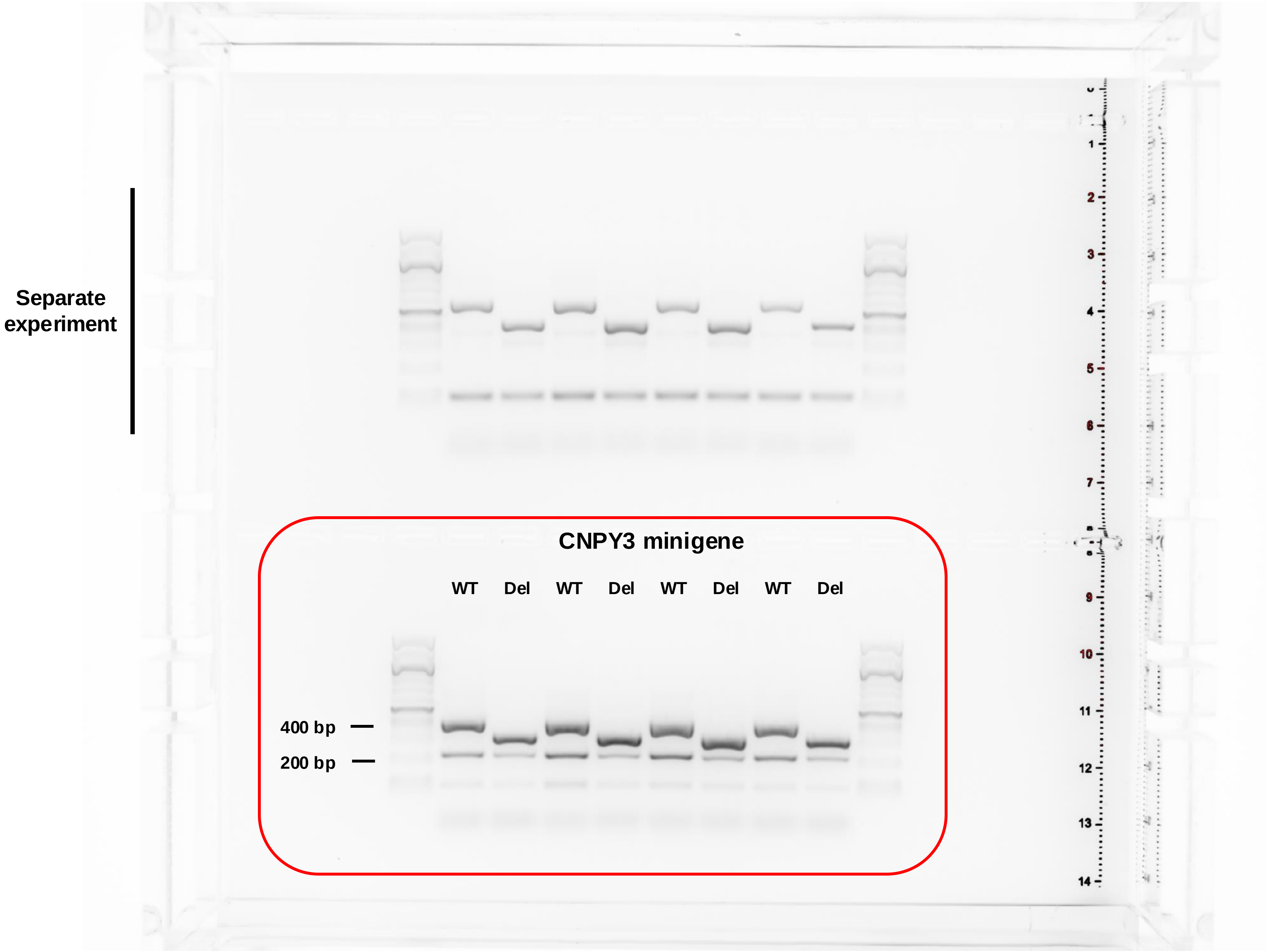

Full unedited gel for Figure 3B. Highlighted bands (red), excluding DNA ladders and primer dimers, were used in Figure 3B.

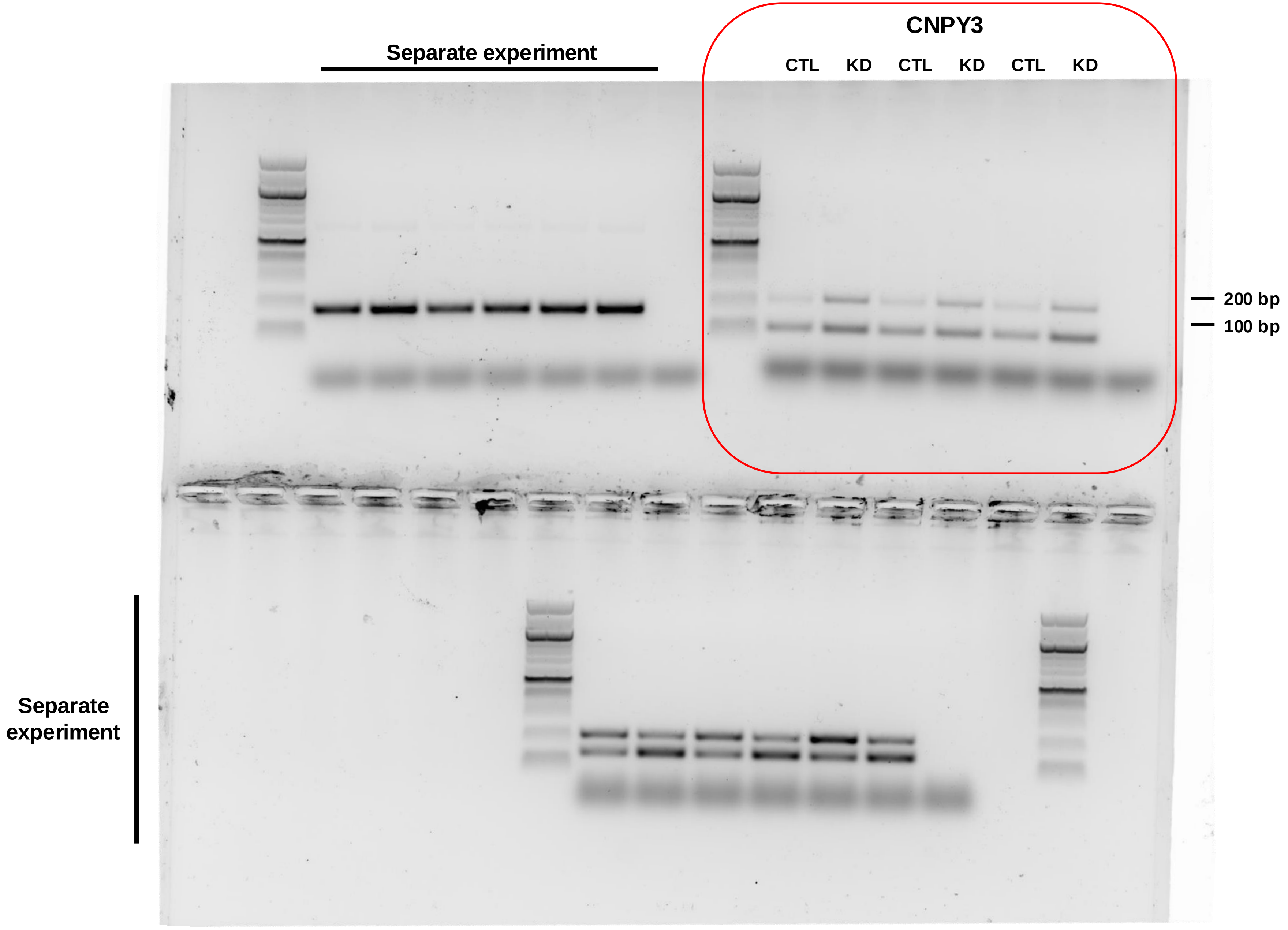

Full unedited gel for Figure 3C. Highlighted bands (red), excluding DNA ladders and primer dimers, were used in Figure 3C.

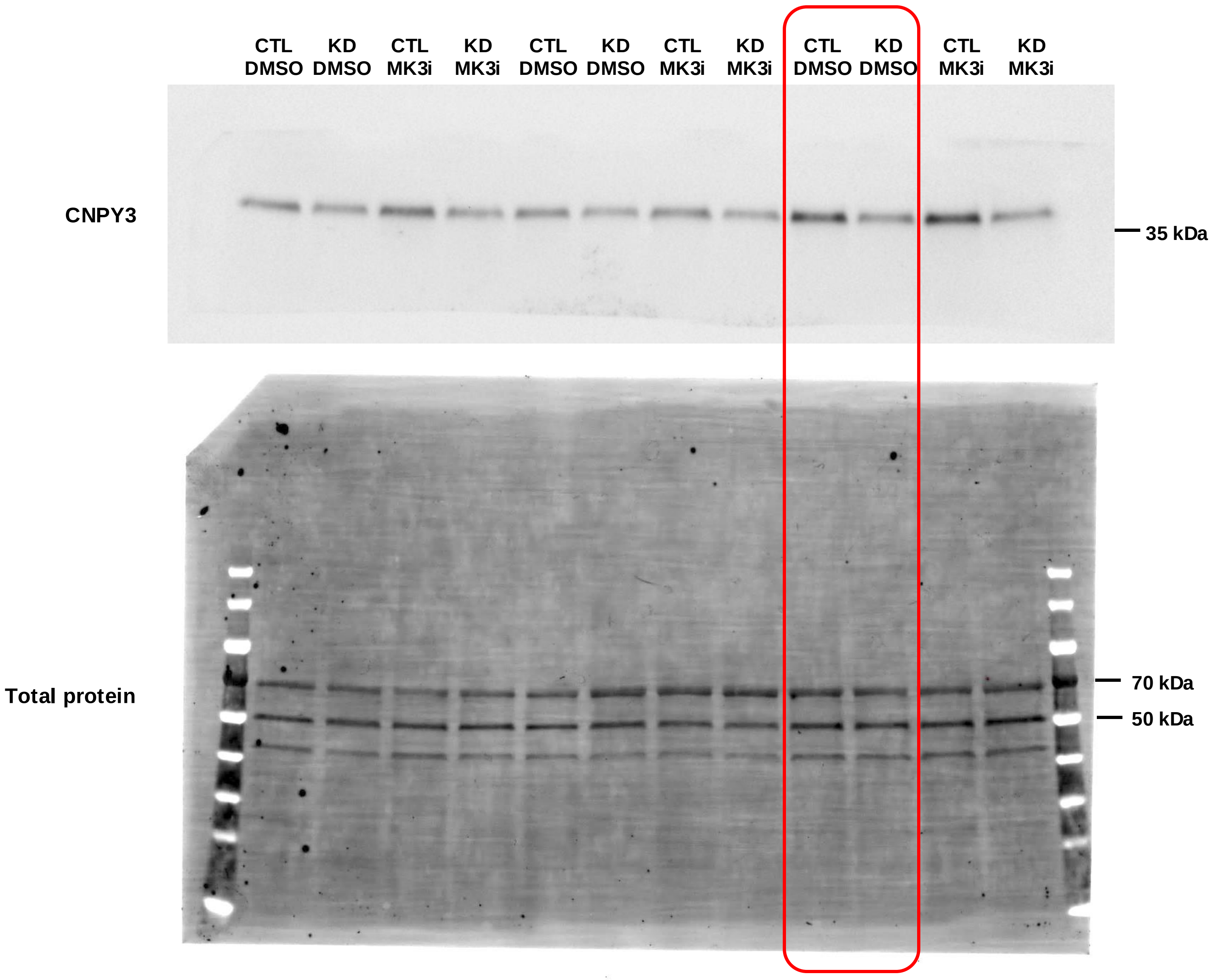

Full unedited blots for Figure 3D. Highlighted bands (red) were used in Figure 3D.

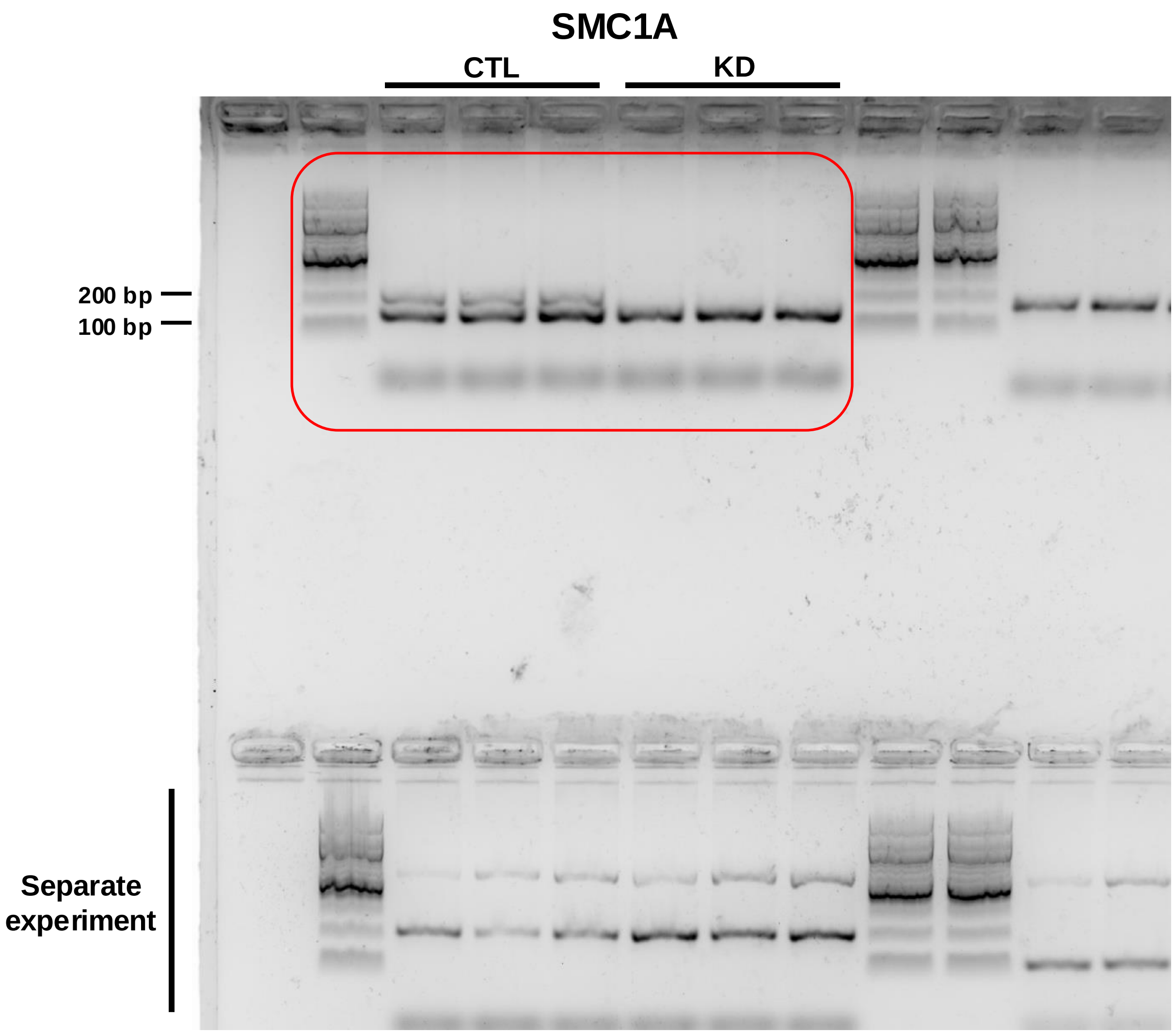

Full unedited gel for Figure 4A. Highlighted bands (red), excluding DNA ladders and primer dimers, were used in Figure 4A.

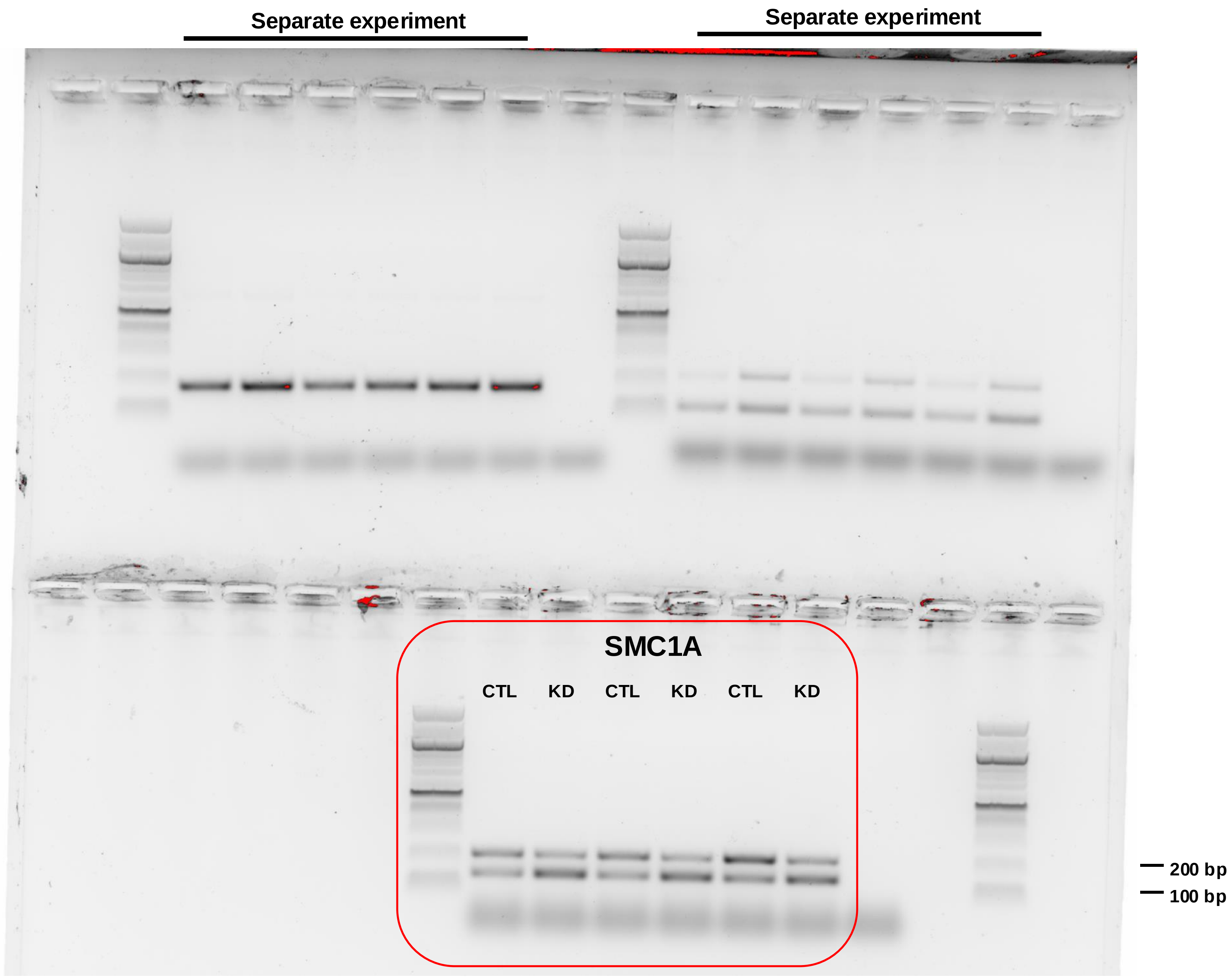

Full unedited gel for Figure 4C. Highlighted bands (red), excluding DNA ladders and primer dimers, were used in Figure 4C.

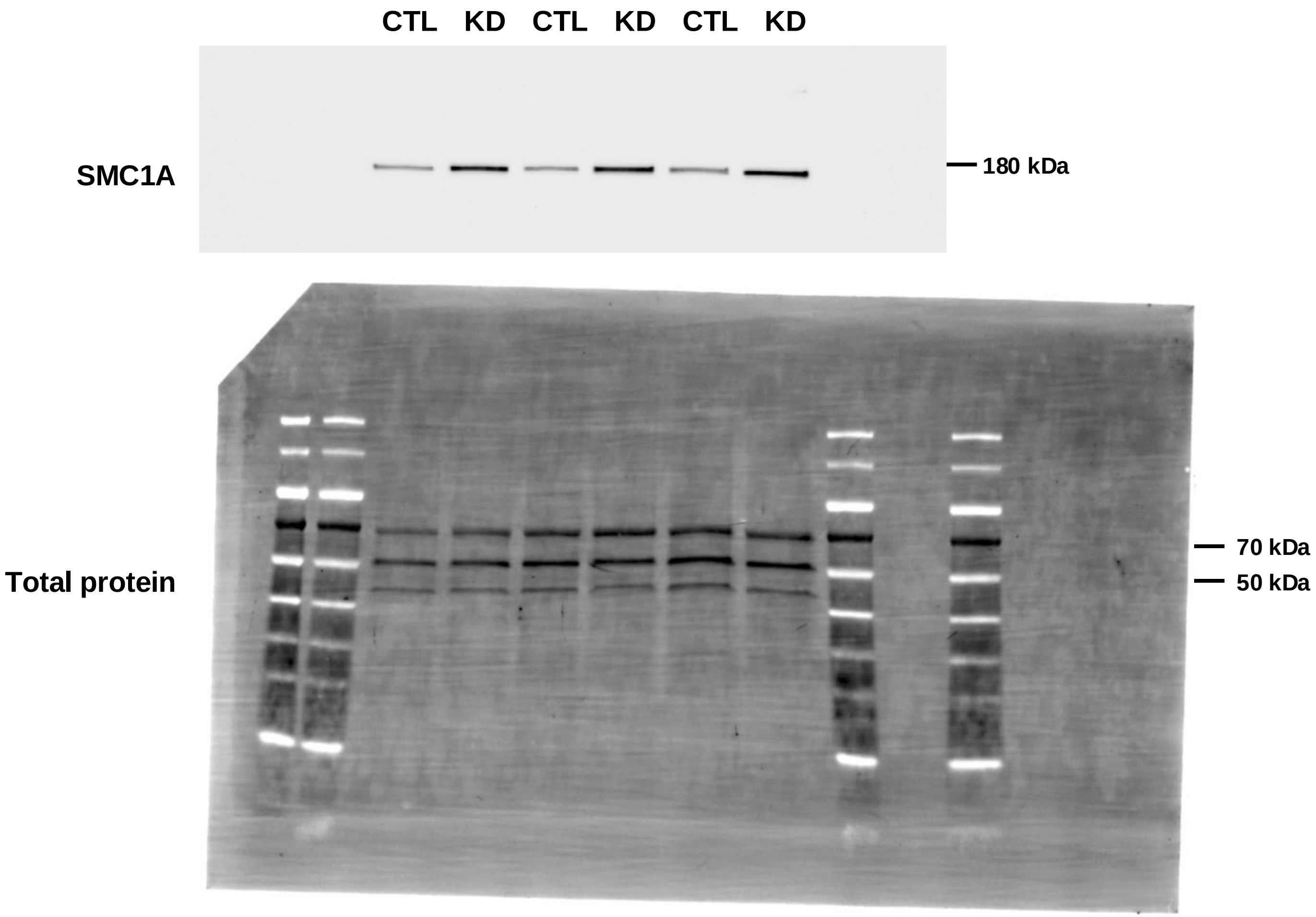

Full unedited blots for Figure 4E. All bands, excluding molecular weight ladders, were included in Figure 4E.

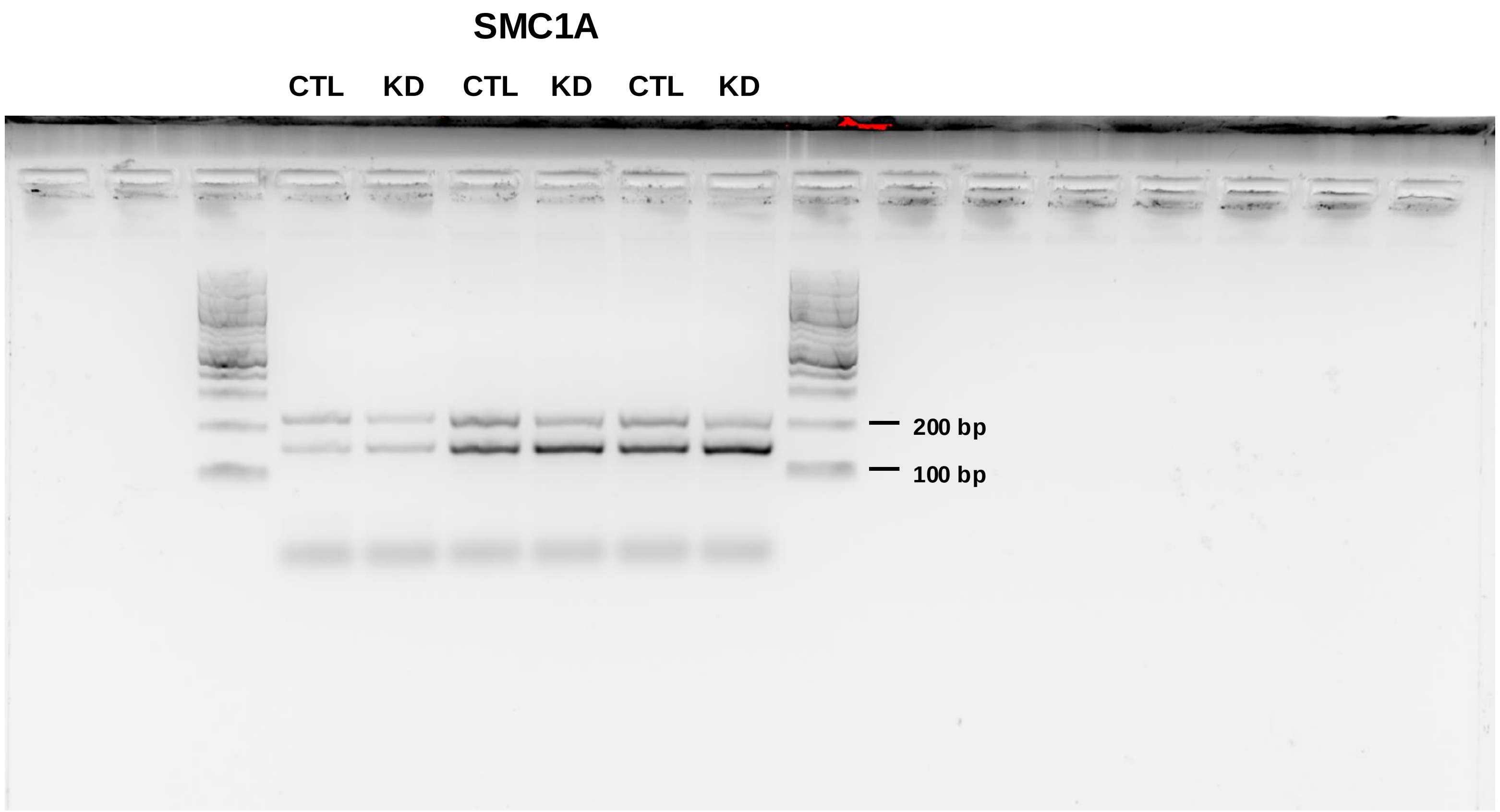

Full unedited gel for Figure 4F. All bands, excluding DNA ladders and primer dimers, were used in Figure 4F.

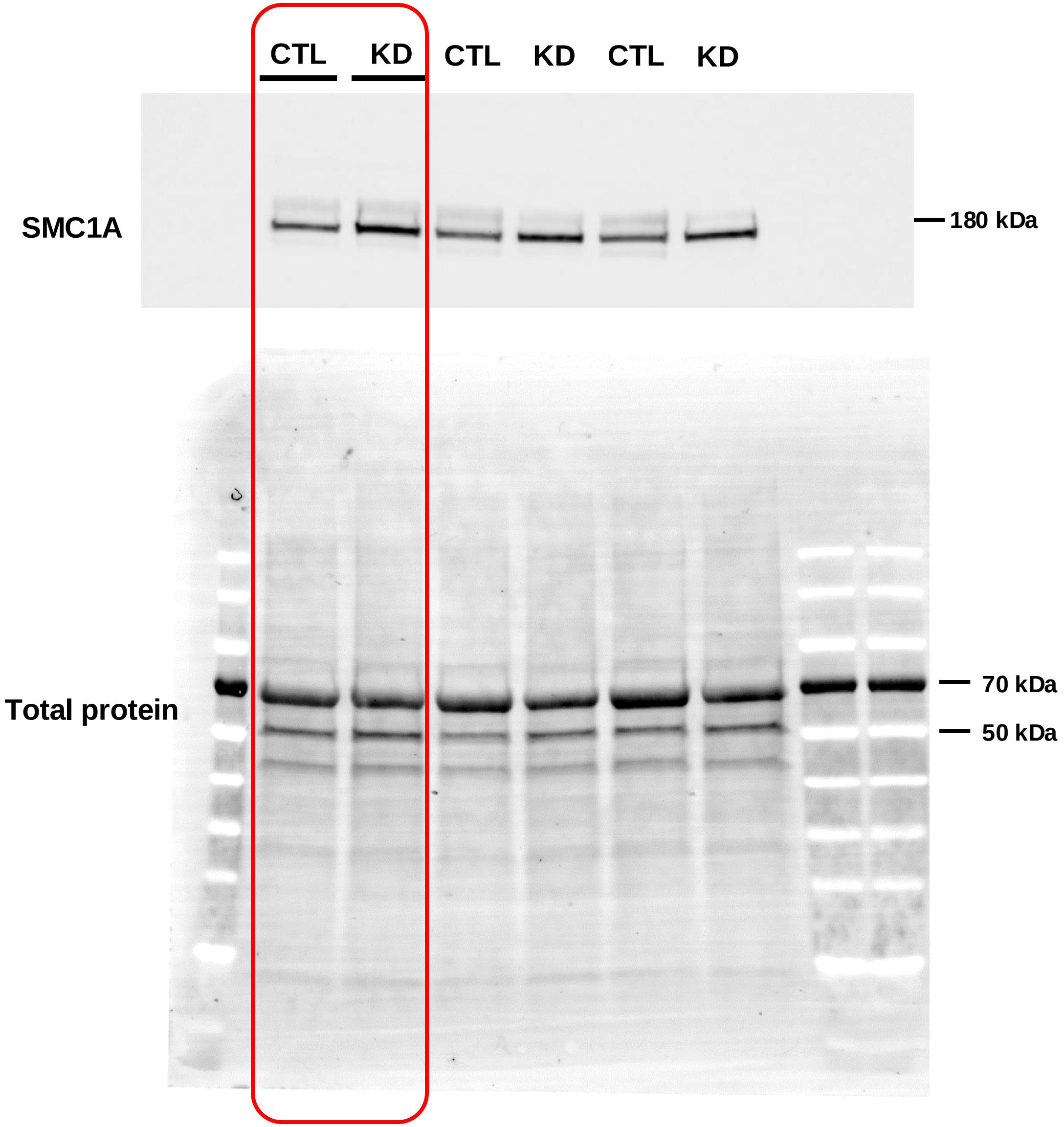

Full unedited blots for Figure 4H. Highlighted bands (red) were used in Figure 4H.

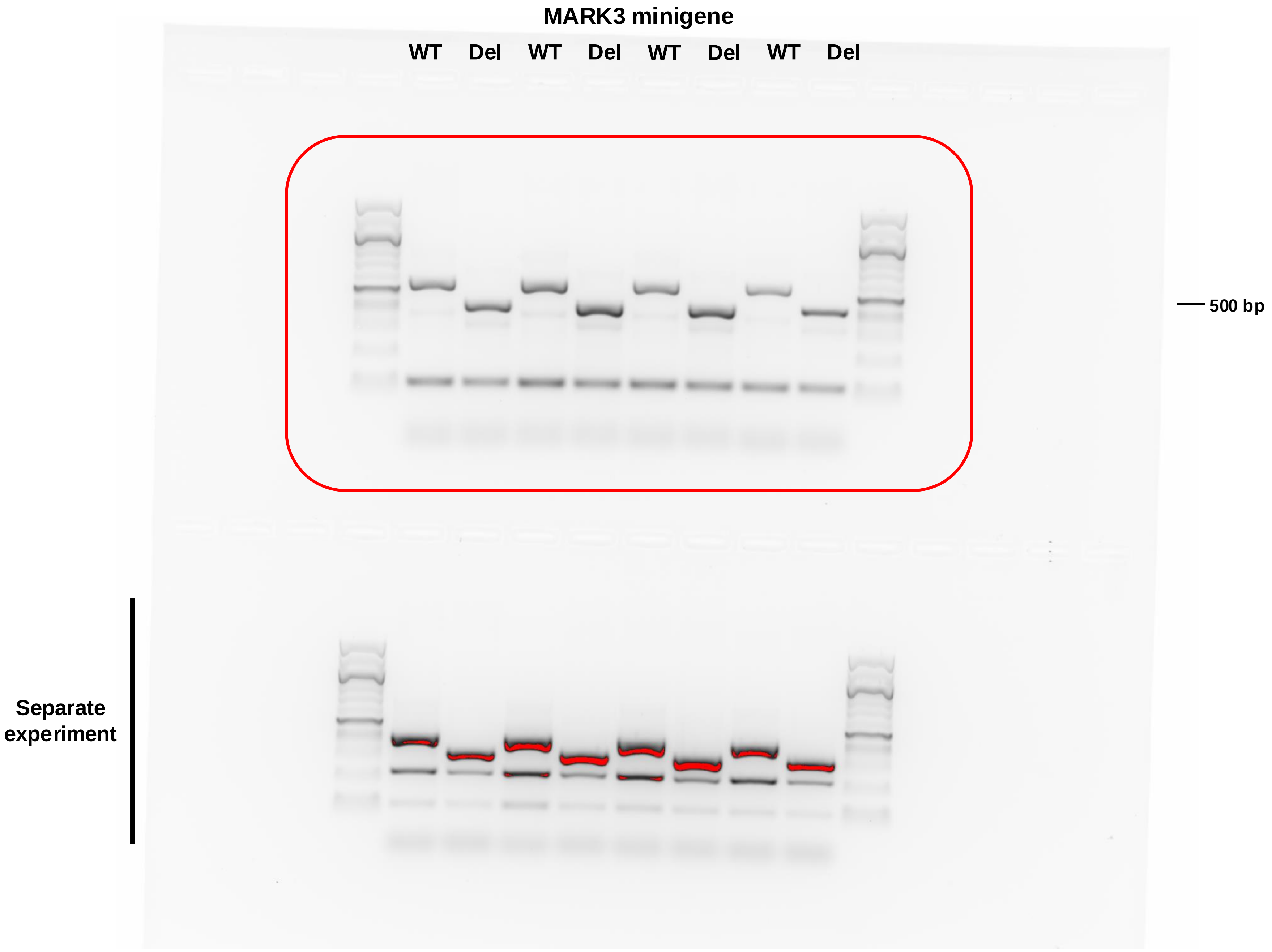

Full unedited gel for Figure 6E. Highlighted bands (red), excluding DNA ladders and primer dimers, were used in Figure 6E.

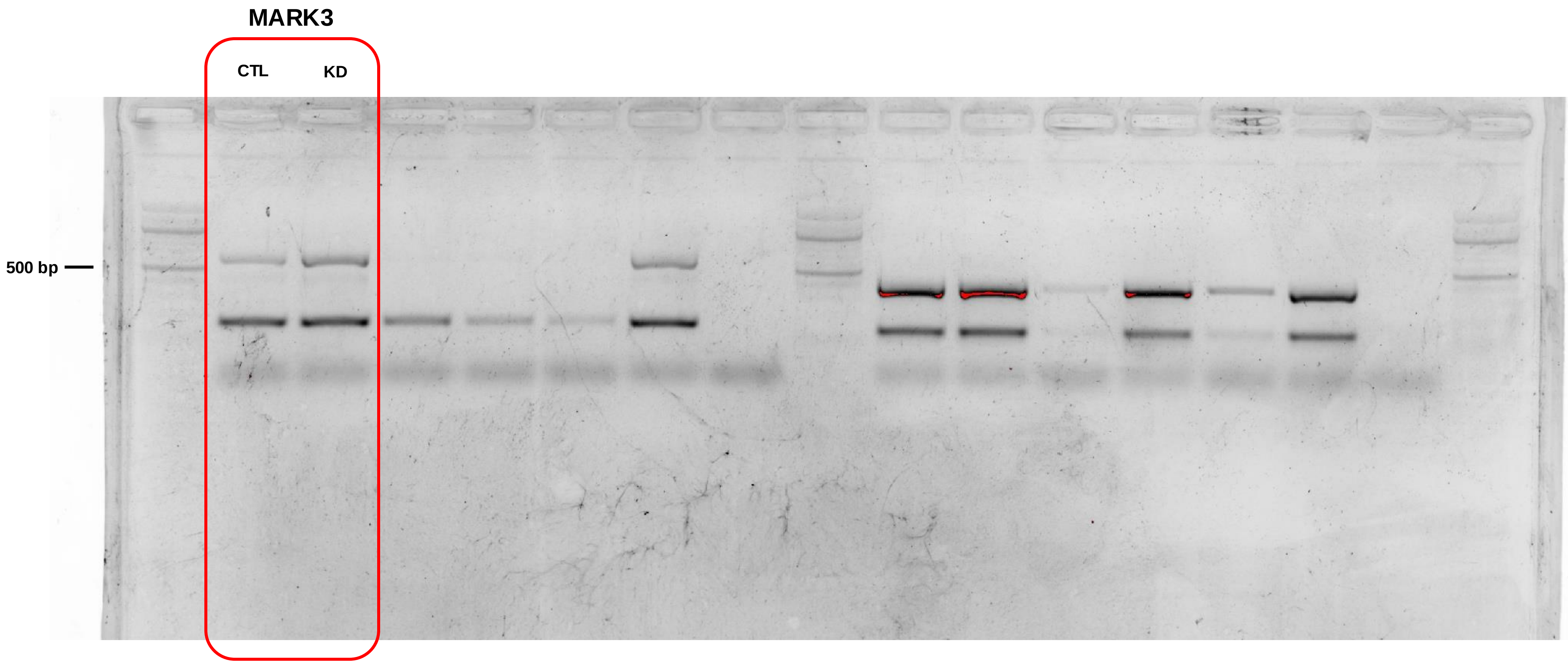

Full unedited gel for Figure 6F. Highlighted bands (red), excluding DNA ladders and primer dimers, were used in Figure 6F.

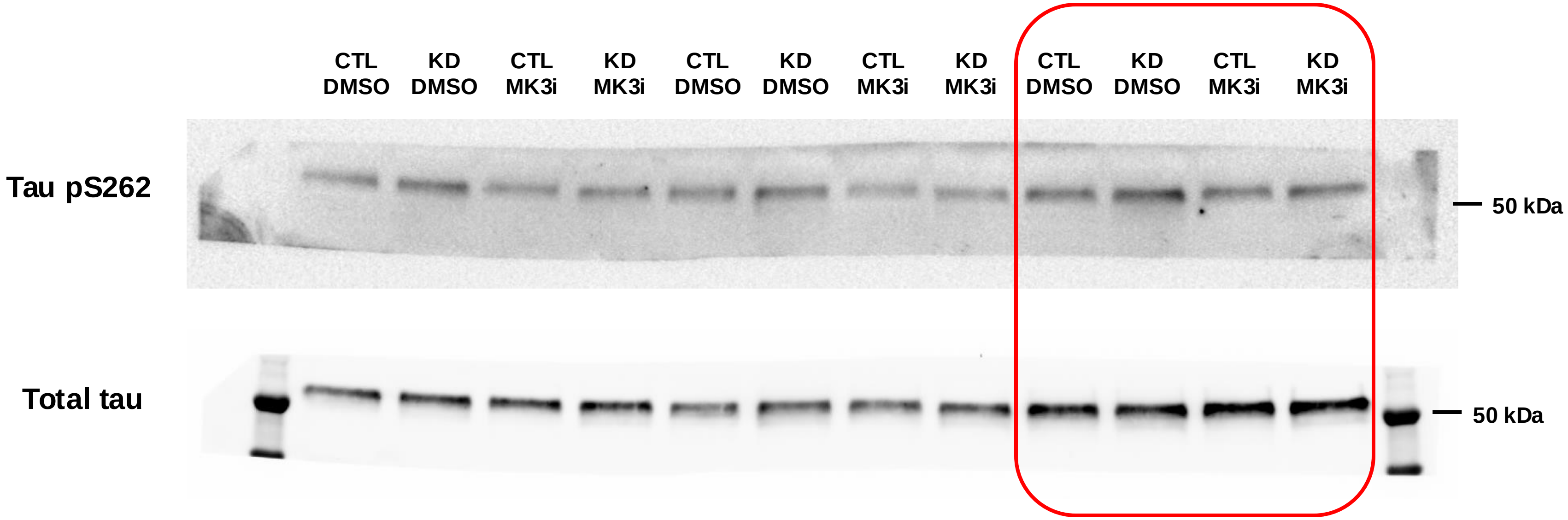

Full unedited blots for Figure 7D. Highlighted bands (red) were used in Figure 7D.

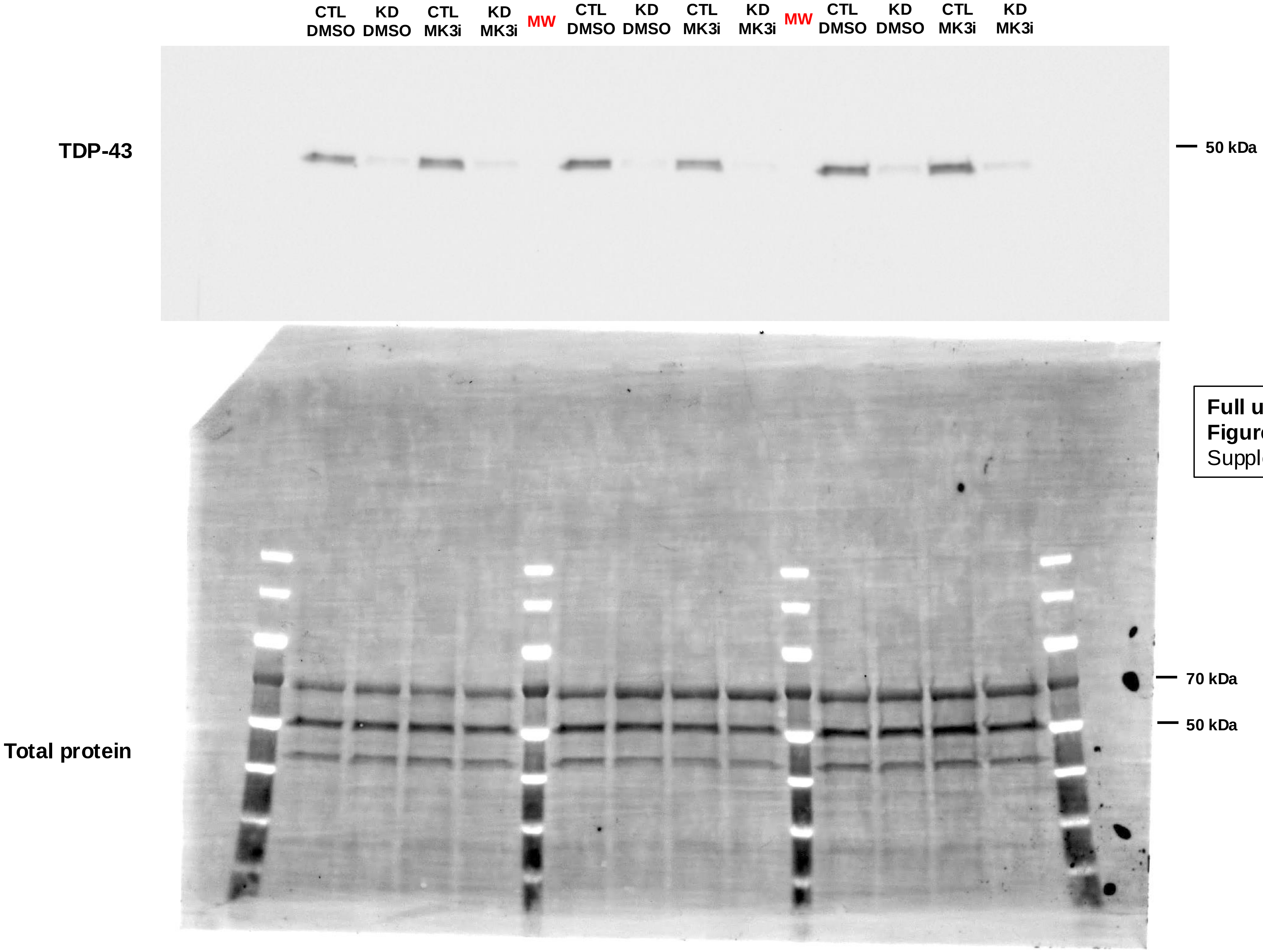

Full unedited blots for Supplemental Figure 6A. All bands were included in Supplemental Figure 6A.

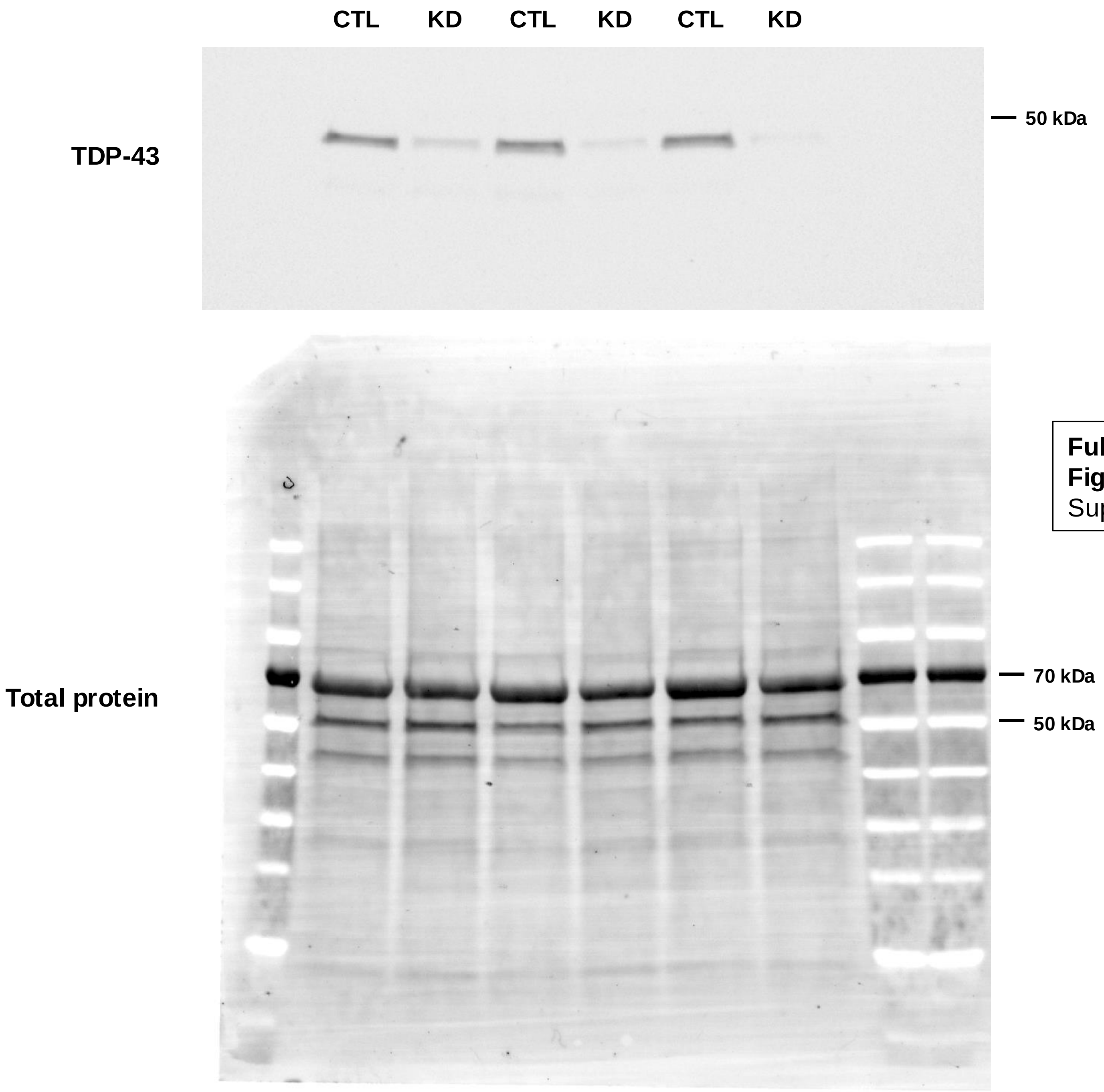

Full unedited blots for Supplemental Figure 6B. All bands were included in Supplemental Figure 6B.

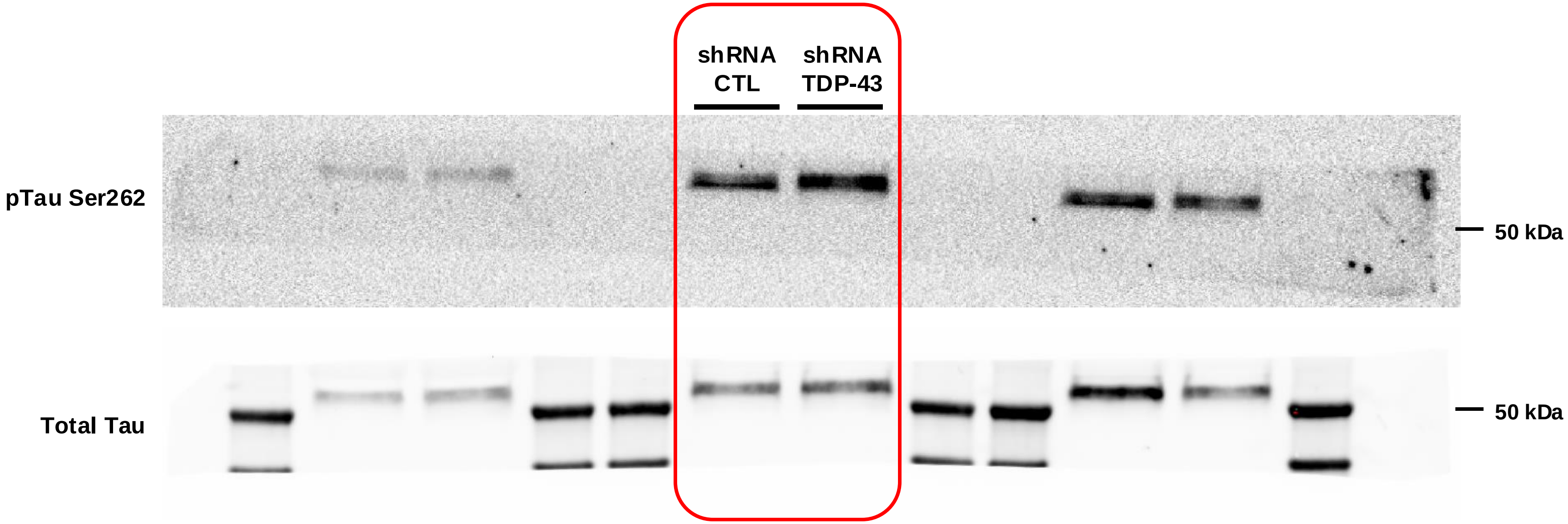

Full unedited blots for Supplemental Figure 10A. Highlighted bands (red) were used in Supplemental Figure 10A.

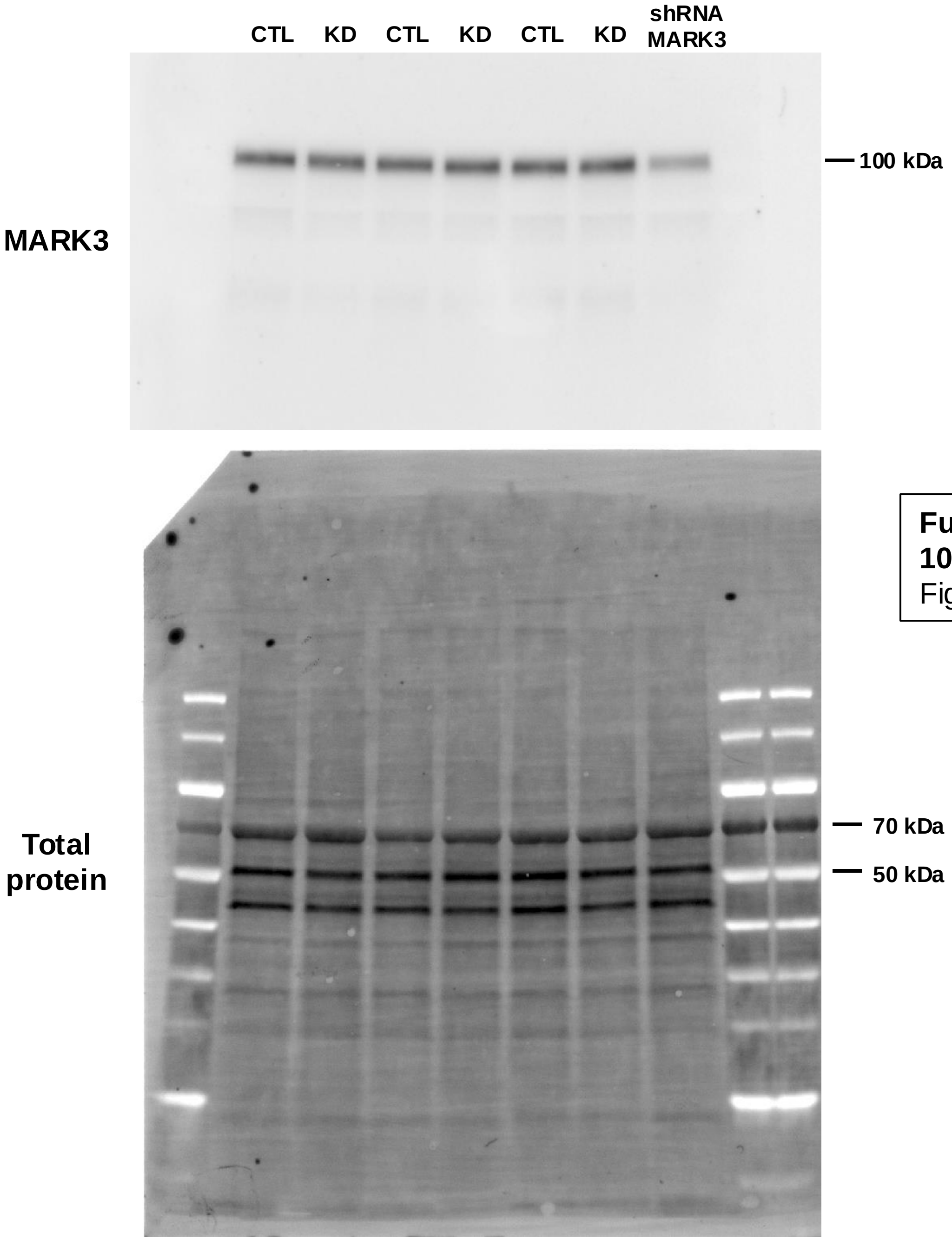

Full unedited blots for Supplemental Figure 10B. All bands were included in Supplemental Figure 10B.

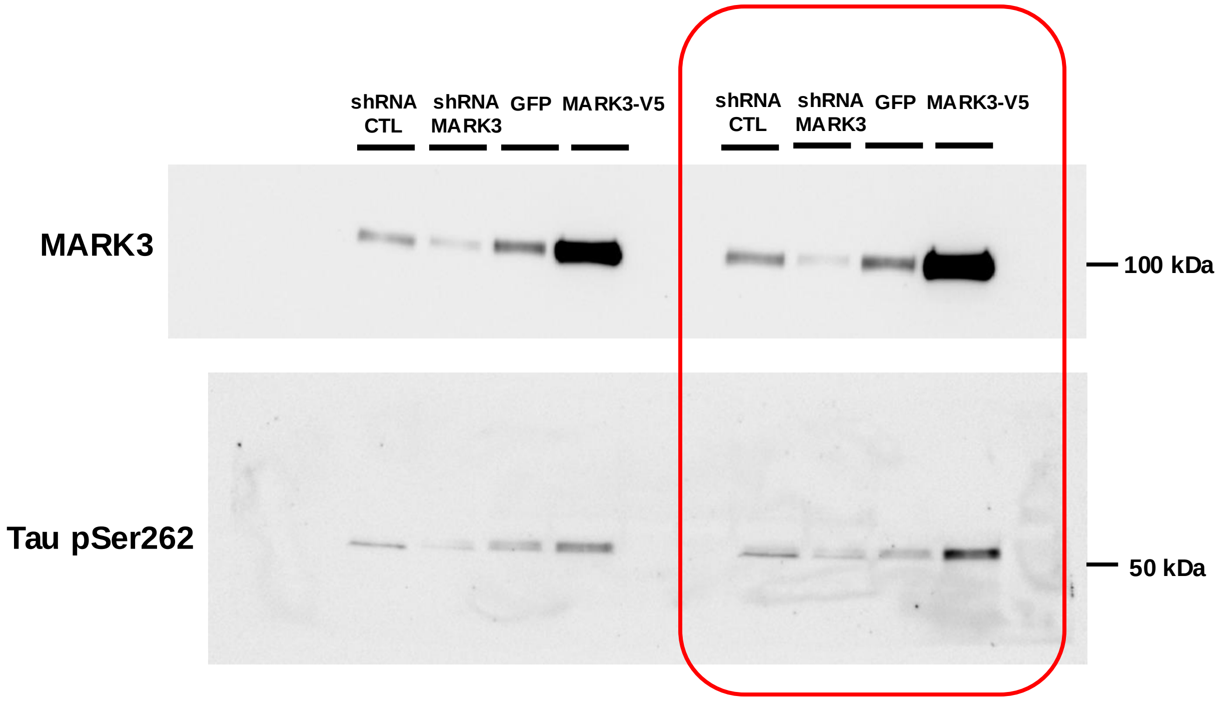

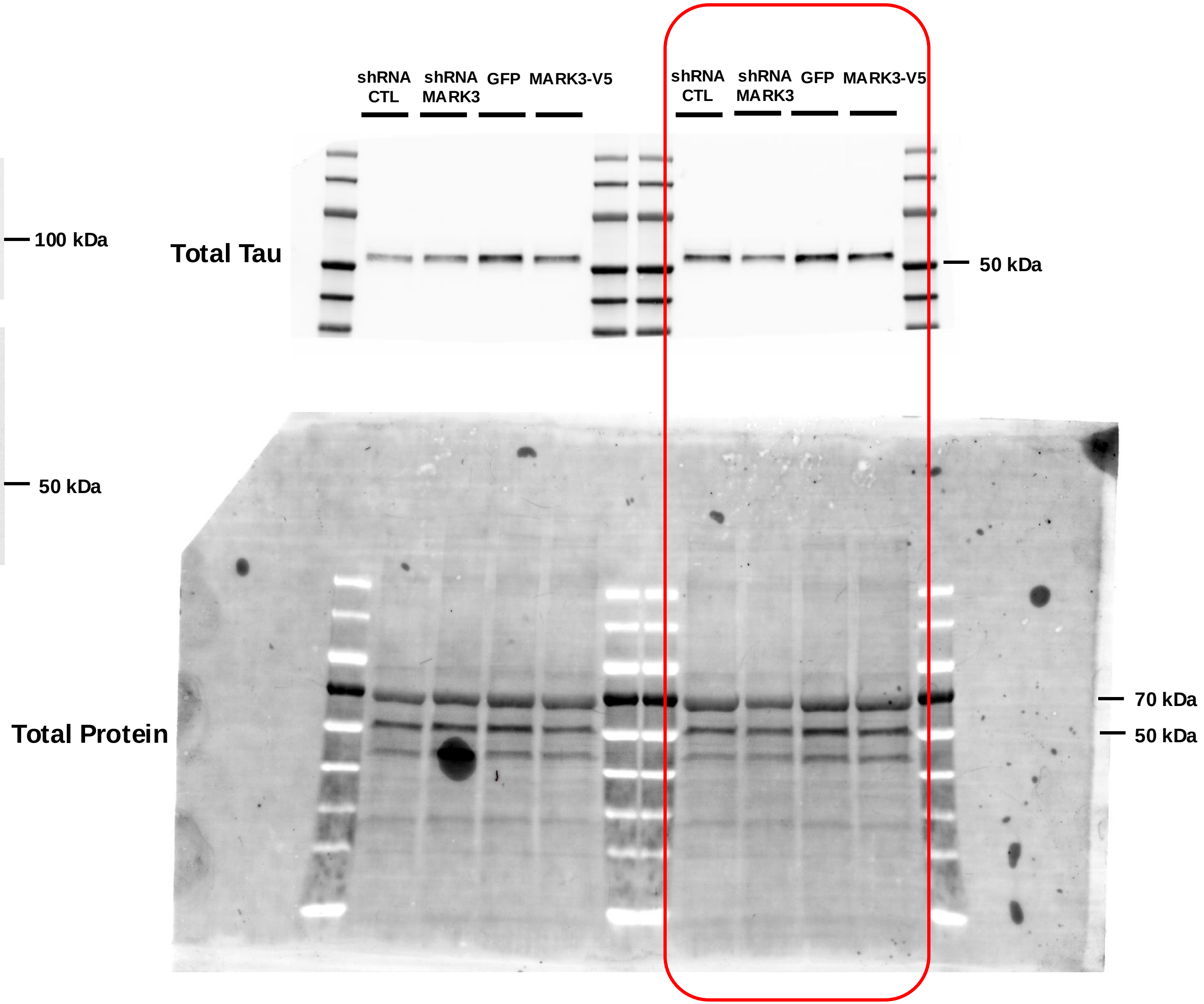

Full unedited blots for Supplemental Figure 10C. Highlighted bands (red) were used in Supplemental Figure 10C.

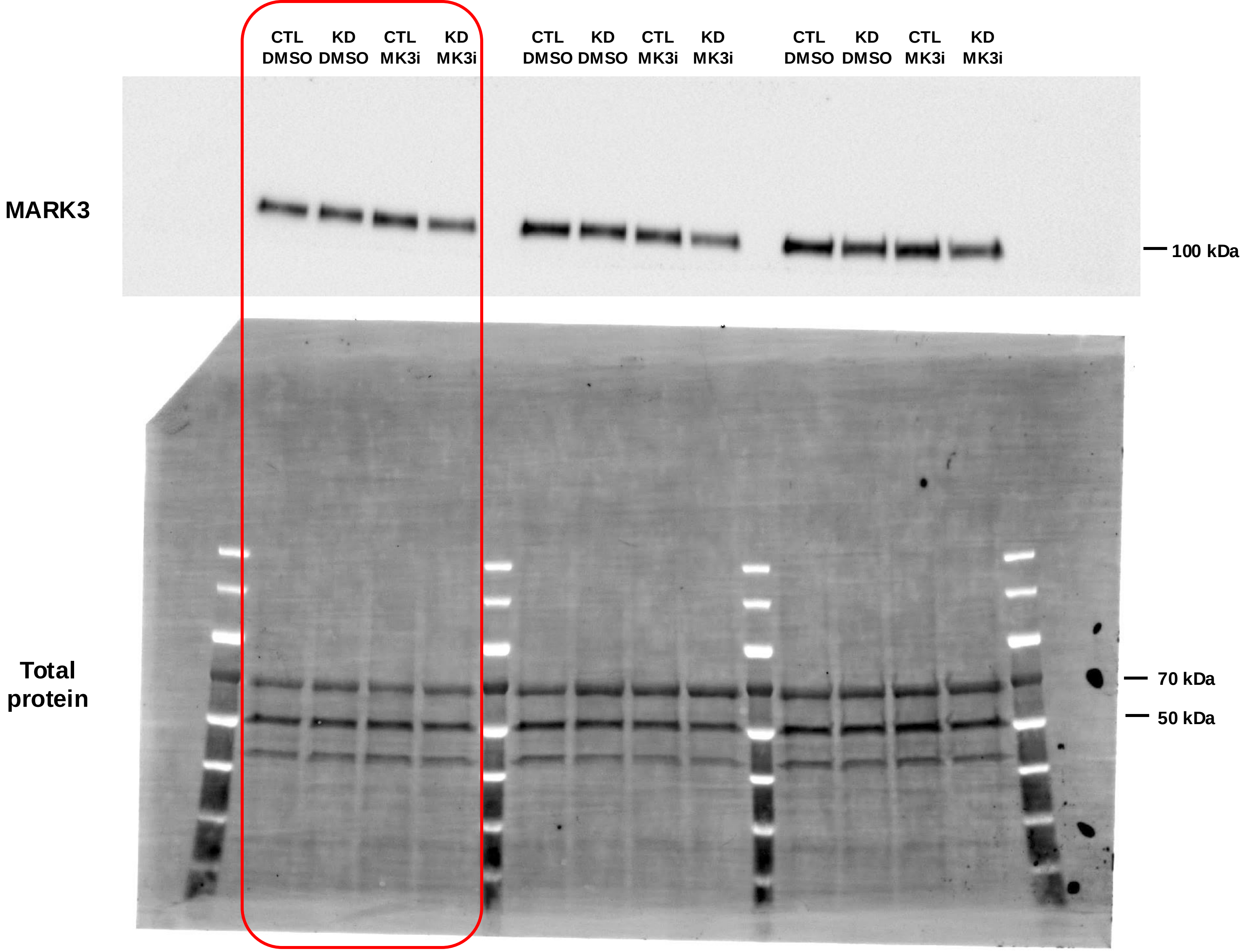

Full unedited blots for Supplemental Figure 11A. Highlighted bands (red) were used in Supplemental Figure 11A.

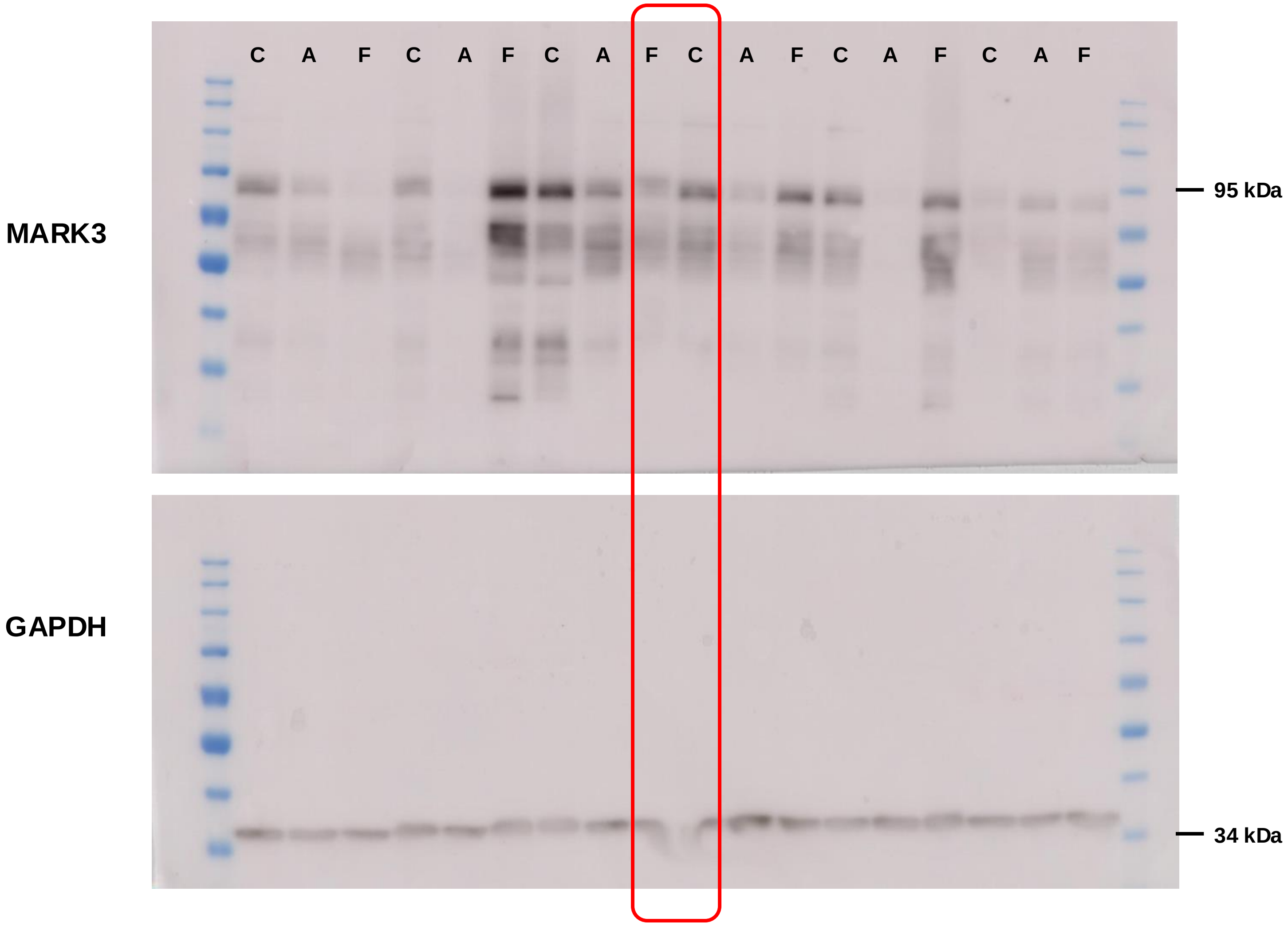

Full unedited blots for Supplemental Figure 11B. Highlighted bands (red) were excluded from the analysis represented in Supplemental Figure 11B due to smearing of the GAPDH signal.
C = healthy controls, A = ALS/FTD, F = FTLD-TDP

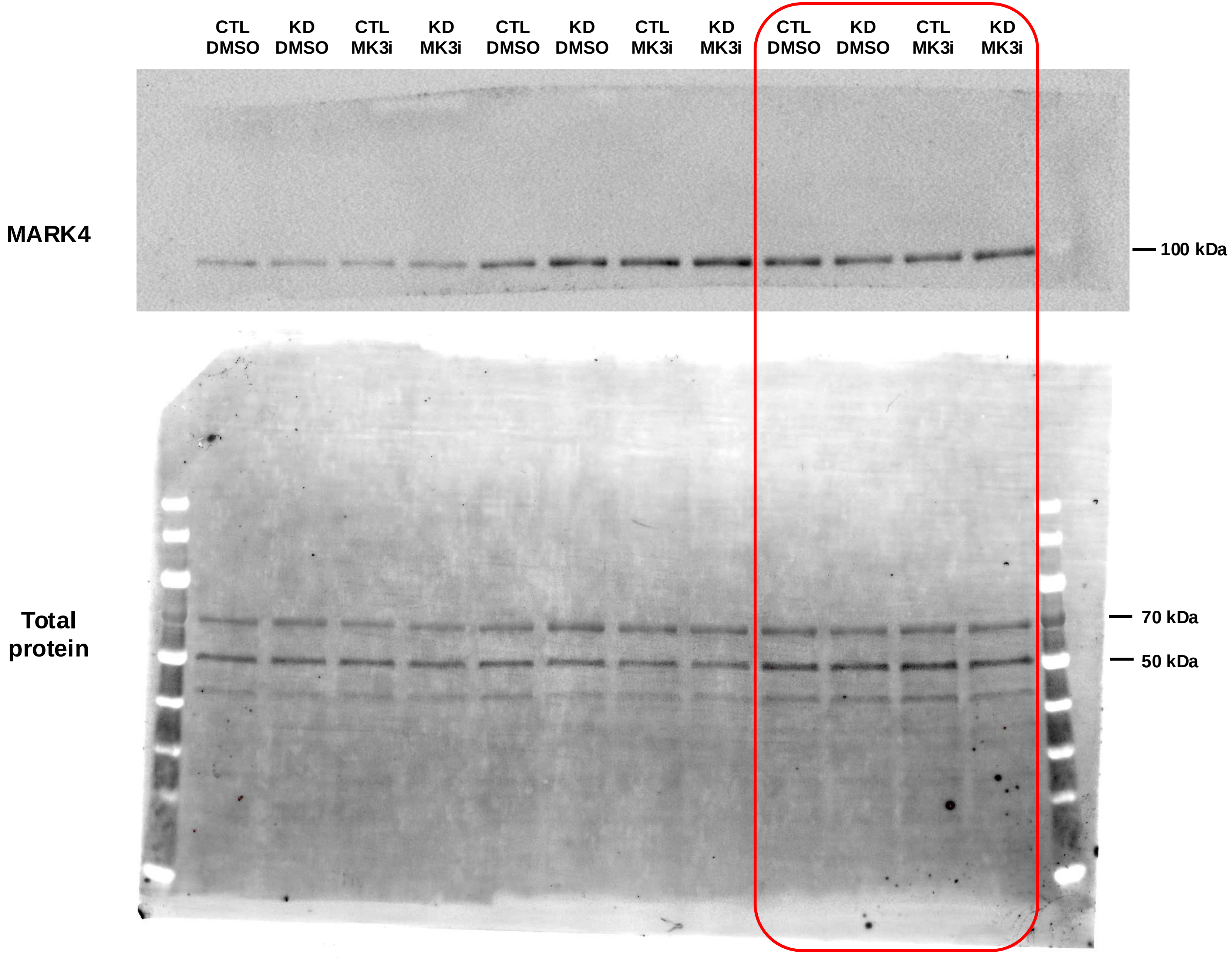

Full unedited blots for Supplemental Figure 11C. Highlighted bands (red) were used in Supplemental Figure 11C.
